# Synopsis of the cyclocephaline scarab beetles (Coleoptera, Scarabaeidae, Dynastinae)

**DOI:** 10.3897/zookeys.745.23683

**Published:** 2018-03-22

**Authors:** Matthew R. Moore, Ronald D. Cave, Marc A. Branham

**Affiliations:** 1 Department of Entomology and Nematology, University of Florida, Building 1881 Natural Area Drive, Steinmetz Hall, Gainesville, FL 32611, USA; 2 Department of Entomology and Nematology, University of Florida, Indian River Research and Education Center, 2199 South Rock Road, Fort Pierce, FL 34945, USA

**Keywords:** masked chafers, rhinoceros beetles, identification key

## Abstract

The cyclocephaline scarabs (Scarabaeidae: Dynastinae: Cyclocephalini) are a speciose tribe of beetles that include species that are ecologically and economically important as pollinators and pests of agriculture and turf. We provide an overview and synopsis of the 14 genera of Cyclocephalini that includes information on: 1) the taxonomic and nomenclatural history of the group; 2) diagnosis and identification of immature life-stages; 3) economic importance in agroecosystems; 4) natural enemies of these beetles; 5) use as food by humans; 6) the importance of adults as pollination mutualists; 7) fossil cyclocephalines and the evolution of the group; 8) generic-level identification of adults. We provide an expanded identification key to genera of world Cyclocephalini and diagnoses for each genus. Character illustrations and generic-level distribution maps are provided along with discussions on the relationships of the tribe’s genera.

## Introduction

The cyclocephaline scarabs (Coleoptera: Scarabaeidae: Dynastinae) are remarkable among rhinoceros beetles for the group’s immense species richness and ecological importance. Cyclocephalini is a pan-tropical tribe with several genera considered to be keystone pollinators in New and Old World tropical ecosystems. By one estimate, pollination mutualisms between cyclocephalines and early-diverging angiosperms suggest that nearly 900 species of Neotropical plants rely upon these scarab beetles for sexual reproduction (Schatz 1990). Beyond tropical forests, cyclocephaline scarab beetle species are important to human industry as pests in tropical and temperate agroecosystems and turfgrass in North America. Due to these factors, the group has received considerable alpha-taxonomic attention as species identity (and identification) is crucial for understanding the fascinating biology of these scarabs. However, almost nothing is known about the evolution of the group into their incredible ecological roles.

This paper synthesizes all available information on cyclocephaline scarab beetles into these broad categories: 1) taxonomic and nomenclatural history of the group organized by major worker, including an exegesis of Endrődi’s German-language revision of the tribe; 2) state of knowledge surrounding diagnosis and identification of immature life-stages; 3) economic importance in agroecosystems; 4) natural enemies of these beetles; 5) use as food by humans; 6) importance of adults as pollination mutualists; 7) knowledge of the fossil record and evolution; and 8) an overview of each genus, including expanded diagnoses and a key to world genera of Cyclocephalini.

## Nomenclatural and taxonomic history of the cyclocephaline scarabs (Scarabaeidae, Dynastinae, Cyclocephalini)

### Carl Linnaeus and his students

The taxonomic and nomenclatural history of Cyclocephalini traces to the works of Carl Linnaeus and several of his students. The 12^th^ edition of *Systema Naturae* included the description of *Scarabaeus amazonus* Linnaeus, 1767, which was later designated as the type species of *Cyclocephala* Dejean (Linnaeus 1767, Casey 1915, Endrődi 1966). This was the only cyclocephaline species described by Linnaeus. The short Latin description of *S. amazonus* indicated that this beetle was from “Suriname,” was smaller than many dung beetles (with a relatively shorter pronotum), and was testaceous with longitudinal, black stripes (Linnaeus 1767). Unfortunately, the type specimen of *S. amazonus* is apparently lost. A serious effort to find this Linnaean type was undertaken by Sebő Endrődi and fellow Coleopterist Bengt-Oloft Landin.

Landin, an expert in Linnaean scarabaeoid types (e.g., see Landin 1956), was in correspondence with Endrődi during the early phases of the latter’s revisionary works (Endrődi 1966). They determined that the type specimen of *S. amazonus* was not present in any of the museums that housed parts of the Linnaeus beetle collection: The De Geer collection at Naturhistoriska Riksmuseet (Stockholm, Sweden), Uppsala University, Museum of Evolution, Zoology Section (Uppsala, Sweden), and The Natural History Museum (London, United Kingdom). In a personal correspondence with Endrődi, Landin speculated that the specimen that became the type of *S. amazonus* was passed from Daniel Rolander (an apostle of Linnaeus sent to Suriname), then to Baron Charles De Geer, and eventually to Linnaeus (Endrődi 1966).

Two female specimens identified as *Melolontha amazona* (Linnaeus) from “Jamaic” and “Columbia” were found in the Schönherr collection at Naturhistoriska Riksmuseet (Endrődi 1966). The specimen from “Jamaic” was determined to be consistent with the description of *Melolontha signata* Fabricius, 1781, also from Jamaica (Endrődi 1966). The specimen from “Columbia” was determined to be conspecific with the mainland species *Cyclocephala detecta* Bates, 1888 (a synonym of *C. amazona*) (Endrődi 1966). This convinced Endrődi that the names *S. amazonus* and *M. signata* referred to the same species with continental and West Indian populations, respectively. Endrődi designated a neotype for *S. amazonus* from Paramaribo, Suriname in his collection (now deposited at Magyar Természettudományi Múzeum Allatatara [Hungarian Natural History Museum], Budapest, Hungary).

Johan Christian Fabricius described 11 species of cyclocephaline scarabs that were ultimately classified in the genera *Cyclocephala*, *Chalepides* Casey, *Dyscinetus* Harold, *Stenocrates* Burmeister, and *Ruteloryctes* Arrow (Fabricius 1775, 1781, 1787, 1798, 1801). Fabricius (1798) reported the earliest floral association record for Cyclocephalini when he noted that *Melolontha morio* Fabricius (=*Ruteloryctes morio*) was found in “*Nympheae floribus*” in “*India orientalis*.” This early floral association record was later validated, and *R. morio* is indeed a pollinator of the water lily, *Nymphaea lotus* L., in Benin, Côte d’Ivoire, Nigeria, and Senegal (Ervik and Knudsen 2003, Hirthe and Porembski 2003, Krell et al. 2003). Linnaeus’ students Leonard Gyllenhal and Carl Peter Thunberg combined to describe four cyclocephaline species later classified in *Cyclocephala* and *Stenocrates* (Thunberg 1814, Gyllenhal 1817a, b).

### Pierre François Marie Auguste Dejean and Pierre André Latreille

Dejean (1821) authored the genus *Cyclocephala* in the first edition of the catalog of his collection. There was longstanding confusion in the literature surrounding the proper authorship of the genus *Cyclocephala*, with most historical workers crediting the genus to Latreille (1829) (e.g., Arrow [1937b], Blackwelder [1944], and Endrődi [1966, 1985a]). This confusion stemmed from Dejean’s practice of proposing new genera without describing them in the catalogs of his collection (Bousquet and Bouchard 2013a, b). Dejean (1821) also attributed authorship to other workers who had applied names to species in their own collections, but before the names were formally described in the literature. Thus, subsequent authors treated Dejean’s new genera and species as invalid *nomina nuda*. However, because Dejean (1821) included one or more available species-group names in *Cyclocephala*, the genus-group name became available from that work (ICZN Article 12.2.5; see Bousquet and Bouchard 2013a for further discussion).

The following originally included available names were placed in *Cyclocephala* by Dejean (1821): *Melolontha geminata* Fabricius, 1801 (=*Dyscinetus dubius* [Olivier, 1789]), *Melolontha dubia* Olivier, 1789 (=*Dyscinetus dubius* [Olivier]), *Scarabaeus barbatus* Fabricius, 1787 (=*Chalepides barbatus* [Fabricius]), *Melolontha signata* Fabricius, 1781 (=*Cyclocephala amazona amazona* [Linnaeus, 1767]), and *Melolontha biliturata* Gyllenhal, 1817 (=*Cyclocephala tridentata* [Fabricius, 1801]).

Dejean (1821) included five *species inquirenda* (indicated by a “?”) in *Cyclocephala*: *Melolontha pallens* Fabricius, 1798 (=*Cyclocephala amazona amazona* [Linnaeus, 1767]), *Melolontha ferruginea* Fabricius, 1801 (=*Cyclocephala immaculata ferruginea* (Fabricius, 1801), *Melolontha valida* Schönherr, 1817 (=*Cyclocephala castanea* [Olivier, 1789]), *Melolontha immaculata* Olivier, 1789 (=*Cyclocephala immaculata immaculata* [Olivier, 1789]), and *Melolontha castanea* Olivier, 1789 (=*Cyclocephala castanea* [Olivier, 1789]). These five *species inquirenda* were not originally included in *Cyclocephala* and are ineligible for type species fixation (ICZN Article 67.2.5).

The second and third editions of Dejean’s (1833, 1836b) catalog followed Latreille (1829) and recognized the genus *Chalepus* MacLeay. Three species previously included in *Cyclocephala* *sensu* Dejean (1821) were transferred into *Chalepus* in the second edition (Dejean 1833). Additional *nomina nuda* were included in these two genera: 19 *nomina nuda* in *Cyclocephala* and eight in *Chalepus* (Dejean 1833). Twenty-three *nomina nuda* were placed in *Cyclocephala* in the third edition of the catalog (Dejean 1836b). Many of Dejean’s (1821, 1833, 1836) *nomina nuda* were later validly described by subsequent authors (e.g., *Ancognatha scarabaeoides* Erichson and *Ancognatha ustulata* [Burmeister]).

*Cyclocephala* was first described and illustrated by Latreille (1829, 1837). Latreille’s (1829) short description of *Cyclocephala* utilized characters of the protarsal claws (unequal in size and cleft at the apex), labrum (visible anteriorly), body shape (ovoid with the head uncovered), elytra (weakly edged without significant lateral dilation), and mandibles (narrow, not strongly produced beyond clypeus, without a lateral sinus, and variably toothed). The genus was also considered variable enough to warrant subgeneric division into *Chalepus* and *Cyclocephala* (Latreille 1829). Figure plates illustrated a dorsal habitus of *Cyclocephala frontalis* Chevrolat, 1844 and the anatomy of the head, labrum, maxilla, and protarsus of *Cyclocephala geminata* (Fabricius) (=*Dyscinetus dubius* [Olivier]) (Latreille 1837). These illustrations are some of the earliest scientific depictions of the group.

### Francis de Laporte de Castelnau

Laporte (1840) was the first author to propose a tribal-level taxon for the cyclocephaline scarab beetles. This group, Cyclocephalites, was included along with Dynastites and Rutélites in the family Xylophiles (Laporte 1840). Cyclocephalites was not originally proposed in a Latinized form (see Smith 2006, Bouchard et al. 2011). However, because the name was subsequently Latinized by several authors (e.g., Cyclocephalidae by Burmeister [1847] and Imhoff [1856], and Cyclocephalinae by Bates [1888]) and was generally accepted, the family-group name is available from this work per ICZN Article 11.7.2. Cyclocephalites *sensu* Laporte (1840) was diagnosed by having the mandibles mostly covered by the clypeus and the labrum not extending anteriorly beyond the apex of the clypeus. Laporte included two divisions in Cyclocephalites. The first division, diagnosed by arched and hooked mandibles, included only *Cyclocephala geminata* (Fabricius, 1801) (=*Dyscinetus dubius* [Olivier, 1789]). The second division of *Cyclocephala* was diagnosed by having straight, truncate, or obtuse mandibular apices (Laporte 1840). This second division contained six species, and these are still classified in *Cyclocephala*.

### Hermann Burmeister

The German naturalist and entomologist Karl Hermann Konrad Burmeister made major contributions to dynastine scarab research in the mid-19^th^ century (Berg 1894). Burmeister’s (1844, 1847, 1855) *Handbuch der Entomologie* volumes systematically organized a large portion of Scarabaeoidea. Burmeister (1847) was one of the first authors to unite members of the subfamily Dynastinae, nearly as currently circumscribed, into a single family and recognizable tribes in the modern sense. This family, Xylophila, was subdivided into Cyclocephalidae, Phileuridae, Dynastidae, Agaocephalidae, Strategidae, Oryctidae, and Xylophila *amphibola* (=Scarabaeidae: Cetoniinae: Trichiini, in part) (Burmeister 1847). Seven of the genera included in Burmeister’s Cyclocephalidae are still part of Cyclocephalini (Table 1). Additionally, Burmeister described five new genera and 71 species-group taxa (56 of which are valid species or subspecies) that are still included in Cyclocephalini.

**Table 1.** Burmeister’s (1847) classification of genera of Cyclocephalidae.

Division	Genera	Current Tribal Classification
Cyclocephalidae *spurii*	*Pachylus* Burmeister, 1847 (=*Alvarengius* Frey, 1975)	Rutelinae: Alvarengiini
	*Hexodon* Olivier, 1789	Dynastinae: Hexodontini
Oryctomorphidae	*Democrates* Burmeister, 1847	Dynastinae: Agaocephalini
	*Oryctomorphus* Guérin-Méneville, 1831	Rutelinae: Rutelini
	*Homoeomorphus* Burmeister, 1847	Dynastinae: Pentodontini
Cyclocephalidae *genuini*	*Augoderia* Burmeister, 1847	Dynastinae: Cyclocephalini
	*Cyclocephala* Dejean, 1821	Dynastinae: Cyclocephalini
	*Harposceles* Burmeister, 1847	Dynastinae: Cyclocephalini
Chalepidae	*Erioscelis* Burmeister, 1847	Dynastinae: Cyclocephalini
	*Bradyscelis* Burmeister, 1847 (=*Oryctoderus* Boisduval, 1835)	Dynastinae: Oryctoderini
	*Peltonotus* Burmeister, 1847	Dynastinae: Cyclocephalini
	*Chalepus* MacLeay, 1819 (=*Dyscinetus* Harold 1869 in part, *Chalepus* also contained species currently classified in *Chalepides* Casey, 1915)	Dynastinae: Cyclocephalini
	*Stenocrates* Burmeister, 1847	Dynastinae: Cyclocephalini

Cyclocephalidae *sensu* Burmeister included 13 genera placed in four divisions. Two of these divisions, Cyclocephalidae *spurii* and Oryctomorphidae, included genera that are all currently classified in Rutelinae and various other dynastine tribes (Table 1) (Burmeister 1847, Ohaus 1929, Endrődi 1966, 1985a, Frey 1975). Cyclocephalidae *genuini* was the most species-rich of Burmeister’s divisions. This group contained three genera: *Augoderia*, *Cyclocephala*, and *Harposceles*. Burmeister (1847) described more than 50 new taxa in *Cyclocephala* and treated 70 species in the genus. *Cyclocephala* was further organized into eight species groups based largely on head morphology: *Cyclocephalae anomalinae*, *Cyclocephalae acutae*, *Cyclocephalae parabolicae*, *Cyclocephalae heterocerae*, *Cyclocephalae reflexae*, *Cyclocephalae microcephalae*, *Cyclocephalae sinuatae*, and *Cyclocephalae eurycephalae*. These *Cyclocephala* species-groups were never formalized, but they were discussed by Lacordaire (1856) and Endrődi (1966).

### Henry Walter Bates

Famous English naturalist Henry Walter Bates treated cyclocephalines in his contributions to the scientific opus *Biologia Centrali-Americana* and Edward Whymper’s *Travels Amongst the Great Andes of the Equator* (Bates 1888, 1891). Between these two works, Bates covered over 50 cyclocephaline species-level taxa, described nearly 30 new species (20 of which are still accepted as valid), and contributed to the generic-level classification of the group. For example, he recognized the distinctiveness of *Ancognatha* Erichson 1847 and revalidated the genus, which had been synonymized with *Cyclocephala* (Erichson 1847, Lacordaire 1856, Bates 1888). He described two new cyclocephaline genera: *Aspidolea* Bates 1888 and the eventual junior synonym *Barotheus* Bates, 1891 (=*Ancognatha* Erichson).

Following Lacordaire’s (1856) system, Bates classified the cyclocephaline scarab beetles as a subfamily (Cyclocephalinae) within Dynastidae. He only provided diagnoses for two higher groups (what he called “subtribes” within Lamellicornia) based upon labial morphology. Thus, Bates did not propose a character-based circumscription of the cyclocephaline scarabs or dynastines more broadly. However, some of the earliest detailed discussion and comparison of generic-level diagnostic characters among cyclocephalines can be found in *Biologia Centrali-Americana* (Bates 1888). For example, the toothless (or nearly toothless) maxillary galeae of *Aspidolea* and *Ancognatha* were recognized as providing partial justification for accepting these genera as being distinct from *Cyclocephala* (Bates 1888).

Bates (1888) divided *Cyclocephala* into a series of informal species-groups. For example, group I, which contained *C. signata* Fabricius (=*C. amazona*) was diagnosed by: 1) an elongated or protracted clypeus; 2) the clypeal apex sometimes bent at the margin; and, 3) the apex of the ligula deeply divided and widely splayed (Bates 1888). Similar diagnoses that relied upon a combination of clypeal and labial morphology were provided for five major *Cyclocephala* species-groups. Sexual dimorphism of the antennal club (elongated in males) was used to further subdivide one of these species-groups (Bates 1888). Bates also covered the cyclocephaline genera *Dyscinetus* and *Stenocrates*. With less available material, he was unable to make many meaningful character comparisons for these genera. However, he did mention that the dorsoventrally flattened tibiae of *Stenocrates* serve to diagnose that genus (Bates 1888).

### Thomas Lincoln Casey, Jr.

Lieutenant Colonel Thomas Casey’s major contribution to scarabaeology was the sixth volume of *Memoirs on the Coleoptera* (Casey 1915). This volume covered Cetoniinae, Rutelinae, and Dynastinae of Central and North America. It provided keys to tribes, genera, and species, reported distributional data, and served as an outlet for the description of many new taxa. Casey (1909, 1915) treated Cyclocephalini as a tribe of Dynastinae, and he was the first Coleopterist to propose extensive generic-level reorganization of the tribe and the genus *Cyclocephala*. Most of Casey’s new taxa (genera, species, and subspecies) in Cyclocephalini were not accepted as valid by subsequent workers. For example, Casey described over 60 new species and subspecies of cyclocephaline scarabs. Only seven of these taxa are currently accepted as valid. Casey (1915) proposed 16 new genera and subgenera in Cyclocephalini, among which only *Chalepides* Casey is currently in use (Table 2). Casey (1915) was the first author to definitively place *Anoplocephalus* Schaeffer, 1906 (=*Coscinocephalus* Prell, 1936) in Cyclocephalini.

**Table 2.** Casey’s (1915) new cyclocephaline genera and subgenera.

Genus or subgenus	Type species	Status of genus or subgenus
*Mononidia* Casey, 1915	*Cyclocephala carbonaria* Arrow, 1911, by monotypy	Synonym of *Cyclocephala* Dejean
*Stigmalia* Casey, 1915	*Cyclocephala mafaffa* Burmeister, 1847, by original designation	Synonym of *Cyclocephala* Dejean
*Mimeoma* Casey, 1915	*Cyclocephala maculata* Burmeister, 1847, by monotypy	Synonym of *Cyclocephala* Dejean
*Diaptalia* Casey, 1915	*Cyclocephala discicollis* Arrow, 1902, by monotypy	Synonym of *Cyclocephala* Dejean
*Spilosota* Casey, 1915	*Spilosota nubeculina* Casey, 1915, by original designation	Synonym of *Cyclocephala* Dejean
Ochrosidia (Ochrosidia) Casey, 1915	*Melolontha immaculata* Olivier, 1789, by original designation	Synonym of *Cyclocephala* Dejean
Ochrosidia (Graphalia) Casey, 1915	not yet designated	Synonym of *Cyclocephala* Dejean
*Dichromina* Casey, 1915	*Cyclocephala dimidiata* Burmeister, 1847, by original designation	Synonym of *Cyclocephala* Dejean
*Homochromina* Casey, 1915	*Homochromina divisa* Casey, 1915, by original designation	Synonym of *Cyclocephala* Dejean
*Halotosia* Casey, 1915	*Cyclocephala fasciolata* Bates, 1888, by monotypy	Synonym of *Cyclocephala* Dejean
*Aclinidia* Casey, 1915	*Melolontha castanea* Olivier, 1789, by monotypy	Synonym of *Cyclocephala* Dejean
Cyclocephala (Plagiosalia) Casey, 1915	*Cyclocephala complanata* Burmeister, 1847, by original designation	Synonym of *Cyclocephala* Dejean
Cyclocephala (Isocoryna) Casey, 1915	Cyclocephala (Iscoryna) jalapensis Casey, 1915, by monotypy	Synonym of *Cyclocephala* Dejean
Dyscinetus (Palechus) Casey, 1915	Dyscinetus (Palechus) histrio Casey, 1915, by original designation	Synonym of *Dyscinetus* Harold
Parachalepus (Parachalepus) Casey, 1915	*Scarabaeus barbatus* Fabricius, 1787, by original designation	Synonym of *Chalepides* Casey
Parachalepus (Chalepides) Casey, 1915	Parachalepus (Chalepides) eucephalus Casey, 1915, by original designation	Valid

### Gilbert John Arrow

English entomologist Gilbert Arrow was notable among early 20^th^ century workers for his global knowledge of Dynastinae and Rutelinae. Arrow’s work in The Natural History Museum allowed him to meaningfully compare characters between diverse New and Old World taxa. For example, the genus *Peltonotus* (considered by most authors to be a cyclocephaline since Burmeister) was transferred into Rutelinae based on the form of the labrum (chitinized apically and projected anteriorly beyond the apex of the clypeus), which it shares with several Asian, parastasiine-like genera (Arrow 1908, 1910). Arrow (1908) described the Afrotropical cyclocephaline genus *Ruteloryctes*, which he compared to the New World genus *Dyscinetus*.

Cyclocephalines, as currently circumscribed, were covered in 11 of Arrow’s publications (Arrow 1900, 1902, 1903, 1908, 1910, 1911, 1913, 1914, 1931, 1937a, b). Arrow described over 40 new species or subspecies of cyclocephalines, and most of these were in the genus *Cyclocephala*. An early critic of Casey’s (1915) genus and species concepts, Arrow (1937a) argued that many of Casey’s new dynastine taxa created unnecessary “disorder” in Cyclocephalini and the subfamily more broadly. Arrow attributed this upheaval to Casey’s ignorance of species that invalidated his generic diagnoses. For example, Arrow criticized Casey’s overreliance on geographic separation of taxa and his intolerance for intraspecific variation, specimen wear, and recognition of teratological forms as distinct taxa.

Arrow (1937b) published the first comprehensive catalog of Dynastinae since Gemminger and Harold’s *Catalogus Coleopterorum* (see Harold 1869b). By Arrow’s admission, incorporating Casey’s cyclocephaline taxa into this catalog was challenging. Arrow struggled to place most species within Casey’s (1915) generic and subgeneric framework or assign synonymy to many species. He generally listed Casey’s higher taxa as subgeneric-level synonyms within *Cyclocephala* (Arrow 1937a, b). *Mimeoma* was accepted by Arrow (1937b), and he included a second species in the genus. *Chalepides* was also accepted as valid, and he elevated the subgenus to genus status (Arrow 1937a, b). Arrow expanded the composition of Cyclocephalini (Table 3) to include several Australasian genera that were later transferred to Oryctoderini (Scarabaeidae: Dynastinae) (Endrődi 1966, 1971a). Some of these Australasian genera had been placed into Cyclocephalini at the time of their description (e.g., *Chalcocrates* Heller, 1903).

**Table 3.** The generic composition of Cyclocephalini *sensu* Arrow (1937b).

Genera	Biogeographic Realm	Current Tribal Classification
*Ancognatha* Erichson, 1847	Neotropical and Nearctic	Dynastinae: Cyclocephalini
*Aspidolea* Bates, 1888	Neotropical and Nearctic	Dynastinae: Cyclocephalini
*Augoderia* Burmeister, 1847	Neotropical	Dynastinae: Cyclocephalini
*Barotheus* Bates, 1891 (=*Ancognatha* Erichson)	Neotropical	Dynastinae: Cyclocephalini
*Chalcocrates* Heller, 1903	Australasia	Dynastinae: Oryctoderini
*Chalcosthenes* Arrow, 1937	Australasia	Dynastinae: Oryctoderini
*Chalepides* Casey, 1915	Neotropical	Dynastinae: Cyclocephalini
*Coenoryctoderus* Prell, 1933	Australasia	Dynastinae: Oryctoderini
*Coscinocephalus* Prell, 1936	Nearctic	Dynastinae: Pentodontini
*Cyclocephala* Dejean, 1821	Neotropical and Nearctic (established in Australia)	Dynastinae: Cyclocephalini
*Dyscinetus* Harold, 1869	Neotropical and Nearctic	Dynastinae: Cyclocephalini
*Erioscelis* Burmeister, 1847	Neotropical	Dynastinae: Cyclocephalini
*Harposceles* Burmeister, 1847	Neotropical	Dynastinae: Cyclocephalini
*Melanhyphus* Fairmaire, 1881	Australasia	Dynastinae: Oryctoderini
*Mimeoma* Casey, 1915	Neotropical	Dynastinae: Cyclocephalini
*Neohyphus* Heller, 1896	Australasia	Dynastinae: Oryctoderini
*Onychionyx* Arrow, 1914	Australasia	Dynastinae: Oryctoderini
*Oryctoderus* Boisduval, 1835	Australasia	Dynastinae: Oryctoderini
*Ruteloryctes* Arrow, 1908	Afrotropical	Dynastinae: Cyclocephalini
*Stenocrates* Burmeister, 1847	Neotropical	Dynastinae: Cyclocephalini

### Lawrence Saylor

American entomologist Lawrence Saylor authored five publications (Saylor 1936, 1937, 1945, 1946, 1948) that included cyclocephaline scarab beetles, especially focusing on North American species. Saylor’s publications were very important for the time because they offered high-quality diagnoses, keys, and illustrations for species of *Ancognatha*, *Cyclocephala*, *Dyscinetus*, and *Erioscelis*. Saylor’s approach and implied species concept arguably influenced Endrődi’s revision of the tribe (see Ratcliffe 2016 for further discussion). Saylor’s role was not as a describer of new species in the group, but rather as a primary reviser of many North American dynastine taxa that had been neglected since the works of John Lawrence LeConte (1854, 1861, 1862, 1863, 1866) and George Henry Horn (1871, 1875, 1894) and further obfuscated by Casey (1915). The problem of Casey’s numerous cyclocephaline synonyms also fell firmly on Saylor. Saylor (1937, 1945) synonymized over 30 of Casey’s taxa in *Cyclocephala* and *Dyscinetus*, which created more reliable and precise diagnoses of North American species in these genera.

### Antonio Martínez

Antonio Martínez was the most productive South American dynastine worker of the middle and late 20^th^ century. Martínez was the principal author or coauthor of 22 publications that covered Cyclocephalini (Martínez 1954, 1955, 1957, 1960a, b, 1964, 1965a, b, 1966, 1967, 1968a–c, 1969, 1975a, b, 1978a, b, D’Andretta and Martínez 1956, Bolívar y Pieltan et al. 1963, Martínez and Martínez 1981, Martínez and Morón 1984). These publications were outlets for the description of new taxa and distribution data from under-sampled areas of South America, especially from localities in Argentina, Bolivia, Brazil, Ecuador, Paraguay, Peru, and Venezuela. Martínez was an author of 25 cyclocephaline species and subspecies (23 of which are still valid) and four genera and subgenera. The genera *Arriguttia* Martínez, 1960 and *Surutu* Martínez, 1955 were accepted by subsequent authors. *Albridarollia* Bolívar y Pieltan, Jiménez-Asúa, and Martínez, 1963, which included two South American species, was synonymized with *Cyclocephala* (Endrődi 1964, 1966). The monotypic subgenus Paraclinidia Martínez, 1965 was also synonymized with *Cyclocephala* (Endrődi 1966).

### Sebő Endrődi

The Hungarian Sebő Endrődi, a lawyer by formal training, was the most prolific and important dynastine worker of the 20^th^ century (Kaszab and Papp 1986). Endrődi, a scarabaeoid beetle specialist, was the principal author of over 200 scientific articles and books on beetle systematics (Kaszab and Papp 1986). In the post-World War II period, Endrődi vigorously undertook a world revision of the subfamily Dynastinae. These revisionary studies, the “Monographie der Dynastinae”, were published from 1966 through 1978 as a 22-part series. The series was later translated into English, synthesized, and published as a single volume, *The Dynastinae of the World* (Endrődi 1985a). Endrődi’s revisions (both the more detailed German-language series and the English-language book) are the basis of modern dynastine systematics research and identification.

Endrődi authored or coauthored 27 works that covered cyclocephaline scarabs from 1960 to 1985 (Endrődi 1960, 1963, 1964, 1966, 1967a–c, 1969a, b, 1970, 1971b, 1973a, b, 1975a–c, 1977a, b, 1979, 1980, 1981, 1985a, b, Howden and Endrődi 1966, Endrődi and Dechambre 1976, Dechambre and Endrődi 1983, 1984). In total, Endrődi named over 110 species and subspecies (>90 of these taxa are still valid) in cyclocephaline genera. The majority (~50% of valid taxa) of these new taxa were described in the speciose genus *Cyclocephala*. Generally, Endrődi did not describe new genera in this group (the junior synonym *Surutoides* Endrődi 1981 is the lone exception) and instead favored lumping species into relatively large genera (e.g., *Cyclocephala* *sensu* Endrődi 1966 included over 180 taxa). The tribe Cyclocephalini was covered in the first installment of the “Monographie der Dynastinae” series (Endrődi 1966). One of the earliest modern discussions on the phylogenetic position of Cyclocephalini, and Dynastinae more broadly, was included in this first installment (Endrődi 1966). Many of the most detailed portions in the German-language monograph of Cyclocephalini (Endrődi 1966) were not included in *The Dynastinae of the World* and these details warrant further discussion.

**Figure 1.**
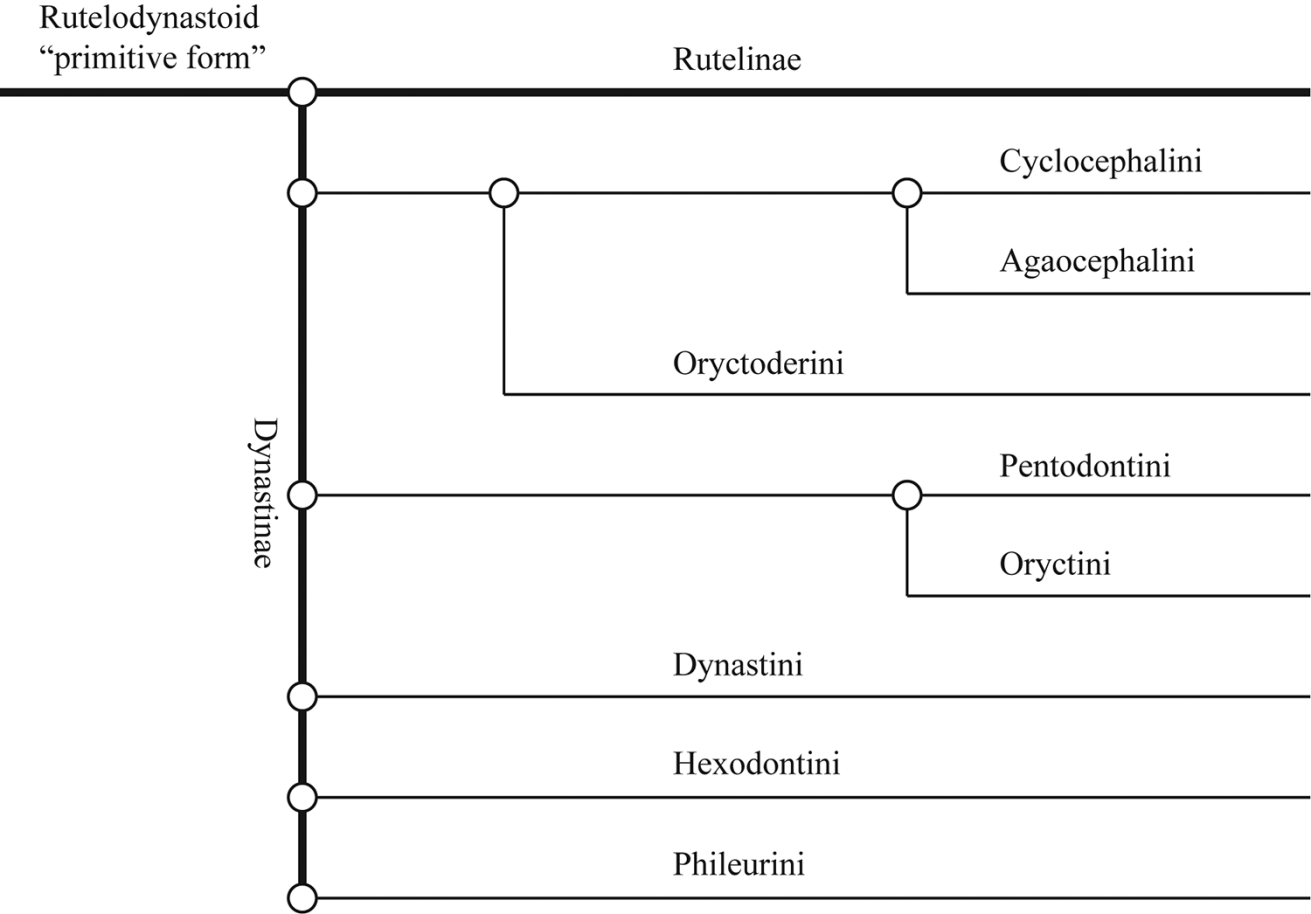
Reproduction of figure 57 from Endrődi (1966). Hypothetical relationships among the tribes of Dynastinae. “Primitive form” is translated from the German “Urform.”

Cyclocephalini was considered by Endrődi to be the most primitive tribe of Dynastinae, with many species sharing characters with Rutelinae (Endrődi 1960, 1966; Fig. 1). Endrődi’s (1966) methodology for assessing the relationships of dynastine tribes defies precise categorization within modern approaches. He attempted, with poor justification, to polarize a suite of characters into primitive and derived states within Dynastinae. Nine characters were scored as three states, which ranged from 1 (most derived) to 3 (ancestral). Character states scored as “2” indicated that both derived and ancestral states, or “partially differentiated” states, were present in each tribe (Endrődi 1966). Tribes with the highest total numerical value (numbers were summed across the matrix) were considered the most primitive overall.

This analysis suggests that Endrődi was attempting a very rudimentary cladistic approach to understanding dynastine tribal relationships. However, he did not define clear synapomorphic characters nor did he discuss homoplasy. This rudimentary approach was used only to hypothesize how “evolved” each of the eight dynastine tribes were compared to the outgroup Rutelinae. His results indicated the Cyclocephalini (score of 25) was the earliest diverging dynastine tribe, while Dynastini (score of 16) was the most derived tribe. Endrődi (1966) utilized the following characters in this analysis: 1) “body form” differentiated from Rutelinae or not; 2) presence or absence of “striking” sexual dimorphism; 3) relative length of the legs; 4) relative thickness of the protarsomeres and protarsal claws in males; 5) form of the anterior margin of the meso- and metatibia (“Hinterschienenspitze”); 6) presence or absence of stridulatory structures on the abdomen; 7) relative degree of expansion of the female elytral epipleuron; 8) presence or absence of hindwings; and 9) global distribution.

Endrődi also considered relationships among genera. A similar character polarization method was applied to cyclocephaline genera that were considered by Endrődi as valid (Endrődi 1966). Nine characters were used in this analysis: 1) clypeus short and simple to strongly differentiated; 2) lamellate club of the antennae elongated in males or not; 3) male protarsomeres thickened or not; 4) body shape vaulted, oval, or differentiated; 5) male parameres simple or differentiated; 6) prosternal peg short or elongated; 7) clypeus with or without bumps (“Höckern”); 8) elytral punctation disorganized, unistriate, or in paired striae; and 9) female elytral epipleuron strongly thickened or not. *Augoderia*, *Arriguttia*, and *Ruteloryctes* were thought to be the most “primitive” cyclocephaline genera, though Endrődi’s analysis provided only weak justification. By Endrődi’s (1966) own admission, this exercise did not yield clear results (“Aus diesen Wertzahlen ist deutlich zu erkennen, daß schon bei den Gattungen die Auswertung der primitiven und fortgeschritten Formen nur schwer vorgenommen werden kann”).

Endrődi’s (1966) diagnosis of Cyclocephalini is the most detailed published for the group, and it offers further discussion on the distribution of some character states among the tribe’s genera. Members of Cyclocephalini were diagnosed as being small- to medium-sized, primitive dynastines that share the oval and convex body shape of Rutelinae. The body shapes of the genera *Arriguttia* (anteroposteriorly compressed) and *Surutu* (dorsoventrally flattened) were considered exceptional in the tribe. Cyclocephaline mandibles were considered small (varying in width or broadness) for the subfamily and lacking teeth on the lateral, outer margin. Cephalic morphology in the tribe was notable for its lack of horns, tubercles, carinae, or sulci. The slightly raised frontoclypeal suture present in some *Ancognatha* species was a possible exception to this lack of armature on the head. These “tubercles”, however, were not considered homologous with tubercles of the head present in other dynastines (Endrődi 1966).

Cyclocephaline antennae are comprised of 8–10 antennomeres with the lamellate club always three-segmented and occasionally elongated in males (Endrődi 1966). The pronotum is convex and only dorsoventrally flattened in *Surutu*, while the scutellum is triangular. The elytra are usually 1.5 times longer than wide and are rarely shorter (e.g., *Arriguttia*). Elytral punctation is regularly spaced and paired when punctures form striae (except for *Augoderia* and *Surutu*). The females of many species have pronounced expansions of the elytral epipleural margin with or without produced lateral flanges (Endrődi 1966).

The propygidium of cyclocephalines lacks a stridulatory apparatus (Endrődi 1966). Pygidial morphology varies between the group’s genera. The pygidium is reduced in *Chalepides*, while it is a large segment in all other cyclocephaline genera. The prosternal process is relatively long and generally rounded at the apex, but has a variably present or absent button-like folding of the cuticle (Endrődi 1966). Protibial morphology in the tribe is also highly variable. The outer lateral margins of the protibia in males have 1–3 produced teeth, while most genera have no teeth on the inner lateral margin of the protibia. *Harposceles* is the lone exception for the tribe, having a small tooth on the inner margin of the protibia (Endrődi 1966).

Three genera included in Cyclocephalini *sensu* Endrődi (1966) lack thickened, foreshortened protarsomeres and enlarged (and sometimes cleft) protarsal claws in males: *Erioscelis*, *Stenocrates*, and *Coscinocephalus*. The meso- and metatarsomeres are not thickened and foreshortened in any cyclocephaline genera (though metatarsomeres are reduced in females of some *Cyclocephala* species). The apical margins of the meso- and metatibia are simple in cyclocephalines, lacking crenulated extensions (“Hinterschienenspitze fast immer gefingert”) (Endrődi 1966). Cyclocephalini *sensu* Endrődi (1966, 1985a) included 14 genera and was a strictly New World tribe, except for the Afrotropical genus *Ruteloryctes* (Table 4).

**Table 4.** The generic composition of Cyclocephalini *sensu* Endrődi (1966, 1985a).

Genera	Biogeographic Realm	Current Tribal Classification
*Ancognatha* Erichson, 1847	Neotropical and Nearctic	Dynastinae: Cyclocephalini
*Arriguttia* Martínez, 1960	Neotropical	Dynastinae: Cyclocephalini
*Aspidolea* Bates, 1888	Neotropical and Nearctic	Dynastinae: Cyclocephalini
*Augoderia* Burmeister, 1847	Neotropical	Dynastinae: Cyclocephalini
*Chalepides* Casey, 1915	Neotropical	Dynastinae: Cyclocephalini
*Coscinocephalus* Prell, 1936	Nearctic	Dynastinae: Pentodontini
*Cyclocephala* Dejean, 1821	Neotropical and Nearctic (established in Australia and Hawaii)	Dynastinae: Cyclocephalini
*Dyscinetus* Harold, 1869	Neotropical and Nearctic	Dynastinae: Cyclocephalini
*Erioscelis* Burmeister, 1847	Neotropical	Dynastinae: Cyclocephalini
*Harposceles* Burmeister, 1847	Neotropical	Dynastinae: Cyclocephalini
*Mimeoma* Casey, 1915	Neotropical	Dynastinae: Cyclocephalini
*Ruteloryctes* Arrow, 1908	Afrotropical	Dynastinae: Cyclocephalini
*Stenocrates* Burmeister, 1847	Neotropical	Dynastinae: Cyclocephalini
*Surutu* Martínez, 1955	Neotropical	Dynastinae: Cyclocephalini

Endrődi (1966) considered the criteria for defining genera like those used to define families. Within this concept, genera were phylogenetic units that needed to show several characteristics in a “constant state” to be valid (Endrődi 1966). This line of argumentation was extended into a criticism of several genera and subgenera proposed within Cyclocephalini. Casey’s generic-level hypotheses in Cyclocephalini were especially in violation of this guiding principle. Endrődi considered most of Casey’s genera as based upon only a single character and were thus invalid within his paradigm. It was also argued that Casey’s subgenera were based upon species-level characters and not applicable to higher-level classification schemes (Endrődi 1966). This led to the synonymy of nearly all of Casey’s higher-level cyclocephaline groups, some of which were tentatively adopted by other authors in the intervening period (e.g., Arrow 1937a, b, Saylor 1937, 1945, and Buchanan 1927) (Casey 1915, Endrődi 1966). The subgenus Cyclocephala (Paraclinidia) was ambiguously synonymized within *Cyclocephala*, and Endrődi (1966) commented that the group could “at most be considered a subgenus.”

An explanation of some aspects of Endrődi’s (1966) morphological approach to his revision of Cyclocephalini was provided in a section entitled “Morphologie der Tribus.” Three types of coloration schemes are found in the tribe: 1) species that are all black or dark brown, except in teneral specimens (e.g., *Surutu*, *Harposceles*, *Coscinocephalus*, *Erioscelis*, *Ruteloryctes*, *Stenocrates*, *Dyscinetus*, *Chalepides*, and occasionally other genera); 2) species that are monotoned and light in color, sometimes with darkened legs and head, and lacking dorsal maculae (e.g., *Cyclocephala* and *Aspidolea*); and 3) species with red or black dorsal maculae (e.g., *Augoderia*, *Ancognatha*, and *Cyclocephala*) (Endrődi 1966). Among species with dorsal maculae, Endrődi considered these characteristics to be highly variable within a “system” of patterning that displayed some species-level specificity. Some species vary from having elaborate dorsal maculae to being nearly free of patterning, and these species were the most challenging for precise identification (Endrődi 1966).

Short or long setae on the head and thorax were useful characters for diagnosing species. Endrődi thought that setae on the frons and anterolateral margins of the pronotum were particularly easy to observe (even when eroded) because they were erect and in obvious punctures. The shape of the clypeus, important since Burmeister (1847), was considered diagnostic in *Mimeoma*, *Ancognatha*, *Stenocrates*, and *Aspidolea* (Endrődi 1966). However, clypeal shape was considered too variable among species for diagnosing groups in other genera such as *Cyclocephala* (Endrődi 1966). Sculpturing and rugosity of the frons, interocular distance, and shape of the frontoclypeal suture were considered stable characters within species (Endrődi 1966). He noted that there is significant variation of the mouthparts (labrum, ligula, maxillae, and mandibles) among cyclocephalines and observed this variation mostly from dissected Burmeister type specimens. Due to the number of species and specimens he needed to examine, Endrődi eschewed characters that required dissection (except for male genitalia) to observe. Thus, he generally did not use mouthpart or hindwing characters in his diagnoses for genera or species. The usefulness of mouthpart and hindwing characters for circumscribing groups remains largely unevaluated in Cyclocephalini and Dynastinae.

### Late 20^th^ and early 21^st^ century French workers: Roger-Paul Dechambre, Fabien Dupuis, and Fortuné Chalumeau

Dynastine scarab enthusiast Roger-Paul Dechambre, a former curator of Coleoptera at Museum National d’Histoire Naturelle in Paris, published 21 papers or book chapters on Cyclocephalini (Dechambre 1979a–c, 1980, 1982, 1985, 1991a, b, 1992, 1995, 1997, 1999, 2000, 2006a, b, Dechambre and Duranton 2005, Dechambre and Endrődi 1983, 1984, Dechambre and Hardy 2004, Dupuis and Dechambre 1995, Ponchel and Dechambre 2003). Dechambre was a prolific describer of cyclocephaline taxa, having authored or coauthored over 80 species and subspecies in the group (only five of which are currently junior synonyms). Most of these taxa were described in *Cyclocephala* (65 species and subspecies) and *Stenocrates* Burmeister (15 species). Beyond his *Cyclocephala* expertise, he described the second species of the African genus *Ruteloryctes* (Dechambre 2006b), a species of *Chalepides* (Ponchel and Dechambre 2003), a species of *Ancognatha* (Dechambre 2000), and three species of *Aspidolea* (Dechambre 1992). Nearly all of Dechambre’s new cyclocephaline taxa are South American, which highlights the need for continued work on that fauna.

Dechambre’s treatment of cyclocephaline genera was conservative. Dechambre did not describe any new cyclocephaline genera, and he synonymized *Surutoides* with *Cyclocephala* (Dechambre 1991a). Dechambre seems to have favored treating “species groups” in lieu of upsetting the classification of *Cyclocephala*. For example, Dechambre (1997) revised the “*Cyclocephala cribrata* species group” which included the relatively large, black species of *Cyclocephala* previously included in *Mononidia* and *Surutoides*.

Fortuné Chalumeau worked on revising the West Indian scarabaeoids, especially on islands under French sovereignty. Chalumeau’s articles provided identification keys and diagnoses for *Cyclocephala*, *Chalepides*, and *Dyscinetus* species found across the Lesser Antilles (Chalumeau and Gruner 1977, Cartwright and Chalumeau 1978, Chalumeau 1982, 1983, Dutrillaux et al. 2013). Fabien Dupuis described 16 cyclocephaline species in *Aspidolea*, *Cyclocephala*, *Dyscinetus*, and *Stenocrates* (Dupuis and Dechambre 1995, Dupuis 1996, 1999, 2006, 2008, 2009, 2014, 2017, 2018). All of Dupuis cyclocephaline taxa were described from Ecuador, French Guiana, Peru, Bolivia, Venezuela, and Colombia.

### Late 20^th^ and early 21^st^ century North, Central, and South American workers: Brett Ratcliffe, Ronald Cave, Luis Joly, and Mary Liz Jameson

Brett Ratcliffe, Curator of Entomology at the University of Nebraska State Museum, greatly expanded upon Endrődi’s dynastine research in the Nearctic and Neotropical realms. Ratcliffe has authored or coauthored 39 publications that cover cyclocephaline scarabs, and many of these are monographic in scope (Ratcliffe 1977, 1978, 1981, 1985, 1986, 1989, 1991, 1992a–d, 2002a, b, 2003, 2008, 2014, 2015, Ratcliffe and Cave 2002, 2006, 2008, 2009, 2010, 2015, 2017, Ratcliffe et al. 2013, 2015, Ratcliffe and Delgado-Castillo 1990, Ratcliffe and Hoffman 2011, Ratcliffe and Morón 1997, Ratcliffe and Paulsen 2008, Figueroa and Ratcliffe 2016, Gasca-Álvarez et al. 2014, Jameson et al. 2002, 2009, Maes and Ratcliffe 1996, Maes et al. 1997, Neita-Moreno et al. 2006, 2007, Saltin and Ratcliffe 2012).

This body of research includes the description of over 60 new cyclocephaline species, only eight of which are in synonymy. These publications are mostly focused on Central or Mesoamerican taxa, but they also enhance knowledge of the poorly known South American genera *Surutu* and *Harposceles*. Ratcliffe, with collaborators Ronald Cave and Enio Cano, have systematically treated Dynastinae north of Panama, including the West Indies (Ratcliffe 2003, Ratcliffe and Cave 2006, 2015, 2017, Ratcliffe et al. 2013). These monumental works provide the most comprehensive, authoritative taxonomic treatment (synonymy and consistent species concept), identification tools, distribution data, and synthesized biological information ever produced for the subfamily in the New World. Venezuelan scarabaeologist Luis Joly, along with collaborator Hermes Escalona, advanced understanding of the group in South America, having revised *Chalepides* and the *Dyscinetus* of Venezuela (Joly and Escalona 2002, 2010). Joly has also described several new species of *Cyclocephala* from across South America and the West Indies.

Recent publications have generally been conservative regarding the generic composition of Cyclocephalini. Morón and Ratcliffe (1996) transferred the genus *Coscinocephalus* from Cyclocephalini to Pentodontini based on characters of the head, mouthparts, and parameres shared with *Orizabus* Fairmaire, 1878. The work of Mary Liz Jameson, while focused mainly on the subfamily Rutelinae, has altered the concept of Cyclocephalini (Jameson 1998, Jameson et al. 2002, Jameson and Wada 2004, 2009, Jameson and Jákl 2010, Jameson and Drumont 2013). Two genera, *Acrobolbia* Ohaus, 1912 and *Peltonotus*, previously classified in Rutelinae were transferred into Cyclocephalini based on morphological phylogenetic analyses (Jameson 1998, Jameson et al. 2002, Jameson and Wada 2004).

## Immature stages: diagnosis and identification

Research interest in cyclocephaline immature stages has recently increased, with approximately 80% of larval and pupal descriptions published after 1990 (Morelli 1991, Morelli and Alzugaray 1994, Vincini et al. 2000, Ramírez-Salinas et al. 2004, Vallejo and Morón 2008, Neita-Moreno and Morón 2008, Bran et al. 2006, Neita-Moreno et al. 2007, Vallejo and Morón 2008, Neita-Moreno and Morón 2008, Lugo-García et al. 2009, Stechauner-Rohringer and Pardo-Locarno 2010, Neita-Moreno and Yepes 2011, Albuquerque et al. 2014, Souza et al. 2014a, b, Morón et al. 2014). It is not yet possible to characterize cyclocephaline larvae or pupae at the tribal level as only 4 of 14 genera have described immatures (Table 5 and Table 6). Neita-Moreno et al. (2007) offered the most detailed tribal-level diagnosis of third-instar larvae and noted all the species known to them shared the following characters: 1) dorsal surface of last antennal segment with two sensory spots and 2) each tarsal claw with two setae. Characters of the haptomeral process (epipharynx), plegmatia (epipharynx), ocelli (head), and raster palidia (abdomen) were consistent in many, but not all, known species at the time (Neita-Moreno et al. 2007, Morón et al. 2014).

**Table 5.** Cyclocephaline species with larval descriptions or with larvae incorporated into identification keys.

**Genera**	**Species and subspecies**	**References**
*Ancognatha* Ericson, 1847	*A. manca* (LeConte)	Ritcher 1966, Ramírez-Salinas et al. 2004, Vallejo and Morón 2008, Neita-Moreno and Morón 2008
	*A. scarabaeoides* Erichson	
	*A. sellata* Arrow	
	*A. ustulata* (Burmeister)	
*Aspidolea* Bates, 1888	*A. singularis* Bates	Neita-Moreno et al. 2007
*Cyclocephala* Dejean, 1821	*C. barrerai* Martínez	Ritcher 1944, 1966, Gordon and Anderson 1981, King 1984, Morelli 1989, 1991, Morelli and Alzugaray 1994, Bran et al. 2006, Lugo-García et al. 2009, Stechauner-Rohringer and Pardo-Locarno 2010, Albuquerque et al. 2014, Souza et al. 2014a, b, Morón et al. 2014
	*C. borealis* Arrow	
	*C. celata* Dechambre	
	*C. comata* Bates	
	*C. distincta* Burmeister	
	*C. fasciolata* Bates	
	*C. fulgurata* Burmeister	
	*C. gregaria* Heyne & Taschenberg	
	*C. jalapensis* Casey	
	*C. longula* LeConte	
	*C. lunulata* Burmeister	
	*C. lurida lurida* Bland	
	*C. modesta* Burmeister (undescribed; incorporated into key by Morelli and Alzugaray [1994])	
	*C. paraguayensis paraguayensis* Arrow	
	*C. parallela* (Casey)	
	*C. pasadenae* (Casey)	
	*C. putrida* Burmeister (undescribed; incorporated into key by Morelli and Alzugaray [1994])	
	*C. signaticollis* Burmeister	
	*C. sinaloae* Howden and Endrődi	
	*C. testacea* Burmeister	
*Dyscinetus* Harold, 1869	*D. dubius* (Olivier)	Ritcher 1944, 1966, Vincini et al. 2000, Neita-Moreno and Yepes 2011
	*D. morator* (Fabricius)	
	*D. rugifrons* (Burmeister)	

**Table 6.** Cyclocephaline species with pupal descriptions.

**Genera**	**Species and subspecies**	**References**
*Aspidolea* Bates, 1888	*A. singularis* Bates	Neita-Moreno et al. 2007
*Cyclocephala* Dejean, 1821	*C. celata* Dechambre	Morelli 1989, 1991, Morelli and Alzugaray 1994, Bran et al. 2006, Stechauner-Rohringer and Pardo-Locarno 2010, Albuquerque et al. 2014, Souza et al. 2014a, b
	*C. distincta* Burmeister	
	*C. fulgurata* Burmeister	
	*C. gregaria* Heyne and Taschenberg	
	*C. paraguayensis paraguayensis* Arrow	
	*C. lunulata* Burmeister	
	*C. signaticollis* Burmeister	
	*C. testacea* Burmeister	
*Dyscinetus* Harold, 1869	*D. dubius* (Olivier)	Vincini et al. 2000, Neita-Moreno and Yepes 2011
	*D. rugifrons* (Burmeister)	

Eleven additional species of *Ancognatha*, *Cyclocephala*, and *Dyscinetus* had their larvae described since Neita-Morena et al. (2007), and these authors' diagnosis for the tribe should be reevaluated with the data presented in Table 7. The presence of two dorsal sensory spots on the terminal antennal segment is a consistent character for the tribe, except for *C. barrerai* (Morón et al. 2014) (Table 7). *Cyclocephala barrerai* has a variably present or absent third dorsal sensory spot on the terminal antennomere (Morón et al. 2014). The tarsal claws of known cyclocephaline larvae have two setae (one basal seta and one prebasal seta). *Cyclocephala celata* is the exception in the tribe, and this species has an additional prebasal seta (Souza et al. 2014b). The haptomerum of the epipharynx has a raised bilobed or entire ridge in the subfamily Dynastinae (Ritcher 1966). Among the known *Cyclocephala* and *Aspidolea* larvae (the genera with the most similar adult morphology that are comparable), the haptomerum is a tooth-like process that is divided into two lobes (or “teeth”) (Table 7). This character may prove useful for diagnosing larvae of *Cyclocephala*-like genera in the tribe if they are described in the future (e.g., *Arriguttia*, *Augoderia*, former *Mimeoma* species, and additional *Cyclocephala* species). *Ancognatha manca* has an entire haptomeral process, making it unique for the known larvae in the genus.

**Table 7.** List of proposed diagnostic characters for cyclocephaline scarab beetle larvae. Question marks indicate character states that are unreported from the literature.

Species	Haptomeral Process	Plegmatia	Ocelli	Terminal Antennal Segment with 2 Dorsal Sensory Spots	Tarsal Claw Setae	Palidia
*Ancognatha manca*	Entire	Absent	Present	Present	2 setae	Absent
*A. scarabaeoides*	Not Entire	Absent	Present	Present	2 setae	Absent
*A. sellata*	Not Entire	Absent	Present	Present	2 setae	Absent
*A. ustulata*	Not Entire	Absent	Present	Present	2 setae	Absent
*Aspidolea singularis*	Not entire	Present	Present	Present	2 setae	Absent
*Cyclocephala barrerai*	Not entire	Absent	Present	Present (variable)	2 setae	Absent
*C. borealis*	Not entire	Absent	Present	Present	2 setae	Absent
*C. celata*	Not entire	Absent	Present	Present	3 setae	Absent
*C. comata*	Not entire	Absent	Present	Present	?	Absent
*C. distincta*	Not entire	Absent	Present	Present	2 setae	Absent
*C. fasciolata*	Not entire	Absent	Present	Present	2 setae	Absent
*C. fulgurata*	Not entire	Absent	Present	Present	2 setae	Absent
*C. gregaria*	Not entire	Absent	Present	Present	2 setae	Absent
*C. jalapensis*	Not entire	Absent	Present	Present	2 setae	Absent
*C. longula*	Not entire	Absent	Present	Present	2 setae	Absent
*C. lunulata*	Not entire	Absent	Present	Present	2 setae	Absent
*C. lurida lurida*	Not entire	Absent	Present	Present	2 setae	Absent
*C. modesta*	?	?	?	?	?	Present
*C. paraguayensis paraguayensis*	Not entire	Absent	Present	Present	2 setae	Absent
*C. parallela*	Not entire	Absent	Present	Present	2 setae	Absent
*C. pasadenae*	Not entire	Absent	Present	Present	2 setae	Absent
*C. putrida*	?	?	?	?	?	Absent
*C. signaticollis*	Not entire	Absent	Present	Present	?	Absent
*C. sinaloae*	Not entire	Absent	Present	Present	2 setae	Absent
*C. testacea*	Not entire	Present	Present	Present	?	Present
*Dyscinetus dubius*	Entire	Absent	Present	Present	2 setae	Absent
*D. morator*	Entire	Absent	Present	Present	2 setae	Absent
*D. rugifrons*	Entire	Absent	Present	Present	?	Absent

Several identification keys incorporating these species have been developed. For example, Lugo-García et al. (2009, 2012) proposed an identification key for all species of phytophagous scarab larvae (including *Cyclocephala*) associated with agave and maize cultivation in Jalisco and Sinaloa, Mexico. Country specific keys for *Cyclocephala* larvae were developed for Uruguay and Colombia (Morelli and Alzugaray 1994, Bran et al. 2006, Stechauner-Rohringer and Pardo-Locarno 2010). Neita-Moreno et al. (2007) proposed a generic-level key to the tribe that included *Ancognatha*, *Aspidolea*, *Cyclocephala*, and *Dyscinetus*. Neita-Moreno and Yepes (2011) provided a key to the larvae of *Dyscinetus* and several authors have proposed keys to the known larvae of *Cyclocephala* (Souza et al. 2014a, b, Albuquerque et al. 2014). The four new larval descriptions from Morón et al. (2014) have yet to be incorporated into an identification key. Neita-Moreno and Morón (2008) provided a key to the known larvae of *Ancognatha*.

## Economic importance of larvae and adults

The habits of cyclocephaline larvae are poorly known, especially for species that are restricted to tropical forests. Species commonly encountered in temperate zones or agricultural areas are the source of the most detailed larval life history data. Cyclocephaline larvae go through three instars and pupate in soil (Ritcher 1966, Santos and Ávila 2007, Stechauner-Rohringer and Pardo-Locarno 2010, Rodrigues et al. 2010, Souza et al. 2015). Economic data from turfgrass researchers suggested that the larvae of temperate *Cyclocephala* species are rhizophagous (e.g., see Blanco-Montero and Ward 1995 and Crutchfield et al. 1995). Data from Central and South American agroecosystems indicated that *Cyclocephala* larvae are at least facultatively saprophagous, feeding on decaying plant matter and leaf litter. Information about immature stages in tropical forests is sparse, but the larvae and pupae of *Harposceles paradoxus* were found in the organic litter accumulated between leaf sheaths of the palm *Astrocaryum carnosum* F. Kahn & B. Millán (Arecaceae) (Couturier and Kahn 1992). *Cyclocephala cribrata* Burmeister larvae reportedly eat the roots of bromeliads in Brazil (Luederwaldt 1926). *Cyclocephala atricapilla* Mannerheim adults and larvae were found beneath litter near their *Annona* host plants, and the larvae were observed feeding on decaying material (Costa et al. 2017).

The economic importance of *Cyclocephala* larvae in agroecosystems is difficult to generalize as beneficial, negative, or neutral. The widespread species *C. lunulata* has been laboratory reared on decaying sugarcane and humus, indicating some saprophagous habits (Stechauner-Rohringer and Pardo-Locarno 2010). In agroecosystems, *C. lunulata* larvae have been collected in soils underneath the living and decaying roots of peanuts (*Arachis hypogaea* L.; Fabaceae), alfalfa (*Medicago sativa* L.; Fabaceae), statice (*Limonium sinuatum* [L.] Mill.), sugarcane (*Saccharum* sp.; Poaceae), maize (*Zea mays* L.; Poaceae), stevia (*Stevia rebaudiana* [Bertoni] Bertoni; Asteraceae), rice, and in pastures (Aragón and Morón 2000, Aragón et al. 2001, Bran et al. 2006, Stechauner-Rohringer and Pardo-Locarno 2010, Morón et al. 2014). However, this species is not thought to be a major damaging pest in crop systems (Aragón et al. 2001).

In contrast, *C. parallela* larvae are considered a pest in Florida “sand-muck” sugarcane production (Gordon and Anderson 1981). Sugarcane production may produce favorable soil conditions for cyclocephaline scarab beetle larvae as *Cyclocephala* and *Dyscinetus* species have been reported to be numerous in fields in Cuba, Puerto Rico, Nicaragua, Colombia, and Guyana (Box 1925, Stahl and Scaramuzza 1929, Squire 1932, 1933, Maes and Tellez 1988, Posada Ochoa 1989). *Cyclocephala testacea* can reach densities of 160 larvae/m^2^ of soil in Uruguayan pastures (Morelli and Alzugaray 1994). At these densities, the larvae form noticeable mounds, denude soil, and possibly contribute to weediness of fields (Morelli and Alzugaray 1994).

The larvae of several *Ancognatha* species are pests in barley, (*Hordeum vulgare* L.; Poaceae), rye (*Secale cereale* L.; Poaceae), maize, oats (*Avena sativa* L; Poaceae), onions (*Allium cepa* L.; Amaryllidaceae), carnations (*Dianthus* spp.; Coryphyllaceae), strawberries (*Fragaria* spp.; Rosaceae), and tamarillo (*Solanum betaceum* Cav.; Solanaceae). (Posada Ochoa 1989, Ruíz and Pumalpa 1990). The association of *Ancognatha* larvae with cultivated commodity flowers in Colombia is a challenge for USDA APHIS inspectors. For example, *Ancognatha* adults of several species from Colombia (presumably emerged from soil) are routinely intercepted with flower imports of *Gypsophila* (Coryphyllaceae), *Dianthus*, *Alstroemeria* (Alstroemeriaceae), and *Limonium* (Coryphyllaceae) (pers. comm. with Charles Brodel, May 2017). As an occasional and sporadic pest, *C. variabilis* Burmeister can affect tea (*Camellia sinesnsis* (L.) Kuntze; Theaceae) cultivation in Brazil (Monte 1933). *Cyclocephala signaticollis* damages potato (*Solanum tuberosum* L.; Solanaceae) tubers and several garden or field crops in Argentina (Remedi de Gavotto 1964, San Martin 394, Berón and Diaz 2005). Similar damage to potato production by larvae has been documented for other *Cyclocephala* and *Ancognatha* species in Bolivia and Colombia (Squire 1972, Posada Ochoa 1989, Montoya et al. 1994).

Adult cyclocephaline scarab beetles are relatively less important as pests of agroecosystems. However, some species have been recorded to chew on the foliage, consume pollen, seeds, and fruits. The conditions in which adults of these species become pests in these systems is not clear and well documented examples are rare. Colombian *Cyclocephala ruficollis* Burmeister were observed to chew on the foliage of sesame (*Sesamum indicum* L.; Pedaliaceae), cotton (*Gossypium* spp.; Malvaceae), maize, banana shoots (*Musa* spp.; Musaceae), and sunflowers (*Helianthus annuus* L; Asteraceae) (Posada Ochoa 1989). *Cyclocephala ovulum* Burmeister has also been reported to attack seeds of sunflower in Argentina (Hayward 1946). The foliage of common beans (*Phaseolus vulgaris* L.; Fabaceae) and African oil palm (*Elaeis guineensis* Jacq.; Arecaceae) are chewed by *C. amazona* (reported as *C. signata*) in Colombia (Posada Ochoa 1989). An unidentified *Cyclocephala* chews foliage of cassava (*Manihot esculenta* Crantz; Euphorbiaceae) (Posada Ochoa 1989). In addition to sunflowers, *C. ruficollis* and *C. amazona* reportedly feed on the flowers of *Citrus* (Rutaceae), various ornamental plants, maize, and *C. ruficollis* will feed on the pollen of sorghum (*Sorghum* sp.; Poaceae) in Colombia (Posada Ochoa 1989). Similar flower feeding on *Citrus* has also been reported for *C. melanocephala* in Brazil (Remillet 1988). At least two *Cyclocephala* species will eat fruit of cultivated rose apples (*Syzygium jambos* (L.) Alston; Myrtaceae), custard apples (*Annona* spp.; Annonaceae), and guava (*Psidium guajava* L.; Myrtaceae) (Posada Ochoa 1989). A *Stenocrates* sp. may also attack foliage of maize in Colombia and sugarcane in Brazil (Lima 1953, Posada Ochoa 1989).

The role of *Dyscinetus* species in agroecosystems is not clear. It is possible that some reports of damage to crops by *Dyscinetus* are complicated by misidentifications of the similar looking genus *Euetheola* Bates (Scarabaeidae: Dynastinae: Pentodontini) (Phillips and Fox 1924). In some cases, *Dyscinetus* species have been reported in association with crop systems but are considered non-damaging saprophages. For example, the larvae of *Dyscinetus* sp. in Puerto Rico can be found in association with rotting stumps of sugarcane but they apparently do not attack the roots of living plants (Smyth 1916). In contrast, *D. rugifrons* is considered a pest of cultivated sugar cane in Argentina where the larvae burrow into internodes and buds (Costilla 1991). Adult *D. rugifrons* attack the shoots, but this is rare (Costilla 1991). In another case of conflicting information, Phillips and Fox (1924) reported that *D. morator* would not attack maize in their experiments. However, adults of this species will attack young maize shoots in North Carolina in fields with wet, high organic matter soil (Anonymous 1980).

*Dyscinetus gagates* Burmeister can be a silvicultural pest in Argentina during years when populations of the beetles are high. *Dyscinetus rugifrons* adults attack the stems and roots of young cultivated *Populus* hybrids (Salicaceae) (Moore 1958) and *Eucalyptus* (Myrtaceae) (Bosq 1945), killing the plants. In Florida, *D. morator* adults attack carrots (Apiaceae), radishes (Brassicaceae) (Foster et al. 1986), and the bulbs, buds, and petioles of cultivated *Caladium* (Araceae) (Anonymous 1971, Price and Kring 1991). Larvae of this species also damage Pangola-grass pastures in Florida when at high densities (Anonymous 1956). In Maryland, *D. morator* larvae can damage the roots of azaleas (*Rhododendron* spp.; Ericaceae) (Staines 1990). *Dyscinetus morator* larvae can damage the fine root tips of cranberry (*Vaccinium* sp.; Ericaceae) in bog cropping systems, though they are considered minor pests (Scammell 1917).

## Natural enemies: predation, parasites, and infections

### Vertebrate predation

Several species of wetland birds, reptiles, and amphibians prey on *Chalepides*, *Cyclocephala*, and *Dyscinetus* species in mucky habitats. White-faced ibis (*Plegadis chihi* (Vieillot)), white ibis (*Eudocimus albus* (Linnaeus)), and scarlet ibis (*E. ruber* (Linnaeus)) eat adult *Dyscinetus* and *Chalepides* in Argentina and Venezuela (Aguilera et al. 1993, Soave et al. 2006.). Common terns (*Sterna hirundo* Linnaeus), white-browed blackbird (*Sturnella superciliaris* (Bonaparte)), yellow-winged blackbird (*Agelaius thilius* (Molina)), Olrog’s gull (*Larus atlanticus* Olrog), and brown-hooded gull (*L. maculipennis* Lichtenstein) eat *Dyscinetus* spp. and *C. signaticollis* in Argentinian marshes, grasslands, lagoons, and riparian areas (Darrieu et al. 2001, Mauco and Favero 2004, Camperi et al. 2004, Ghys and Favero 2004, Berón and Favero 2010). Clapper rails (*Rallus crepitans* Gmelin) hunt *D. morator* in Louisiana marshes (Roth et al. 1972). Wattled Jacana (*Jacana jacana* (Linnaeus)) have been observed to catch and eat *Cyclocephala* species associated with Amazonian water lilies (Prance and Arias 1975). Lizards and birds will quickly eat *Cyclocephala* if they are knocked out of *Cyclanthus* spathes during the day (Beach 1982).

Juvenile brown caimans (*Caiman crocodilus fuscus* (Cope)) in Costa Rica feed primarily on insects, especially *Dyscinetus* (Allsteadt and Vaughan-Dickhaut 1994). The invasive cane toad (*Rhinella marina* (Linnaeus)) eats *C. barbatus* in Puerto Rico (Wolcott 1937). In the American southwest, Couch’s spadefoot toad (*Scaphiopus couchii* Baird) will readily eat *A. manca* and *Cyclocephala* species (Dimmitt and Ruibal 1980). Mammal predation on cyclocephalines has rarely been documented, but it is suspected that fossorial mammals, such as armadillos, would consume larvae (Tashiro 1987). Mountain coati, *Nasuella olivacea* (Gray), dig up and eat *A. scarabaeoides* larvae in the Eastern and Central Colombian Cordilleras (Apolinar Maria 1946). Several species of bat are known to eat *Cyclocephala* seasonally or opportunistically (Goldman and Henson 1977, Johnston and Fenton 2001, Lenoble et al. 2014).

### Invertebrate predators and parasitoids

Cyclocephaline scarab beetle larvae are subject to parasitism by ecto- and endoparasitoid flies and wasps. The fly *Mallophora ruficauda* Wiedemann (Diptera: Asilidae) is a koinobiont parasitoid of *C. signaticollis* (Barrantes and Castelo 2014). *Mallophora ruficauda* can also attack *C. putrida* and *C. modesta*, but the fly does not complete its development on these hosts or the adult flies are stunted and deformed (Barrantes and Castelo 2014). Two other asilid flies, *M. sylverii* Macquart and *Diogmites vulgaris* Carrera, parasitize *Dyscinetus rugifrons* in Brazil (Dennis and Knutson 1988). *Dyscinetus* species are parasitized by *Tiphia parallela* Smith (Hymenoptera: Tiphiidae) in Guyana (Box 1925). *Tiphia pygidialis* Allen parasitizes *C. borealis*, *C. lurida lurida*, and *C. pasadenae* (Rogers and Potter 2004). *Cyclocephala pasadenae* was demonstrated to be toxic to spiders of several families when eaten, though the mechanism of this toxicity remains unexplained (Cokendolpher 1993). Ants can be significant egg and larval predators of *C. lurida lurida* in turfgrass (Zenger and Gibb 2001). The parasitoid larvae of *Plega banksi* Rehn (Neuroptera: Mantispidae: Symphrasinae) attack *Cyclocephala* pupae in Arizona (Werner and Butler 1965).

Cyclocephalines, like many relatively large beetles, are hosts of phoretic mites. Acarid and macrochelid mites have been reported from *Cyclocephala* (Goldwasser 1987, Crocker et al. 1992). Phoretic macrochelid mites on *Cyclocephala* are common in aroid inflorescences visited by the beetles, and the mites appear to feed on floral exudates (Goldwasser 1987). The mesostigmatid *Dyscinetonyssus hystricosus* Moss and Funk is hypothesized to be a parasite of *D. morator* (Moss and Funk 1965). This conclusion was based on morphological features of the mites consistent with parasitic habits and the observation that all life-stages and sexes of the mites are present on *D. morator* (Moss and Funk 1965).

### Entomopathogenic nematodes and worms

Entomopathogenic nematodes are remarkable for their ability to attack and kill numerous insect pests. Their flexibility of use, combinability with other chemical and biological controls, and safety has led to their use in IPM strategies for control of *C. borealis*, *C. pasadenae*, *C. lurida lurida*, and *C. hirta* grubs (Kaya et al. 1995, Koppenhöffer and Kaya 1997, 1998, Converse and Grewal 1998, Koppenhöffer and Fuzy 2003, Koppenhöffer et al. 1999, 2002, 2004). Many species and strains of *Steinernema* Travassos (Nematoda: Steinernematidae) and *Heterorhabditis* Poinar (Heterorhabditidae) infect these *Cyclocephala* species, though *C. pasadenae* appears to have the most natural resistance to nematode infection among examined North American *Cyclocephala* (Koppenhöffer and Kaya 1996, Koppenhöffer et al. 2004).

Nematode infections of South American cyclocephalines have received some attention. The Argentinian pest grub *C. signaticollis* is naturally infected by two rhabditid and two thelastomatid nematodes (Reboredo and Camino 2000, Camino and Reboredo 2005, Camino and Achinelly 2012). *Cyclocephala modesta* hosts a thelastomatid parasitic nematode in its alimentary canal (Achinelly and Camino 2008). *Ancognatha scarabaeoides*, a major grub pest in Colombia, can be readily infected by *Steinernema* nematodes (Lucero Malfa et al. 2006). *Dyscinetus morator* can be an intermediate host of the swine parasite, thick stomach worm (*Ascarops strongylina* [Rudolphi]; Nematoda: Spirocercidae) (Fincher et al. 1969). Beyond nematodes, information regarding the infection of cyclocephalines by other worms is lacking. The only known example is that of *D. gagates* adults, which are suitable intermediate hosts of the rat tapeworm (*Hymenolepis diminuta* [Rudolphi]; Cestoda: Hymenolepididae) under laboratory conditions (Bacigalupo 1939).

### Entomopathogenic bacteria and fungi

Bacterial and fungal pathogens have proven useful for IPM of injurious scarab grubs, especially Japanese beetle (*Popillia japonica* Newman). Several of the most important pathogens for *P. japonica* control have been explored for use on *Cyclocephala* species. The fungal parasites *Beauveria bassiana* (Bals.-Criv.) Vuill and *Metarhizium anisopliae* (Metchnikoff) Sorokin (both Sordariomycetes: Hypocreales) have been evaluated for pathogenicity and virulence in *C. signaticollis*, *C. borealis*, and *C. lurida lurida* (Berón and Diaz 2005, Redmond and Potter 2010). Experiments demonstrated that one Brazilian strain of *B. bassiana* caused significant mortality against *C. signaticollis*, while native strains of *M. anisopliae* were not pathogenic in this species (Berón and Diaz 2005). This relatively low mortality caused by *B. bassiana* and *M. anisopliae* was also observed in *C. lurida lurida*, but both fungal pathogens display synergism with entomopathogenic nematodes (Wu et al. 2014). *Cyclocephala borealis* and *C. lurida lurida* larvae surveyed from Kentucky golf courses also showed low infection rates by *M. anisopliae* (Redmond and Potter 2010). *Cyclocephala parallela* can also be naturally infected by *M. anisopliae* in sugarcane fields (Boucias et al. 1986). *Metarhizium anisopliae* – based control measures of *A. scarabaeoides* may have promise in Colombia, as at least one identified strain causes high mortality in this species (Marino et al. 2004).

Milky disease, caused by the bacterium *Paenibacillus popilliae* Dutky (Bacillales: Paenibacillaceae), is the only registered biological control specifically for *P. japonica* (Koppenhöfer et al. 2000). Infections of the disease are chronic in populations, but infection rates grow slowly (Klein 1992). Thus, milky disease is effective for inoculative, long-term treatments rather than as an emergency control measure (Klein 1992). Several *Cyclocephala* species can be infected by *P. popilliae*. *Cyclocephala parallela* larvae infected by *P. popilliae* show significantly higher mortality than healthy larvae (Boucias et al. 1986, Cherry and Boucias 1989, Cherry and Klein 1997).

*Bacillus thuringiensis* Berliner (Bt) is the most important bacterial biological control agent of insects, but there is a lack of information about infectivity in cyclocephalines. What is known about Bt in *Cyclocephala* suggests that infections enhance other biological control methods. Like fungal infections, bacterial infections by B. t. subspecies japonensis Buiui and *P. popilliae* cause additive or synergistic mortality with entomopathogenic nematodes in *C. hirta* and *C. pasadenae* (Thurston et al. 1993, 1994, Koppenhöfer and Kaya 1997, Koppenhöfer et al. 1999). Bt isolated from *C. signaticollis* in Argentina caused 100% mortality in inoculated larvae (Consolo et al. 2010).

## Human use as food

Beetles are the most commonly consumed insects by humans (van Huis et al. 2013). Many phytophagous scarab larvae reach large sizes by the 3^rd^ instar and can be found in abundance, making these beetles a valuable food resource. Data about the consumption of cyclocephaline scarab beetles is lacking, but there are a few well documented examples. The Lacandon people of Chiapas eat larval, pupal, and adult *C. fasciolata* (Ramos-Elorduy and Pino Moreno 2002). Additionally, *C. capitata* Höhne is eaten in southwestern Mexico and *C. guttata* Bates larvae and adults are eaten in Veracruz (Ramos-Elorduy and Pino Moreno 2004). Ecuadorians eat the larvae of *Ancognatha castanea* Erichson, *A. jamesoni* Murray, and *A. vulgaris* Arrow (Onore 1997, 2005). Similarly, the larvae of an unidentified *Ancognatha* species may be regularly fried and eaten in Cauca, Colombia (DeFoliart 2012). Among American Indians in the western US, the Mono Lake and Owens Valley Paiute would roast and eat adult *Phyllophaga* sp. (Scarabaeidae: Melolonthinae) (Sutton 1988). These groups may have also eaten common *Cyclocephala* spp., but this is unconfirmed (Sutton 1988). In Thailand, Karen-speaking people from the Tak province fry and eat adult *Peltonotus nasutus* Arrow that they collect from the inflorescences of *Amorphophallus paeoniifolius* (Araceae) (Danell 2010).

## Cyclocephalines as floral visitors

### Scope of the Mutualism

Based on the most specific available data, about 97 cyclocephaline scarab beetle species have been reported from the flowers of at least 58 plant genera representing 17 families and 15 orders (Moore and Jameson 2013), though new data are being published often. The preponderance of data suggests that tropical cyclocephaline species are involved in a pollination mutualism with species in the early-diverging angiosperm families Nymphaeaceae, Annonaceae, Magnoliaceae, Araceae, Cyclanthaceae, and Arecaceae (Moore and Jameson 2013). More sporadic data suggests that cyclocephaline floral visitation of more derived angiosperm groups is opportunistic and not adequately explained. However, based on the observations of Prance (1976), Cyclocephala species may be unrecognized pollinators of some Neotropical genera of the Brazil nut family (Lecythidaceae).

The mutualism between cyclocephaline scarab beetles and these early-diverging angiosperms has resulted in a cantharophilous floral syndrome in these groups. This floral syndrome is the result of the convergent evolution of several floral traits that accommodate “mess-and-spoil” beetle pollination (Faegri and van der Pijl 1979). Among the families Nymphaeaceae, Annonaceae, Magnoliaceae, Araceae, Cyclanthaceae, and Arecaceae these convergent floral traits include: 1) bisexuality of flowers or inflorescences; 2) protogyny; 3) nocturnal flower activity; 4) relatively large flowers or inflorescences that provide a “pollination chamber” and are sturdy enough to withstand beetle damage; 5) thermogenesis during anthesis; 6) production of excess pollen, floral exudates, or sterile floral parts as a food reward; 7) coordination of timing between beetle behavior, thermogenesis, and floral sexual stages; 8) large pollen grains; 9) sticky floral exudates; 10) strong floral scents and; 11) pale colored flowers or inflorescences (Bawa and Beach 1981, Bernhardt 2000, Silberbauer-Gottsberger et al. 2001, Davis et al. 2008, Thien et al. 2009, Gibernau et al. 2010). Excellent observational and experimental evidence indicates that cyclocephaline scarab beetles are primary or secondary pollinators of these plant groups (Cramer et al. 1975, Beach 1982, 1984, Young 1986, 1988a, b, Gottsberger 1989, Dieringer et al. 1999, Hirthe and Porembski 2003, Maia et al. 2012). Cyclocephalines are offered rewards for their pollination of these families. These rewards include access to aggregation and mating sites, food, and metabolic boosts associated with floral thermogenicity.

Facultative endothermy (sustained increase in thoracic muscle temperature) during rest, terrestrial activity, and preparation for flight has been documented in Coleoptera and Scarabaeidae more narrowly, including *Cyclocephala* species (Bartholomew and Casey 1977a, b). Among some examined dung beetles, changes in thermoregulation and behavior are associated with high levels of intra- and interspecific competition for rapidly depleting dung resources (Heinrich and Bartholomew 1979, Ybarrondo and Heinrich 1996). *Cyclocephala colasi* Endrődi experience sporadic bouts of endothermy during the early evening when these beetles fly between inflorescences (Seymour et al. 2009). These bouts of endothermy are more intense at lower ambient temperatures and continue throughout the night, when they may be associated with feeding, mating, or escape behaviors (Seymour et al. 2009). The host plant, *Philodendron solimoesense* A.C.Sm. (Araceae), continues thermogenesis even after floral scent compounds have been volatilized (Seymour et al. 2003). This suggests that the increased temperature of the inflorescences serves as a thermal reward to the beetles, lowering the amount of energy spent achieving sporadic endothermy (Seymour et al. 2003, 2009). Thermal rewards of this nature are predicted to be more important in montane forest habitats with much lower average ambient temperatures than lowland rainforests (Seymour et al. 2009).

Cyclocephaline scarab beetles have been observed to mate within the inflorescences or flowers of many families: 1) Nymphaeaceae (Prance and Arias 1975; Hirthe and Porembski 2003); 2) Annonaceae (Gottsberger 1990, Murray 1993, Costa et al. 2017); 3) Magnoliaceae (Gibbs et al. 1977, Dieringer and Espinosa 1994, Dieringer et al. 1999); 4) Cyclanthaceae (Beach 1982); 5) Araceae (Young 1986, 1988a, b, Maia and Schlindwein 2006, Grimm 2009, Seymour et al. 2009, Moore 2012); 5) Arecaceae (Beach 1984, Rickson et al. 1990, Voeks 2002); 6) Solanaceae (Ratcliffe and Cave 2017); and possibly 7) Cactaceae (B. Schlumpberger *in litt*. 2011). Large, chamber-like flowers also serve to protect the beetles from predation (Prance and Arias 1975, Beach 1982).

Floral food rewards for these scarab beetles are diverse and include sterile staminate or staminode tissue (Prance 1976, 1980, Young 1986, Maia et al. 2010, Maldonado et al. 2015), carpellary appendages (Prance and Arias 1975, Hirthe and Porembski 2003), stamens (Dieringer and Espinosa 1994, Hirthe and Porembski 2003, Costa et al. 2017), petal tissue (Gibbs et al. 1977, Gottsberger 1989, Dieringer and Espinosa 1994, Dieringer et al. 1999, Voeks 1992), specialized adaxial food tissue of bracts (Beach 1982), and pollen (Rickson et al. 1990). *Cyclocephala amazona* was observed consuming epidermal trichomes from the stalk of *Bactris gasipaes* Kunth (Arecaceae) inflorescences before feeding on pollen (Rickson et al. 1990). These trichomes are hypothesized to serve as non-nutritional gastroliths that aid in the piercing of pollen grains in the beetles’ gut (Rickson et al. 1990). Some *Cyclocephala* species may be destructively florivorous and detrimental to the reproductive success of the plants they visit. For example, *Cyclocephala* species are known to destructively feed on flowers of some crop plants (Remillet 1988, Posada Ochoa 1989) and the cactus species *Echinopsis ancistrophora* Speg. (Schlumpberger et al. 2009) and *Opuntia monocantha* Haw (Lenzi and Orth 2011).

### Attraction to flowers and inflorescences

Cyclocephaline attraction to their floral hosts is hypothesized to be driven by both long-distance chemical cues and short-distance visual stimuli. In the case of *Philodendron bipinnatifidum* Schott ex Endl. (Araceae), *Erioscelis emarginata* (Mannerheim) will not land on inflorescences covered in black cloth (obscuring visual stimuli associated with the scent releasing plant) (Gottsberger and Silberbauer-Gottsberger 1991). Furthermore, experiments demonstrated that these beetles were differentially attracted to *P. bipinnatifidum* spathes covered in yellow paper, indicating that contrasting colors play a role in close range attraction (Gottsberger and Silberbauer-Gottsberger 1991). Slight differences in spathe color and scent have also been hypothesized to influence the community of *Cyclocephala* spp. visiting *Dieffenbachia* spp. inflorescences in Costa Rica and Panama (Beath 1999). The white flowers of *Victoria amazonica* (Poepp.) J.C. Sowerby (Nymphaeaceae) have been hypothesized to aid in the attraction of cyclocephalines, along with their heavy floral scent (Prance and Arias 1975). Contrasting colors have also been suggested to play a role in the attraction of *Cyclocephala* species to *Cyclanthus* (Beach 1982).

The chemical composition of the floral scents attractive to cyclocephalines has received some research attention. These heavy scents are generally only volatile at elevated temperatures during floral thermogenesis. For example, protogynous *P. bipinnatifidum* inflorescences can reach an astonishing 46˚C during the female phase of anthesis (Gottsberger and Silberbauer-Gottsberger 1991). Research on these floral scents reveals that while they are complex chemical mixtures, a single dominant scent compound is sufficient for cyclocephaline attraction. In Brazil, the nitrogen and sulfur containing compound 4-methyl-5-vinylthiazole is the dominant floral scent constituent in four *Annona* spp. (Annonaceae) and *Caladium bicolor* (Aiton) Vent. (Araceae) pollinated by *Cyclocephala* species (Maia et al. 2012). Scent trap experiments confirmed that this compound alone was sufficient to attract these beetles (Maia et al. 2012).

Dötterl et al. (2012) identified three main compounds present in the *P. bipinnatifidum* floral scent that are attractive to *E. emarginata*. The dominant compound alone, 4-vinylanisole (also called 4-methoxystyrene), was sufficient to attract *E. emarginata* and various mixtures of the three scents also served to attract the beetles (Dötterl et al. 2012). A mixture of dihydro-β-ionone and methyl jasmonate was synergistically attractive to *E. emarginata*, which pollinates *Philodendron adamantium* Mart. ex Schott (Araceae) (Pereira et al. 2014). Among *Nymphaea* spp. (Nymphaeaceae) pollinated by *Cyclocephala*, floral scents are dominated by aromatic ethers and aliphatic esters (Maia et al. 2014). 4-vinylanisole is also present in *Nymphaea* species pollinated by *Cyclocephala*, suggesting that some *Nymphaea* spp. and *P. bipinnatifidum* may have converged on a similar floral scent for attracting these beetles. The ester methyl-2-methylbutanoate is the dominant floral scent compound in *Magnolia ovata* (A.St.-Hil.) Spreng. (Magnoliaceae) and is sufficient to attract *C. literata* Burmeister (Gottsberger et al. 2012). (*S*)-2-hydroxy-5-methyl-3-hexanone is one of the dominant compounds in the floral scent of *Taccarum ulei* Engl. & K.Krause and is sufficient to attract its *Cyclocephala* pollinators (Maia et al. 2013).

The mechanisms of attraction of cyclocephalines to other flower groups is poorly understood. The phytelephantoid palms (Arecaceae) *Phytelephas aequatorialis* Spruce, *P. macrocarpa* Ruiz & Pav., *P. seemannii* O.F. Cook, and *Aphandra natalia* (Balslev & A.J. Hend.) Barfod, all visited by *Cyclocephala*, have floral scents that are dominated by 4-methylanisole and 2-methoxy-3-sec-butyl pyrazine (Ervik et al. 1999). The presence of anisoles in the floral scents of phytelephantoid palms, Nymphaeaceae, and Araceae suggests that this class of compounds may have convergently evolved in these groups for attraction of cyclocephalines. *Cyclanthus bipartitus* Poit., visited by several *Cyclocephala* species, has a floral scent dominated by a unique compound called (*E*)-cyclanthone (Schultz et al. 1999). Heavy floral scents are likely to play a role in cyclocephaline attraction in every case. For example, *C. melanocephala* has been collected in the flowers of *Datura* and related genera (Solanaceae) from across its range (Moore and Jameson 2013). The dominant floral scent compounds found in these flowers are very different from those in early diverging angiosperms described above, and are comprised mostly of terpenes, terpenoids, and aromatic alcohols (Raguso et al. 2003).

### Redundancy of pollinating cyclocephalines

Some authors have speculated that floral scent compounds are serving as surrogate sex pheromones for cyclocephalines (Schatz 1990, Dieringer et al. 1999). No specific *Cyclocephala*-derived sex pheromones have been chemically identified (Leal 1996), though some North American *Cyclocephala* species appear to use volatile pheromones. For example, *C. lurida* and *C. borealis* females use pheromones to attract males, and these pheromones are cross-attractive to males of both species (Potter 1980). Further experiments demonstrated that *C. lurida* larvae produce a similar male-attracting compound that elicits attempted mating (Haynes et al. 1992). These pheromones are present in all three instars and pupae (Haynes and Potter 1995). Cross-attractiveness of *C. lurida* pheromone extracts are limited to *C. borealis*, as *C. pasadenae* and *C. longula* are not attracted to these scents (Bauernfeind et al. 1999).

In cases of cross-attractive pheromones, it can be predicted that some other mechanism (temporal or behavioral) maintains species boundaries. For sympatric *C. lurida* and *C. borealis* in Kentucky, differences in peak flight time and mating periods throughout the night serve to temporally isolate these species (Potter 1980). If attractive floral scents are serving as sex pheromones for tropical cyclocephalines, then the mechanisms isolating species remain unexplained. Only one case of interspecific copulation has been documented for cyclocephalines. The South American species *C. putrida* was observed mating at light traps, and several male *C. putrida* copulated with females of a *Tomarus* sp. (Dynastinae: Pentodontini) (Bosq 1936). Because these tropical cyclocephalines often mate within their host inflorescences, it is unclear how sexual isolation is maintained when congenerics are present. Diagnostic secondary sexual characters of the elytral epipleuron in females and protarsal and paramere morphology in males may be involved in the sexual isolation of cyclocephaline species (Moore 2012).

Many different cyclocephaline species can be found associated with a floral host at a specific time or throughout a season. There is little evidence for monophagy in the group, and available data indicate that tropical cyclocephalines are predominantly oligophagous or polyphagous floral feeders (Moore and Jameson 2013). For example, *C. bipartitus* inflorescences can contain up to three *Cyclocephala* species at one time (Beach 1982). Parsing out how redundant cyclocephalines are in their pollinator functions has been assessed in a few cases. Detailed studies on *Dieffenbachia* Schott (Araceae) indicate that among a group of cyclocephaline floral visitors, some species are relatively more effective pollinators (Young 1988a). Seasonal abundance of cyclocephalines at a specific locality, along with floral phenology, may also determine which species are primary or secondary pollinators (Maia et al. 2010, Costa et al. 2017).

## Evolution and fossil record

### Fossil cyclocephalines

The only known cyclocephaline fossil is from the extant South American species *C. signaticollis*. A fossilized elytron and pronotum of an unsexed *C. signaticollis* individual were discovered in Buenos Aires Province, Argentina (Ramírez and Alonso 2016). The fossil is from the Late Pleistocene (Tarantian Stage) and the sediments containing the fossil dated between 12,100 ± 100 BP and 13,400 ± 200 BP (Ramírez and Alonso 2016). Neoichnological experiments demonstrated that *C. borealis* and *C. lurida lurida* larvae create diagnostic backfilled meniscate burrows and ellipsoidal chambers as they burrow through soil, while adults create poorly organized backfilled burrows (Counts and Hasiotis 2009). The diagnostic features of these burrows may allow for the future detection of cyclocephaline scarab beetle ichnofossils.

### Cyclocephaline Phylogeny

Very little is known about the phylogeny of Dynastinae, and the monophyly of its tribes is in doubt. The lack of phylogenetic framework for the subfamily has limited the ability to hypothesize sister relationships among tribes and reconstruct the evolution of ecological (e.g., the floral feeding syndromes in Cyclocephalini) and morphological (e.g., such as thoracic and cephalic armature in Oryctini and Dynastini) traits. Indeed, the most meaningful comparison of characters for Dynastinae in the literature has centered around the subfamily’s relationship to Rutelinae, especially among cyclocephalines (Jameson 1998, Jameson et al. 2002, Jameson and Wada 2004). Several studies have begun to address this gap in knowledge.

The morphological phylogenetic analysis (128 characters) of Rutelina (Rutelinae: Rutelini) (Jameson 1998) was the first empirical study to suggest that the monobasic ruteline tribal- and subtribal-groups Peltonotini and Acrobolbiina were more closely related to Cyclocephalini than Rutelini. This analysis, however, did not include enough exemplar taxa from Dynastinae to conclude anything about tribal relationships in the subfamily. Schiestl and Dötterl (2012) used an analysis of 18S sequence data to examine the evolution of olfactory preferences in scarabaeoids. This analysis suggested a sister relationship between Dynastinae and Rutelinae, but it did not resolve intrasubfamilial relationships of the included genera nor did it report statistical support for recovered nodes (Schiestl and Dötterl 2012). A *Cyclocephala* exemplar species was included in this analysis, and this species fell within the dynastine clade (Schiestl and Dötterl 2012). Rowland and Miller (2012) performed a four-gene phylogenetic analysis of Dynastini (Dynastinae) that included one *Cyclocephala* exemplar. This analysis was useful for recovering subtribal relationships within Dynastini, but the relationship of Dynastini to Cyclocephalini (*Cyclocephala*) and Pentodontini (*Orizabus*) was unresolved (Rowland and Miller 2012, see also Jin et al. 2016).

**Figure 2.**
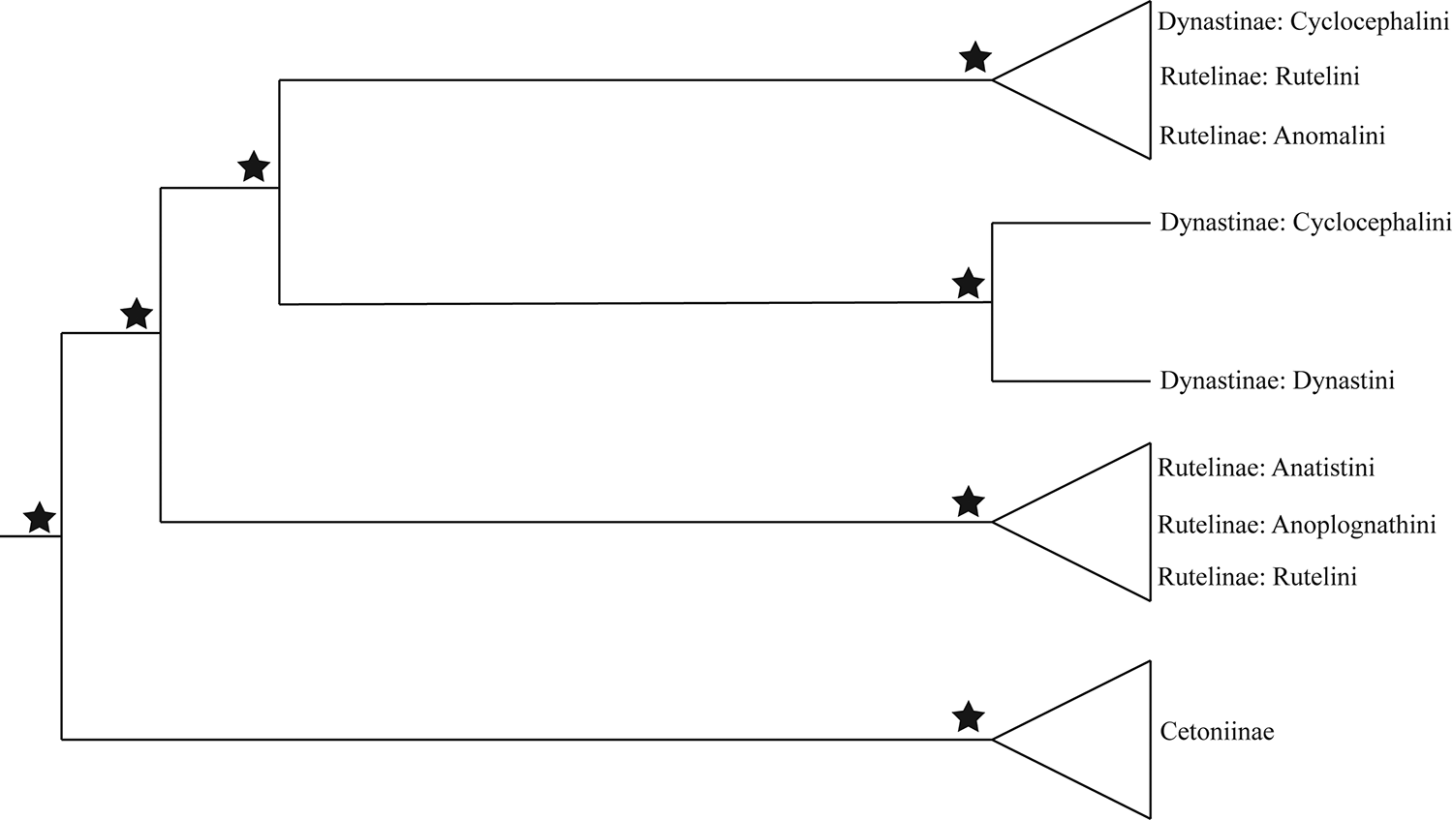
Summary of the hypothetical relationships of Dynastinae and Rutelinae tribes from McKenna et al. (2014). Stars indicate nodes with >75% bootstrap support. All but one of the starred nodes (Cyclocephalini + Rutelini + Anomalini) also had >0.95 posterior probability.

The most informative molecular phylogenetic analyses of phytophagous scarabs to date were conducted by McKenna et al. (2014) and Gunter et al. (2016). Both studies represent huge leaps forward in our understanding of subfamilial relationships in Scarabaeidae due to their resolution, statistical support, and taxa sampling. Despite their strengths, these studies are difficult to compare because of differences in gene selection and small (but significant for interpretation) differences in taxa sampling. McKenna et al. (2014) utilized 28S and CAD to phylogenetically analyze staphyliniform beetle (Histeroidea, Hydrophiloidea, and Staphylinoidea) relationships while using Scarabaeiformia as an outgroup. The most derived group of Scarabaeidae recovered from this analysis was a clade that included Cetoniinae + (Dynastinae and Rutelinae) (McKenna et al. 2014) (Fig. 2). Rutelinae was recovered as polyphyletic (McKenna et al. 2014) (Fig. 2). Three orthochilous (labrum vertically produced from clypeus and fused to clypeus) and three homalochilous (labrum horizontally produced relative to the clypeus and separated from the clypeus by a suture) rutelines from four total tribes were included in the analysis (McKenna et al. 2014). The included orthochilous rutelines (Anoplognathini and Anatistini) were recovered in the same clade, but the group was not monophyletic (McKenna et al. 2014) (Fig. 2).

The homalochilous Rutelinae (Anomalini and Rutelini) were polyphyletic, with *Oryctomorphus* (Rutelini) falling into a clade including Anatistini and Anoplognathini (McKenna et al. 2014). Three dynastines were included: *Dynastes*, *Cyclocephala*, and *Peltonotus* (McKenna et al. 2014). *Cyclocephala* was recovered in a clade along with *Dynastes* (McKenna et al. 2014) (Fig. 2). However, *Peltonotus* was recovered in a sister clade that included the remaining homalochilous rutelines (*Popillia* and *Parastasia*) (McKenna et al. 2014) (Fig. 2). These results suggest that Cyclocephalini is correctly classified in Dynastinae, but that the tribe is polyphyletic if it includes *Peltonotus*. This phylogenetic analysis is more in line with the placement of *Peltonotus* near the Asian parastasiine rutelines by Arrow (1908, 1910) than the hypotheses of Jameson (1998).

**Figure 3.**
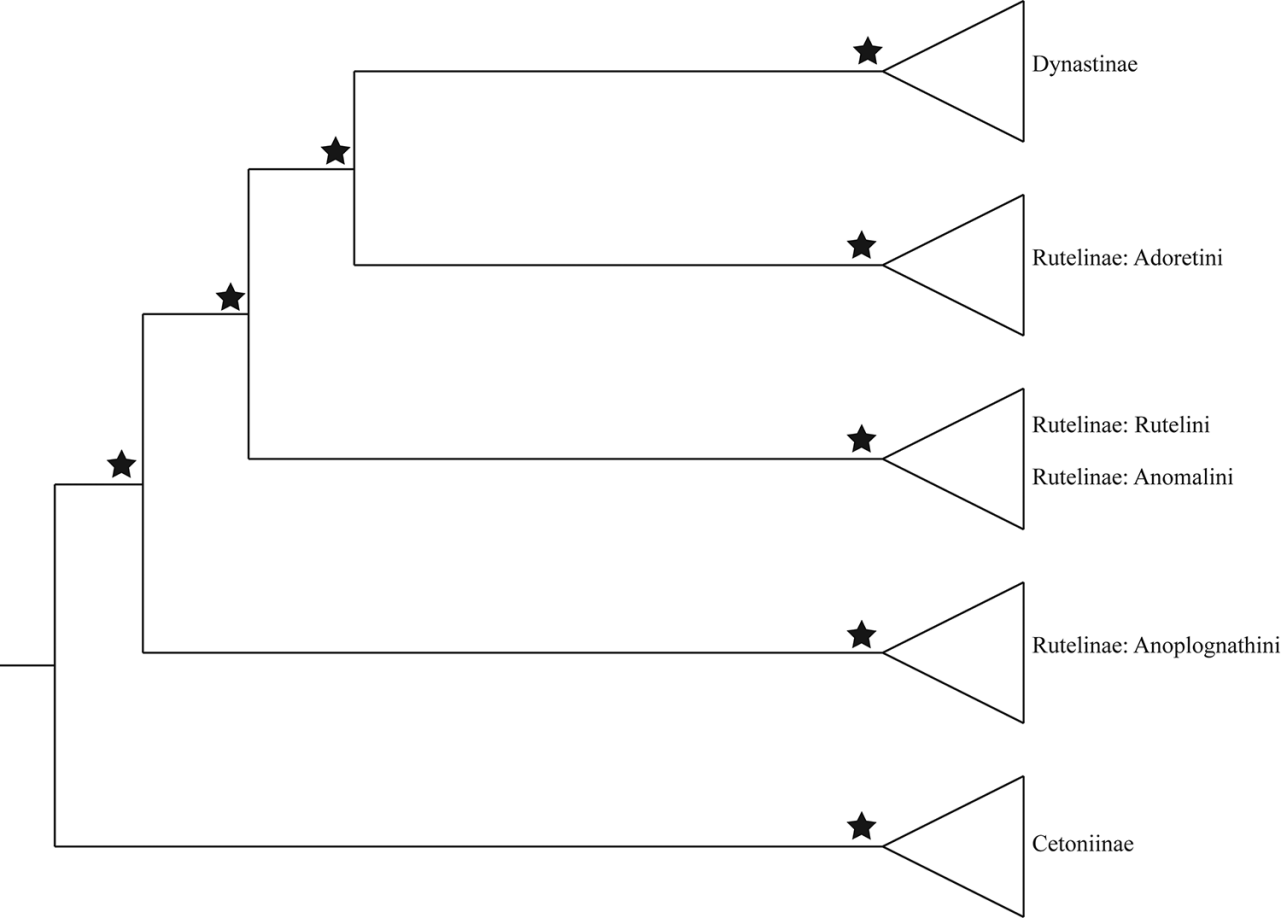
Summary of the hypothetical relationships of Rutelinae and Dynastinae from Gunter et al. (2016). Stars indicate nodes with >0.95 posterior probability.

Gunter et al. (2016), building on the datasets of Ahrens et al. (2011, 2014), utilized 16S, 12S, CO1, and 28S to conduct a phylogenetic analysis of Scarabaeoidea that included over 400 taxa. A clade including Cetoniinae + (Dynastinae and Rutelinae) was recovered, but the node uniting these subfamilies was only weakly supported (0.89 posterior probability) (Gunter et al. 2016) (Fig. 3). These three analyses, built from similar datasets, together suggest that Rutelinae is a paraphyletic grade of tribes (Ahrens et al. 2011, 2014, Gunter et al. 2016). The subfamily Dynastinae in these analyses was consistently recovered as the most derived of all scarabaeoids (Ahrens et al. 2011, 2014, Gunter et al. 2016). Gunter et al. (2016) recovered a strongly supported node that suggests that the Asian orthochilous ruteline tribe Adoretini is sister to a monophyletic Dynastinae. This node had been similarly recovered by Ahrens et al. (2011). However, this relationship between Adoretini and Dynastini was weakly supported and interrupted by Pachydemini (Melolonthinae) in Ahrens et al. (2014). McKenna et al. (2014) did not include exemplars from Adoretini, making this relationship difficult to evaluate.

**Figure 4.**
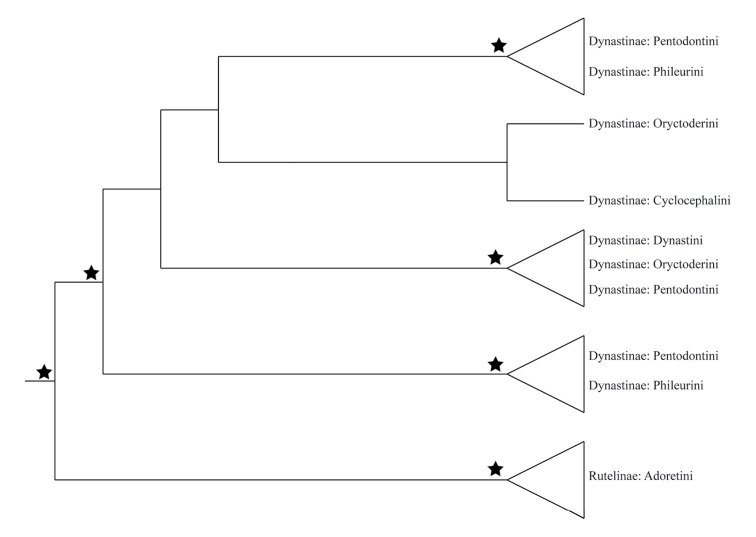
Summary of the hypothetical relationships of dynastine tribes from Gunter et al. (2016). Stars indicate nodes with >0.95 posterior probability.

The analysis by Gunter et al. (2016) included 22 dynastine species from 18 genera in 5 tribes. Nodes were generally poorly supported within Dynastinae, making it difficult to assess relationships among tribes (Gunter et al. 2016) (Fig. 4). The study included one *Cyclocephala* species, which was recovered as sister to *Onychionyx* (Oryctoderini), but this relationship was weakly supported (0.83 posterior probability). These results suggest future analyses of Cyclocephalini should include oryctoderine genera (nearly all of which were at some point previously included in Cyclocephalini) to assess the boundaries of the two tribes. Additionally, these analyses do not support the monophyly of the tribes Oryctoderini, Pentodontini, and Phileurini (Gunter et al. 2016).

Taken together, these studies demonstrate that the position of Cyclocephalini in the broader phylogeny of Dynastinae and Rutelinae is not resolved. In addition, very little is known about the relationships among cyclocephaline genera and species. Breeschoten et al. (2013) presented a morphological phylogeny of cyclocephaline genera, but few details of the analysis were provided and the support for recovered relationships were not reported. Moore et al. (2015) suggested that *Mimeoma* species were nested among a clade of *Cyclocephala* that included the type species of the genus, *C. amazona*. These data also provided evidence of two major clades of *Cyclocephala* based on morphological and molecular evidence (Moore et al. 2015). However, the relationship of *Cyclocephala* to the other cyclocephaline genera is completely unevaluated.

## Generic overviews

The section below summarizes information on the distribution, recognition, and hypothesized relationships of cyclocephaline scarab beetle genera. The provided diagnoses are roughly parallel to each other and, in many cases, discuss morphological characters that have not been adequately described for the group. Diagnoses also rely on the dissection of the mandibles, maxillae, and hindwings. These diagnoses should allow for enhanced identification when in doubt of generic-level affinities. The last identification key to genera for the tribe did not include *Peltonotus* (Jameson et al. 2002). The key to genera below builds on the work of Jameson et al. (2002) and is supplemental to that identification tool. This key requires dissection of the hindwings and mouthparts and will aid in precise identification of these groups, along with provided diagnoses.

### Key to the Adults of the World Genera of Cyclocephalini (Scarabaeidae: Dynastinae)

**Figures 5–8.**
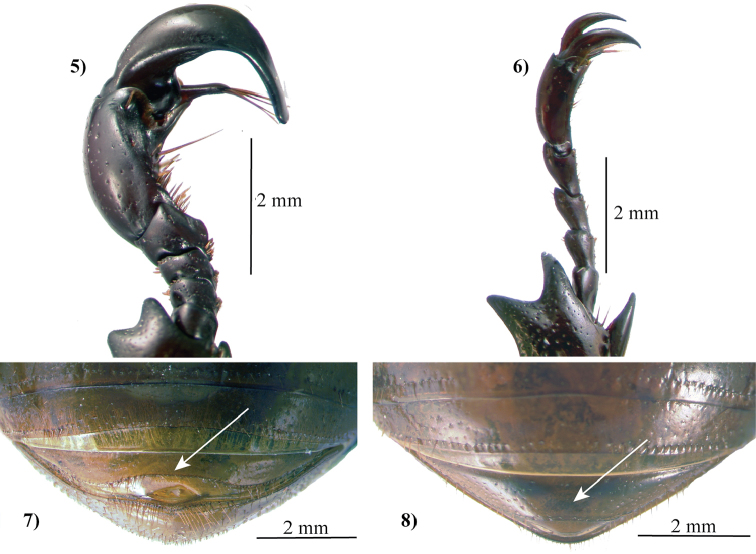
Gender specific characteristics of cyclocephaline species. **5)** *Surutu dytiscoides* Martínez; male protarsus. **6)** *S. dytiscoides*; female protarsus. **7)** *Cyclocephala conspicua* Sharp; male, last abdominal sternite emarginate. **8)** *C. conspicua* Sharp; female, last abdominal sternite entire.

Males: Protarsomeres and inner protarsal claws enlarged except for in the genera *Stenocrates* and *Erioscelis* (Fig. 5). Last abdominal sternite emarginate (Fig. 7).

Females: Protarsomeres and inner protarsal claws simple, not enlarged (Fig. 6). Last abdominal sternite entire, not emarginate (Fig. 8).

**Table:** 

1	Labrum extended anteriorly beyond the apex of the clypeus (Fig. 9). Hindwings with membranous areas pigmented and darkened (Fig. 18). Maxillae with an articulated tooth on the galea (Fig. 25). India, southern China, Southeast Asia, and Melanesia	***Peltonotus* Burmeister**
–	Labrum not extended anteriorly beyond apex of the clypeus (Figs 10–17). Hindwings with membranous areas lacking pigment, not darkened (Fig. 19). Maxillae lacking an articulated tooth on the galea (Fig. 26). Africa and the New World	**2**
2	Hindwings on leading edge distal to apical hinge with row of long erect setae with their origin at or proximal to the apical hinge (Figs 22–23) or lacking setae and lacking membrane distal to apical hinge (Fig. 26). Maxillary galea with 2-2-2 or 2-2-1 (from base to apex, most basal tooth bifurcate) teeth arrangement	**3**
–	Hindwings on leading edge distal to apical hinge lacking setae and with a membranous border (Figs 20–21) or having a row of decumbent setae arising distal to apical hinge (Figs 26–27). Maxillary galea lacking teeth or with teeth in any other arrangement	**6**
3	Vein RA with double row of pegs (second row begins mid-way along vein). Veins RA 3 and RA 4 contiguous at their base (Fig. 28). Protibiae tridentate or bidentate. Maxillary galea with 2-2-2 (six total teeth) or 2-2-1 (five total teeth) teeth arrangement	***Erioscelis* Burmeister**
–	Vein RA with single row of pegs. Veins RA 3 and RA 4 separated at their bases and not contiguous (Fig. 29). Protibiae tridentate. Maxillary galea with 2-2-2 teeth arrangement	**4**
4	Lateral margin of metacoxae simple, lacking longitudinal sulcus (Fig. 31). Meso- and metatibia dorsoventrally flattened and laterally expanded (Fig. 32). Mandibular molar area planar, lacking rounded depressions on distal portion (Fig. 36)	***Stenocrates* Burmeister**
–	Lateral margin of metacoxae with longitudinal sulcus (Fig. 30). Meso- and metatibia not strongly dorsoventrally flattened (Fig. 33). Mandibular molar area with rounded depressions on distal portion (Fig. 37)	**5**
5	Propygidium and the pygidium fused. Propygidium expanded (Figs 38–39) or not. Males with inner protarsal claw enlarged and entire at apex	***Chalepides* Casey**
–	Propygidium not expanded and not fused with the pygidium (Figs 40–41). Males with inner protarsal claw enlarged and narrowly split at apex	***Dyscinetus* Harold**
6	Vein RA with single row of pegs	**7**
–	Vein RA with double row of pegs	**10**
7	Hindwing on leading edge distal to apical hinge lacking setae and with a membranous border (Figs 20–21). Maxillary teeth on galea lacking or reduced to small spines. Maxillary galea with 5 teeth in 3-1-2 arrangement if teeth are well-developed. Meso- and metatibiae with apices straight, not corbeled (Figs 32–33, 35)	**8**
–	Hindwing on leading edge distal to apical hinge with decumbent setae arising distal to apical hinge (Figs 26–27). Membranous border lacking on leading edge of hindwing. Maxillary galea with more than 5 total teeth. Meso- and metatibiae with corbeled apices (Fig. 34)	**9**
8	Mentum with apex weakly emarginate (emargination does not approach level of labial palp insertion). Maxillary galea with well-developed teeth in 3-1-2 arrangement (Fig. 25). Veins RA3 and RA4 contiguous at their base (Figs 18, 28). Afrotropics	***Ruteloryctes* Arrow**
–	Mentum with apex deeply emarginate (emargination reaching level of labial palp insertion). Maxillary galea lacking well-developed teeth and teeth small and spinose when present. Veins RA3 and RA4 separated at their bases and not contiguous (Figs 19, 29). Neotropics	***Ancognatha* Erichson**
9	Apex of mentum deeply emarginate (Fig. 44). Anterior marginal bead of pronotum incomplete at middle (Fig. 43). Protibia straight (Fig. 47). Protibial spur articulated, not fused to protibia (Fig. 47). Males with protrochanters not produced into ventral spines. Mandibular molar area with rows of large, circular pits (Fig. 36)	***Surutu* Martínez**
–	Apex of mentum straight (Fig. 45). Anterior marginal bead of the pronotum complete at middle (Fig. 42). Males with protibia arcuate (Fig. 46). Males with protibial spur fused to protibia (Fig. 46). Males with protrochanters produced into ventral spines (Fig. 48). Mandibular molar area with rows of small micropunctures, lacking larger circular punctures	***Harposceles* Burmeister**
10	Apices of meso- and metatibiae produced into acute teeth (Figs 49–50). Males with many large, circular sensillae on the antennal club. Mesocoxae touching, not widely separated	***Acrobolbia* Ohaus**
–	Apices of meso- and metatibiae straight or weakly corbeled, not produced into acute teeth (Figs 32–35). Males lacking large sensillae on the antennal club. Mesocoxae touching or widely separated	**11**
11	Metatibiae lacking raised, transverse carinae (Fig. 35). Dorsal coloration with a mother-of-pearl sheen or not. Mesocoxae widely separated, not touching. Clypeus with apex evenly rounded (Fig. 10)	***Augoderia* Burmeister**
–	Metatibiae with at least one raised, transverse carina (Fig. 32–34). Dorsal coloration lacking a mother-of-pearl sheen. Mesocoxae widely separated or not. Clypeus with apex rounded, parabolic, truncate, emarginate, acute, or bisinuate (Figs 11–17)	**12**
12	Body anteroposteriorly compressed and having a round gestalt. Clypeus with apex truncate and straight, appearing quadrate in dorsal view (Fig. 11). Clypeus with apex curved upward, creating a small depression on disc. Mesocoxae widely separated, not touching. Both sexes with tridentate protibiae, proximal most tooth reduced in size and removed from two distal teeth. Protibial spur straight to weakly decurved. Metacoxae with lateral surface perpendicular with respect to ventral surface	***Arriguttia* Martínez**
–	Body not anteroposteriorly compressed and having an oval gestalt. Clypeus with apex rounded, parabolic, truncate, emarginate, acute, or bisinuate (Figs 12–17). Clypeal apex planar with base of clypeus, not strongly curved upward. Mesocoxae widely separated or not. Males with protibiae tridentate or bidentate. Females with tridentate protibiae. Protibial spur straight to weakly decurved or strongly decurved. Metacoxae with lateral surface perpendicular with respect to ventral surface or angled beneath ventral surface	**13**
13	Clypeus with sides weakly divergent to straight at base (Fig. 12). Clypeal apex nearly straight across or broadly rounded, never acute or emarginate. Maxillae with galea strongly dorsoventrally flattened into rounded lobe lacking well-developed teeth (except for *Aspidolea fuliginea*). Apex of maxillae with tight, dense brush of long, penicillate setae	***Aspidolea* Bates**
–	Clypeus with sides convergent at base (except for species similar to *Cyclocephala porioni*) (Fig. 15). Clypeal apex acute, parabolic, broadly rounded, emarginate, truncate, or bisinuate. Maxillae with galea dorsoventrally flattened or not, but usually with well-developed teeth in many different arrangements. Apex of maxillae without tight, dense brush of long, penicillate setae	***Cyclocephala* Dejean**

**Figures 9–17.**
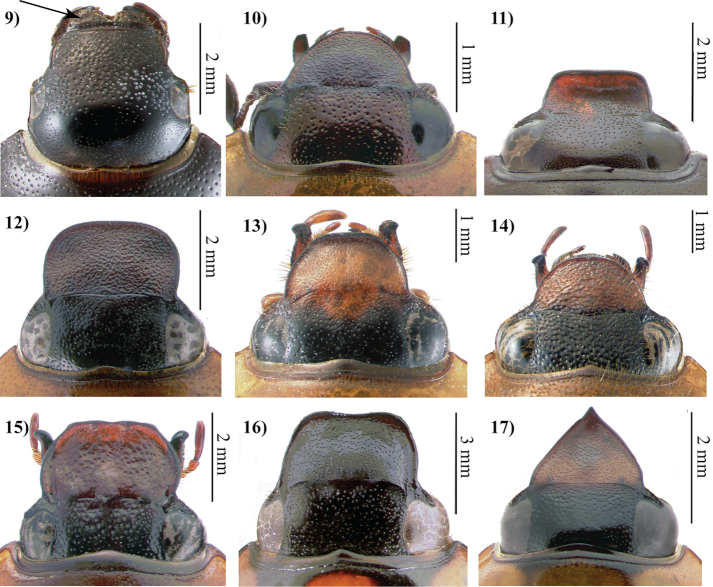
Clypeal and labral form of cyclocephaline species. **9** *Peltonotus malayensis* Arrow; black arrow indicates the anteriorly produced labrum **10** *Augoderia nitidula* Burmeister; clypeus rounded **11** *Arriguttia brevissima* (Arrow); clypeus truncate and apex strongly reflexed dorsally **12** *Aspidolea singularis* Bates; clypeus broadly rounded and with lateral margins slightly divergent at base **13** *Cyclocephala weidneri* Endrődi; clypeus truncate without apex strongly reflexed dorsally **14** *Cyclocephala octopunctata* Burmeister; clypeus rounded **15** *Cyclocephala hartmannorum* Malý; clypeus bisinuate and with lateral margins divergent at base **16** *Cyclocephala mafaffa* Burmeister; clypeus emarginate **17** *Cyclocephala acuta* Arrow; clypeus acute.

**Figures 18–23.**
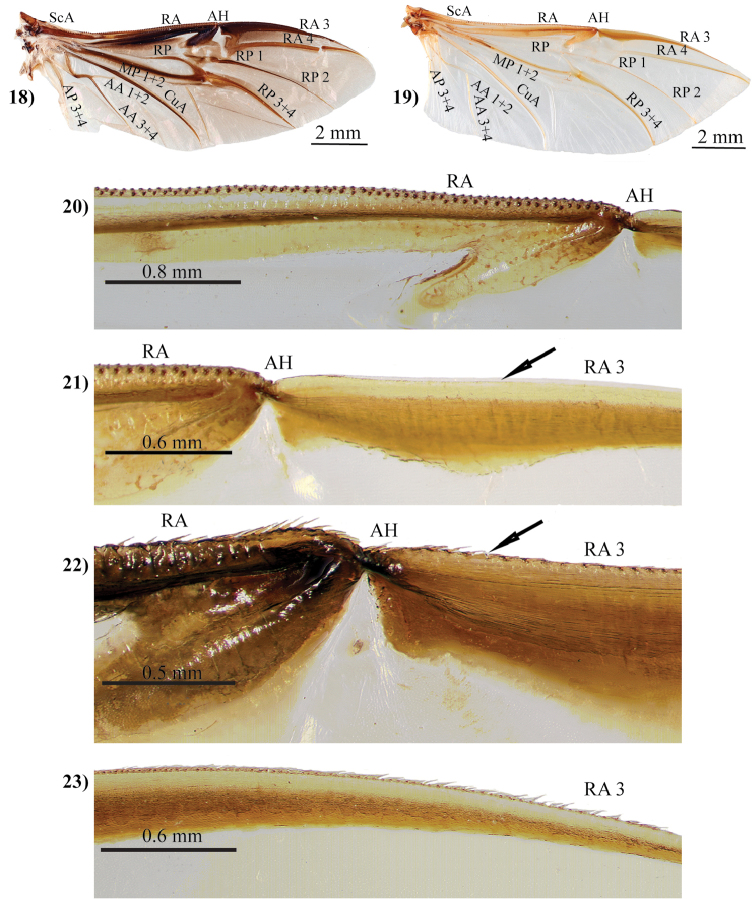
Hindwings of cyclocephaline species. **18** *Peltonotus nasutus* Arrow; labeled veins of the hindwing **19** *Cyclocephala amazona* (Linnaeus); labeled veins of the hindwing **20** *C. amazona*; view of vein RA proximal to AH showing lack of setae and double row of pegs **21** *C. amazona*; view of vein RA 3 distal to AH showing lack of setae. Arrow indicates membranous border of RA 3 **22** *Chalepides barbatus* (Fabricius); view of veins RA and RA 3 showing presence of setae proximally and distally from AH. Arrow indicates the presence of setae along RA 3 **23** *C. barbatus*; view of vein RA 3 distal to AH showing erect row of setae along the vein. Abbreviations: AA=Anal anterior vein; AP=Anal posterior vein; AH=Apical hinge of hind wing; CuA=Cubitus anterior vein; MP=Medial posterior vein; RA=Radius anterior vein; RP=Radius posterior vein; ScA=Subcosta anterior vein.

**Figures 24–25.**
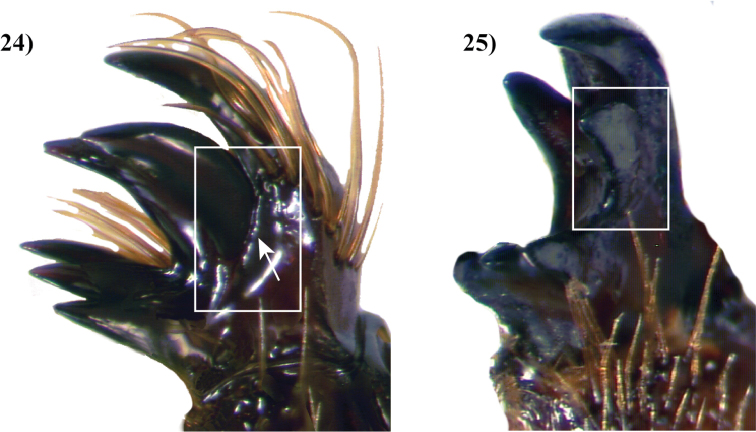
Galea of maxillae in *Peltonotus* and *Ruteloryctes*. **24** *Peltonotus nasutus* Arrow; galea of maxilla with articulated tooth indicated by arrow **25** *Ruteloryctes morio* Fabricius; galea of maxilla lacking articulated tooth.

**Figures 26–27.**
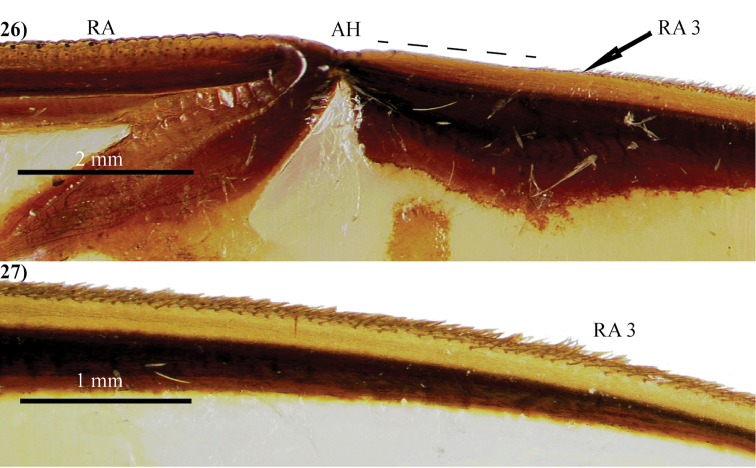
Leading edge of the hindwing in *Harposceles paradoxus* Burmeister. **26** *H. paradoxus*; distribution of setae on the leading edge of the hindwing. Arrow indicates setae on the edge of RA 3. Dashed line indicates glabrous area directly distal to AH **27** *H. paradoxus*; view of the decumbent setae of vein RA 3. Abbreviations: AH=Apical hinge of hind wing; RA=Radius anterior vein.

**Figures 28–29.**
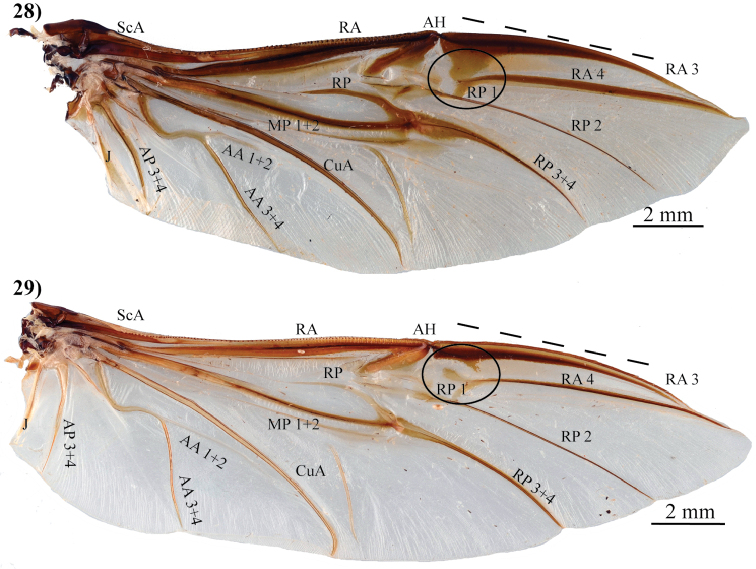
Hindwings of *Erioscelis emarginata* (Mannerheim) and *Stenocrates clipeatus* Endrődi. **28** *E. emarginata*; hindwing showing the veins RA 4 and RA 3 contiguous at their bases, indicated by the circle. Dashed line indicates glabrous region of RA 3 **29** *S. clipeatus*; hindwing showing veins RA 4 and RA 3 separated at their bases, indicated by the circle. Dashed line indicates row of erect setae along length of RA 3.

**Figures 30–35.**
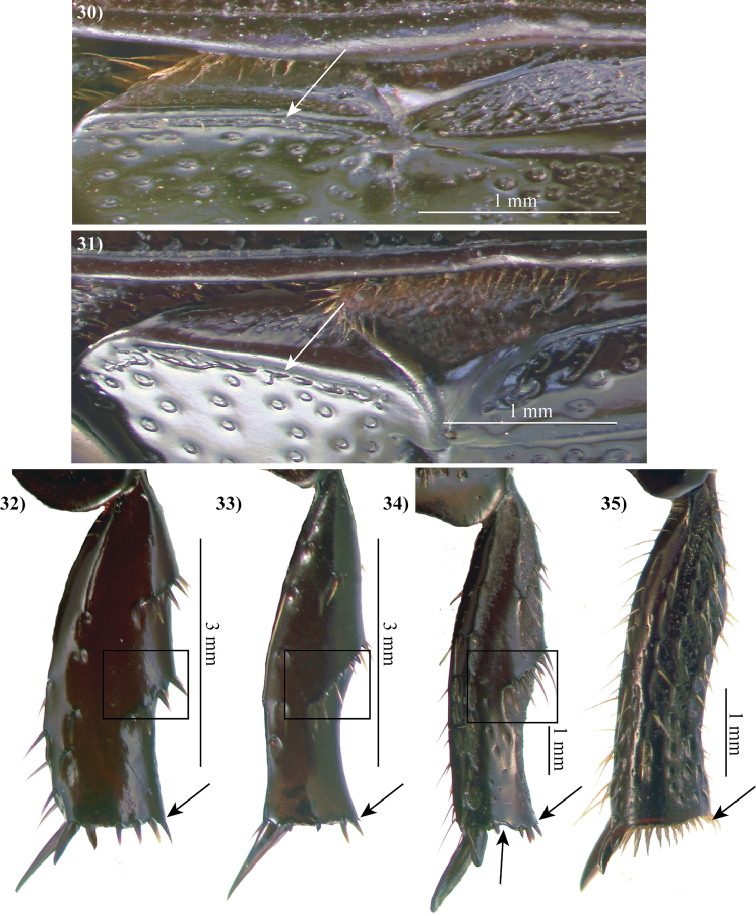
Metacoxal and metatibial morphology of cyclocephaline species. **30** *Dyscinetus morator* (Fabricius), metacoxa. White arrow indicates transverse sulcus on the lateral edge on the ventral surface of the metacoxa **31** *Stenocrates canuli* Delgado, metacoxa. White arrow indicates punctation on the lateral edge on the ventral surface of the metacoxa **32** *S. canuli*, metatibia. Arrow indicates the straight apex of the metatibia. Square indicates transverse carina **33** *Dyscinetus laevicollis* Arrow, metatibia. Arrow indicates the straight apex of the metatibia. Square indicates transverse carina **34)** *Surutu dytiscoides* Martínez, metatibia. Arrows indicate the corbeled apex of the metatibia. Square indicates transverse carina **35** *Augoderia nitidula* Burmeister, metatibia. Arrow indicates the straight apex of the metatibia.

**Figures 36–37.**
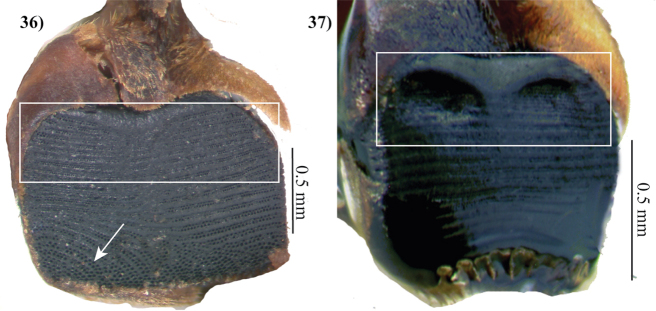
Mandibular molar of *Cyclocephala kaszabi* Endrődi and *Dyscinetus laevipunctatus* Bates. **36** *C. kaszabi*; white box indicates the lack of depressions on distal portion of molar. Arrow indicates large circular punctures compared to micropunctures on the rest of the molar **37** *D. laevipunctatus*; white box indicates rounded depressions on the distal portion of the molar.

**Figures 38–41.**
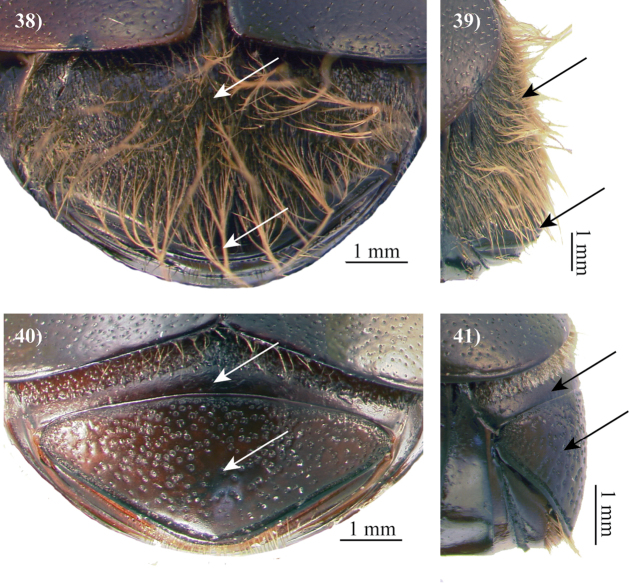
Pygidial morphology of *Dyscinetus* and *Chalepides* species **38** *Chalepides alliaceus* Burmeister; apex of the abdomen in caudal view. Top arrow indicates the propygidium. Bottom arrow indicates the reduced pygidium **39** *C. alliaceus*; apex of the abdomen in lateral view. Top arrow indicates the propygidium. Bottom arrow indicates the reduced pygidium **40** *Dyscinetus laevicollis* Arrow; apex of the abdomen in caudal view. Top arrow indicates the propygidium. Bttom arrow indicates the pygidium **41** *D. laevicollis*; apex of the abdomen in lateral view. Top arrow indicates the propygidium. Bottom arrow indicates the pygidium.

**Figures 42–45.**
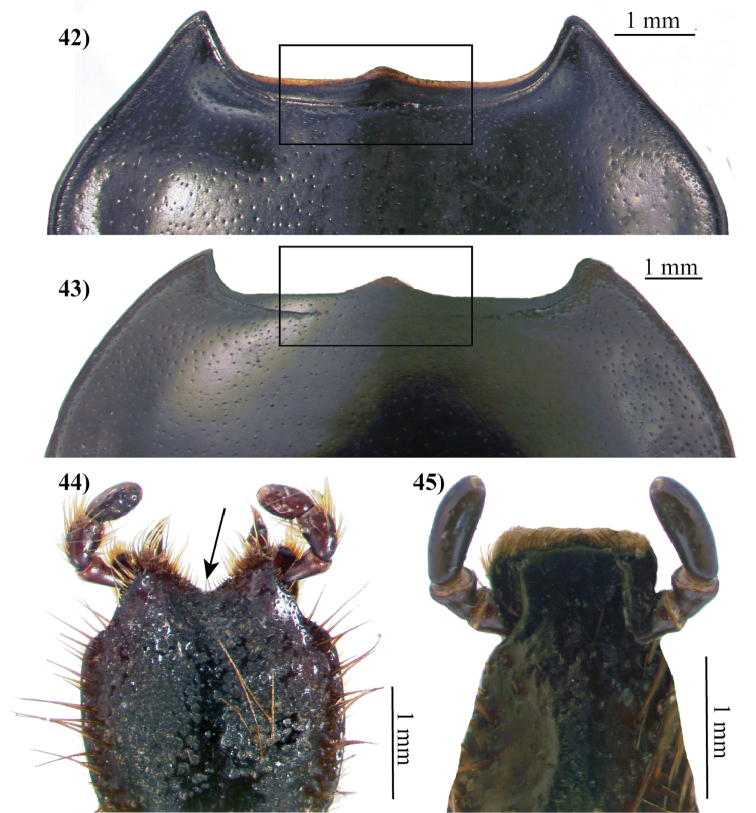
Pronotum and labium morphology of *Harposceles paradoxus* Burmeister and *Surutu dytiscoides* Martínez. **42** *H. paradoxus*; anterior margin of pronotum. Box indicates the complete marginal bead **43** *S. dytiscoides*; anterior margin of pronotum. Box indicates the incomplete marginal bead **44** *S. dytiscoides*; apex of the mentum. Arrow indicates the deeply emarginate apex of the mentum **45** *H. paradoxus*; apex of the mentum.

**Figures 46–48.**
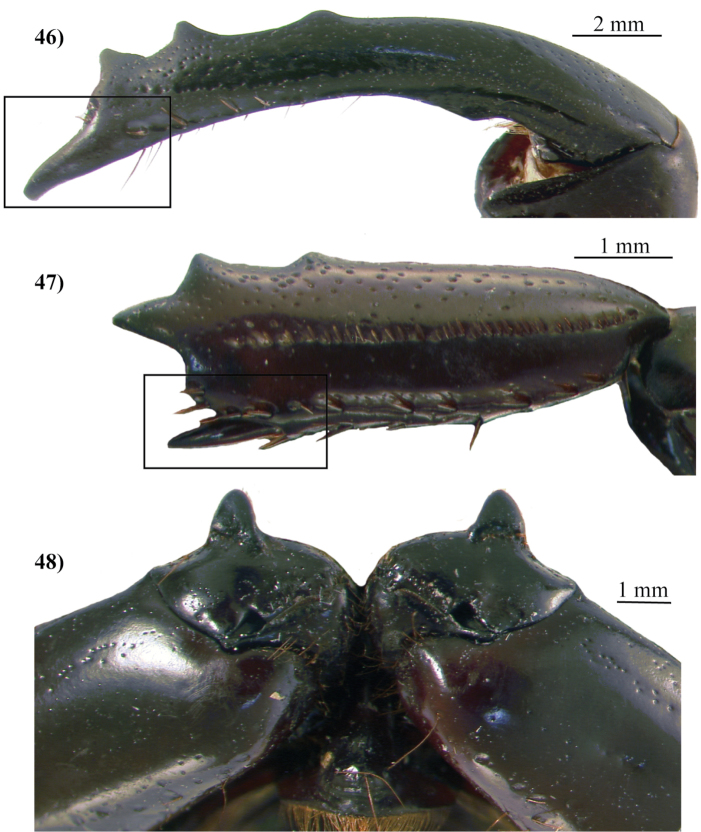
Proleg morphology of *Harposceles paradoxus* Burmeister and *Surutu dytiscoides* Martínez. **46** *H. paradoxus*; arcuate protibia of male. Box indicates the fusion of the protibial spur to the protibia **47** *S. dytiscoides*; protibia. Box indicates the articulated protibial spur. **48** *H. paradoxus*; spines of the protrochanter.

**Figures 49–50.**
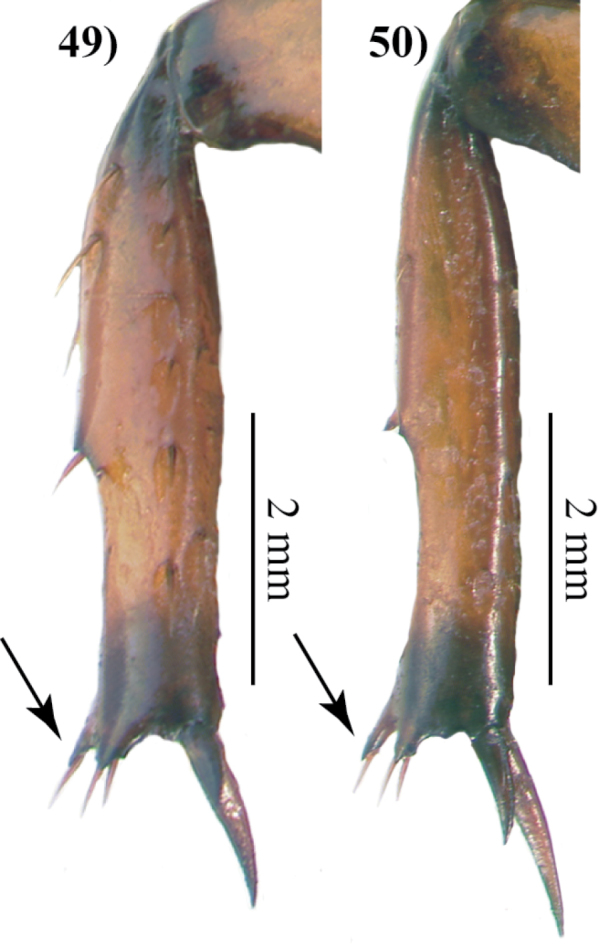
Meso- and metatibia of *Acrobolbia macrophylla* Ohaus. **49** *A. macrophylla*; mesotibia. Arrow indicates the acute, spine-like apices **50** *A. macrophylla*; metatibia. Arrow indicates the acute, spine-like apices.

#### Acrobolbia

Taxon classificationAnimaliaColeopteraScarabaeidae

Ohaus, 1912

##### Type species.

*Acrobolbia macrophylla* Ohaus, 1912, by monotypy.

##### Valid taxa.

One species.

**Figure 51.**
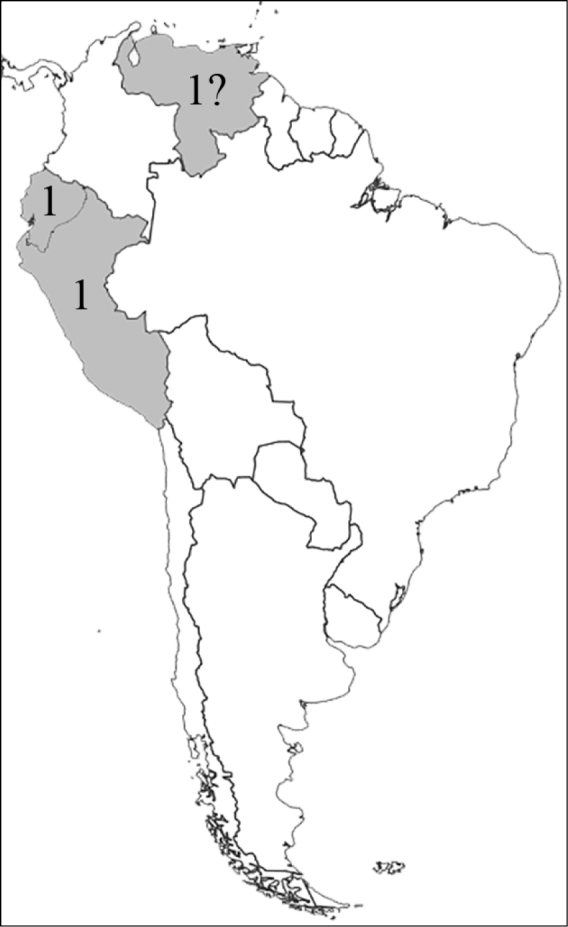
Country-level distribution of *Acrobolbia macrophylla* in South America. Numbers indicate taxa per country. The presence of *A. macrophylla* in Venezuela is based upon a single specimen without further label details.

The northern South American genus *Acrobolbia* is known from Peru, Ecuador, and possibly Venezuela (Ohaus 1912, Machatschke 1972, Jameson et al. 2002) (Fig. 51). *Acrobolbia* has a complicated classification history. Ohaus (1912) described *A. macrophylla* based upon a single male specimen collected in Peru. Ohaus (1912) compared *Acrobolbia* to *Cyclocephala*, but he ultimately classified the genus in the subtribe Areodina (Rutelinae: Rutelini). Ohaus (1918) later transferred the genus into its own subtribe, Acrobolbiina, within Rutelini. *Acrobolbia triangularis* was the second species to be described into the genus, but this species was later treated as a synonym and a “variant” of *A. macrophylla* (Benderitter 1922, Ohaus 1934a, b).

Based on the elongated antennal club of the male in *Acrobolbia*, the genus was transferred into the ruteline subtribe Oryctomorphina (Dechambre and Ponchel 1999). Most recently, *Acrobolbia* was reviewed and transferred into Cyclocephalini by Jameson et al. (2002). *Acrobolbia* is hypothesized to be related to *Ancognatha* based upon characters of the clypeus, mentum, pronotum, prosternal process, protarsus, and mandibles (Jameson 1998, Jameson et al. 2002). Specimens of *Acrobolbia* are rare in collections, and almost nothing is known of their biology (Jameson et al. 2002). *Acrobolbia macrophylla* adults are attracted to lights at night, though specimens do not land or rest at light traps (Jameson et al. 2002). Specimens have been collected from 400–1,200 m in elevation (Jameson et al. 2002). The immature stages are undescribed and unknown.

*Acrobolbia* species can be recognized by the following combination of characters: 1) dorsal coloration varying from all black with variable reddish brown margins of the elytra and elytral suture, or with the elytra partially testaceous; 2) body not anteroposteriorly compressed or dorsoventrally flattened; 3) clypeal apex acuminate in dorsal view; 4) frontoclypeal suture distinct, but incomplete medially; 5) mandibles long, sickle-shaped, with pointed apex; 6) mandibular molar area with rows of circular micropunctures; 7) apical margin of mentum weakly emarginate to nearly straight; 8) galea of maxilla reduced to small, rectangular mound in dorsal view; 9) galea on inner surface with teeth greatly reduced to peg-like projections at the middle and apex; 10) galea on inner surface lacking teeth at base; 11) males with antennal club (segments 8–10) elongated, nearly twice as long as antennomeres 1–7; 12) pronotum with broadly incomplete beaded basal margin; 13) males and females with 3 protibial teeth, basal tooth reduced, removed from the apical 2 teeth, and oriented laterally; 14) protibial spur straight to weakly deflexed; 15) males with inner protarsal claw enlarged and narrowly cleft at apex; 16) mesocoxae touching, nearly contiguous; 17) meso- and metatibiae with distal, divided carinae; 18) metacoxae with lateral edge perpendicular to ventral surface; 19) anterior edge of hindwing distal to apical hinge lacking setae and with produced, membranous border; 20) vein RA with 2 rows of pegs extending distally nearly to margin of apical hinge.

#### Ancognatha

Taxon classificationAnimaliaColeopteraScarabaeidae

Erichson, 1847

##### Type species.

*Ancognatha scarabaeoides* Erichson, subsequent designation by Casey 1915: 111.

##### Valid taxa.

22 species.

**Figure 52.**
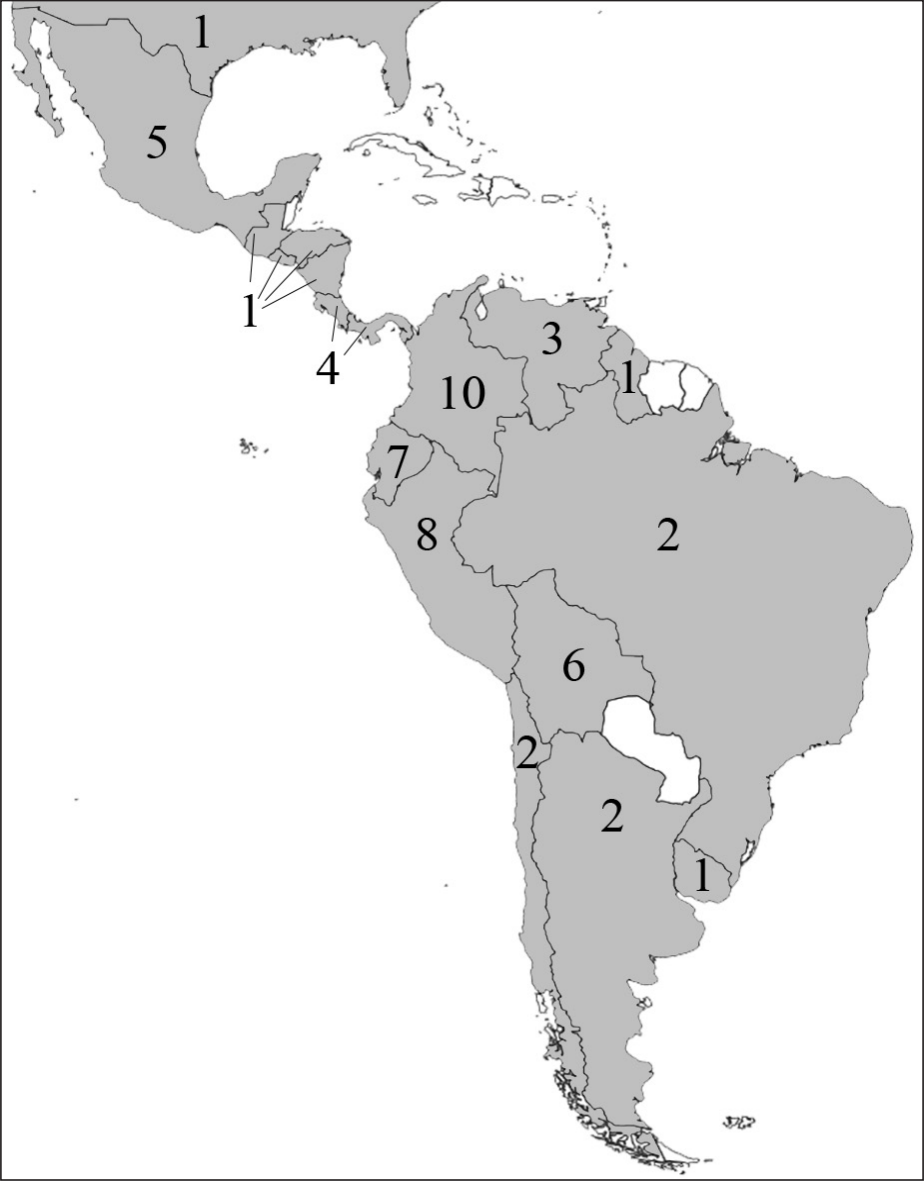
Distribution of *Ancognatha* taxa in North, Central, and South America. Numbers indicate taxa per country.

The 22 species of *Ancognatha* are distributed from the southwestern United States south to Argentina (Fig. 52). The species diversity in the genus is concentrated in north and western South America and in Mexico, west of the Isthmus of Tehuantepec. Biological information on *Ancognatha* species is lacking, and almost nothing is known about the natural history of adults. In Meso- and Central America, *Ancognatha* species are associated with premontane, lower montane, and montane tropical forests with some species being collected at elevations from 2,000 to 3,500 m above sea level (Ratcliffe 2003, Ratcliffe and Cave 2006, Ratcliffe et al. 2013). This pattern also holds in South America. Several *Ancognatha* species have been recorded from elevations over 4,000 m in Peru and northern Chile (Mondaca 2016, Figueroa and Ratcliffe 2016). Some South American *Ancognatha* species can be very large for the tribe. For example, *A. matilei* Dechambre from Colombia is up to the 36 mm long (Dechambre 2000). Adults are attracted to lights at night.

Larvae are described for four *Ancognatha* species (Ritcher 1966, Ramírez-Salinas et al. 2004, Vallejo and Morón 2008, Neita-Moreno and Morón 2008). South American larval descriptions are largely based on material collected in agroecosystems, and thus the natural ecology of *Ancognatha* immatures is poorly known. Mondaca (2016) reported the larvae of *A. aymara* Mondaca feeding on grass roots high in the altiplano steppe of northern Chile.

*Ancognatha* species can be recognized by the following combination of characters: 1) dorsal coloration variable, from all or partially black or testaceous, to light brown with variable dark maculae; 2) body convex and not strongly anteroposteriorly or dorsoventrally compressed; 3) clypeal apex rounded to parabolic, never truncate or emarginate; 4) frontoclypeal suture incomplete medially; 5) males with anterolateral margin of the mandibles without teeth; 6) mandibular apices narrow and elongated, recurved dorsally; 7) mandibular molar area with rows of circular micropunctures; 8) apical margin of mentum narrowly and deeply emarginated; 9) galea of maxilla reduced to a roughly quadrate process; 10) galea of the maxilla on inner surface lacking well-developed teeth, teeth when present and visible greatly reduced into spine-like projections; 11) males and females with 3 protibial teeth, basal tooth slightly removed from the more apical 2 teeth, and oriented laterally; 12) protibial spur straight to weakly deflexed; 13) males with inner protarsal claw enlarged and narrowly cleft at apex; 14) mesocoxae narrowly separated and touching; 15) meso- and metatibiae with distal, transverse carinae; 16) metacoxae with lateral edge perpendicular to ventral surface; 17) anterior edge of hindwing distal to apical hinge lacking setae and with produced, membranous border; 18) vein RA with single row of pegs extending distally nearly to margin of apical hinge; 19) elytral margin membranous.

The relationship of *Ancognatha* species to other cyclocephaline genera has not been evaluated. *Acrobolbia* may be related to *Ancognatha* based on characters of the clypeus, mentum, pronotum, prosternal process, protarsus, and mandibles (Jameson 1998, Jameson et al. 2002). *Surutu* also shares some intriguing characters with *Ancognatha*, which may be indicative of a close relationship between these two genera. For example, *Ancognatha* and *Surutu* species all have a rounded to parabolic clypeal apex and a narrowly, but deeply, emarginated apex of the mentum. *Surutu* species have a anteriorly projecting tooth at the apex of the labrum, and this is also shared in some *Ancognatha* species.

#### Arriguttia

Taxon classificationAnimaliaColeopteraScarabaeidae

Martínez, 1960

##### Type species.

*Cyclocephala brevissima* Arrow, 1911, by monotypy.

##### Valid taxa.

Two species.

**Figure 53.**
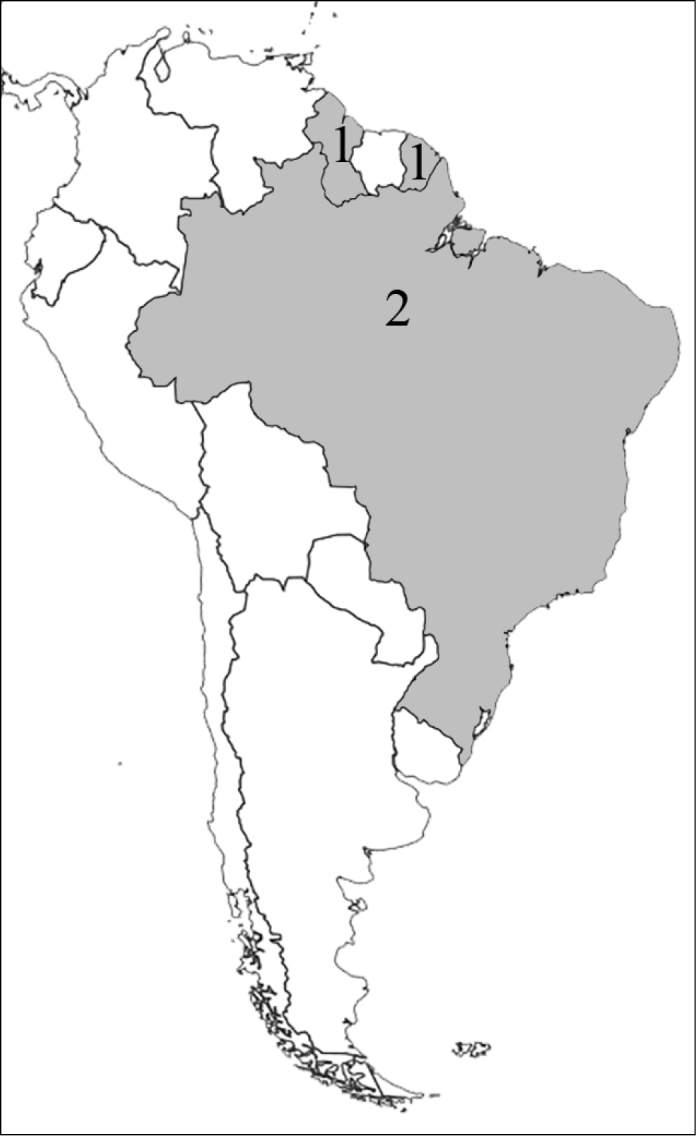
Country-level distribution of *Arriguttia* taxa in South America. Numbers indicate taxa per country.

*Arriguttia* contains two South American species known only from the Brazilian Amazon, Guyana, and French Guiana (Arrow 1911, 1937b, Blackwelder 1944, Martínez 1960a, 1968a, Endrődi 1966, 1985a, Ponchel 2006, 2011, 2015) (Fig. 53). Very little is known about the biology of *Arriguttia* species. *Arriguttia brevissima* (Arrow) feeds within the inflorescences of *Victoria* sp. in Brazil (Martínez 1968a). In French Guiana, *A. brevissima* was found in the spathes of an unidentified terrestrial aroid (Araceae) (Ponchel 2006, 2015). In Brazilian cerrado habitat, *A. brevissima* are floral visitors of *Annona coriacea* Mart. and are likely late-season, secondary pollinators of this species (Costa et al. 2017). Specimens of *A. brevissima* have been collected at lights at night (Martínez 1968a). The immature stages are undescribed and unknown.

*Arriguttia* was compared to *Surutu* in the original description of the genus (Martínez 1960a). This is possibly confusing for identification purposes. *Arriguttia* shares many more characters with *Cyclocephala* and *Augoderia* than with *Surutu*. *Arriguttia* species can be recognized by the following combination of characters: 1) dorsal coloration varying from all black or with variable dark, reddish coloration on the elytra; 2) body convex and anteroposteriorly compressed, creating a relatively round gestalt; 3) clypeus quadrate in dorsal view, with sides nearly parallel, and the apex distinctly reflexed upwards (most obvious in lateral view); 4) frontoclypeal suture complete medially; 5) males with anterolateral margin of the mandibles weakly toothed; 6) mandibular molar area with rows of circular micropunctures; 7) apical margin of mentum weakly emarginated; 8) galea of the maxilla on inner surface with 3 fused basal teeth, a free median tooth, and 2 fused apical teeth (3-1-2 arrangement); 9) pronotum with broadly incomplete beaded basal margin; 10) males and females with 3 protibial teeth, basal tooth reduced, removed from the more apical 2 teeth, and oriented anteriorly; 11) protibial spur straight to weakly deflexed; 12) males with inner protarsal claw enlarged and narrowly cleft at apex; 13) mesocoxae widely separated; 14) meso- and metatibiae with distal, transverse carinae; 15) metacoxae with lateral edge perpendicular to ventral surface; 16) anterior edge of hindwing distal to apical hinge lacking setae and with produced, membranous border; 17) vein RA with 2 rows of pegs extending distally nearly to margin of apical hinge.

The relationships of *Arriguttia* to other cyclocephaline genera have not been clearly discussed in the literature. Martínez (1968a) stated that *Arriguttia* should be “placed next to” *Surutu*, but he did not offer any character justifications for this hypothesis. Endrődi (1966) considered *Arriguttia* to be a “primitive” cyclocephaline based on his poorly justified character analysis. *Arriguttia* shares hindwing characters (two rows of pegs on vein RA and a membrane on the leading edge of the hindwing distal to the apical hinge) with *Augoderia*, *Aspidolea*, and *Cyclocephala*. The form of the maxilla (3-1-2 teeth arrangement), the mandibular form (males with anterolateral margin weakly toothed and the molar area with rows of circular micropunctures), the incomplete bead on the basal margin of the pronotum, and the shape and arrangement of the protibial teeth are shared among *Arriguttia*, *Augoderia*, and some *Cyclocephala* (especially species like *C. sexpunctata* Laporte and species formerly placed in *Stigmalia* Casey). Future analyses should focus on comparing characters in these *Cyclocephala* species-groups and genera to *Arriguttia*, rather than *Surutu*.

#### Aspidolea

Taxon classificationAnimaliaColeopteraScarabaeidae

Bates, 1888

##### Type species.

*Aspidolea singularis* Bates, 1888: 296–297, by monotypy.

##### Valid taxa.

26 species.

**Figure 54.**
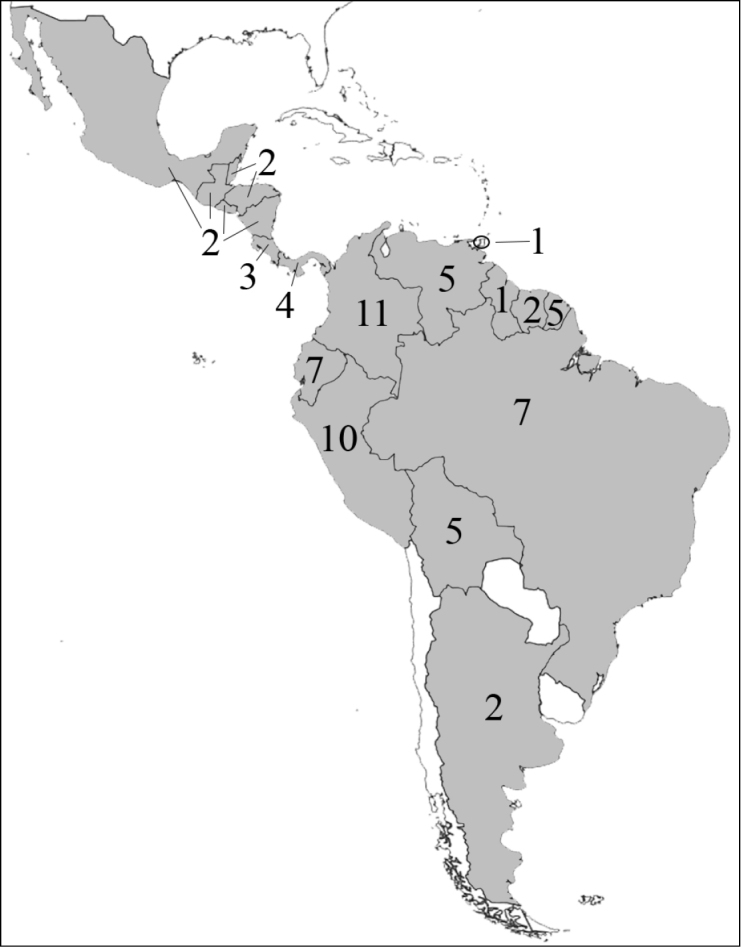
Country-level distribution of *Aspidolea* taxa in Meso-, Central, and South America. Numbers indicate taxa per country.

*Aspidolea* contains 26 species ranging from northern Mexico south through South America (Fig. 54) (Endrődi 1966, 1985a, Ratcliffe 2003, Ratcliffe and Cave 2006, Ratcliffe et al. 2013). The genus includes both widespread and narrowly distributed species. Most *Aspidolea* (22 of 26 species) are known only from a few South American localities. In contrast, *A. fuliginea* and *A. singularis* occur from Mexico south to Argentina and Ecuador, respectively. Bates (1888) described *Aspidolea* based upon the “elongate and robust” yet toothless maxillary galea found in the type species *A. singularis*. Bates (1888) noted a similar reduction in maxillary teeth in “*Cyclocephala fuliginea* Burmeister” and *Ancognatha* species. *Aspidolea* contained only *A. singularis* for over 30 years until Höhne (1922a, b, c) recircumscribed the genus and placed many new species into the group.

Höhne (1922a) offered an expanded diagnosis of *Aspidolea* using characters of the clypeus (sides parallel at base with apical margin perpendicular to the sides), maxilla (toothless and with penicillate setae at the apex), and dorsum (yellow to brownish coloration and generally lacking maculae) to distinguish the genus. *Cyclocephala clypeata* Burmeister and *C. laticeps* Harold were transferred into *Aspidolea* along with ten new species described by Höhne (1922a). The new genus *Paraspidolea* was erected to contain species similar to *Aspidolea*, but with at least two small teeth present at the apex of the galea (Höhne 1922a). Six new species were included in *Paraspidolea* along with the Burmeister species *C. fuliginea* (Höhne 1922a, b). The subgenus Aspidolea (Aspidolites) was erected to contain the species *A. atricollis* Höhne (Höhne 1923c). The homonym *Aspidolites* Höhne was replaced with *Aspidolella* (Prell 1936). *Aspidolea atricollis* is conspecific with *C. histrionica* Burmeister (Endrődi 1966), and the subgenus Aspidolella is considered a synonym of *Cyclocephala*. *Paraspidolea* was also synonymized within *Aspidolea* (Endrődi 1966).

The last major contribution to the knowledge of *Aspidolea* was provided by Dechambre (1992). Dechambre (1992) described three new *Aspidolea* species, which he included in the “*Aspidolea helleri* species-group” along with *A. helleri* (Höhne) and *A. chalumeaui* Endrődi. These species were placed into the “*helleri* species-group” based on the bidentate form of the protibial margin in males. This male protibial character is shared with species formerly included in *Mimeoma* and some *Cyclocephala* species (like *C. amazona*) (see Moore et al. 2015). The dorsal coloration of the “*helleri* species-group”, especially the elongated, triangular maculae found along the elytral suture, is like that found in some former *Mimeoma* species (especially *Cyclocephala acuta* Arrow and *C. englemani* (Ratcliffe)). These characters suggest that *Aspidolea* may not be monophyletic as presently defined.

There is little available biological data for *Aspidolea* species. *Aspidolea* adults seem to be readily attracted to lights at night and can occasionally be collected in large numbers (Ratcliffe and Cave 2006, Touroult et al. 2010, Grossi et al. 2011). Floral association data for *Aspidolea* are mostly lacking. *Aspidolea fuliginea* were collected in male- and female-phase inflorescences of *Oenocarpus bataua* Mart. (Arecaceae) in Colombia, though they were only sporadically encountered (Núñez-Avellaneda and Rojas-Robles 2008). In French Guiana, *A. quadrata* Endrődi was collected from the inflorescence of *Montrichardia arborescens* (L.) Schott (Araceae) (Gibernau et al. 2003, Ponchel 2006). Neita-Moreno et al. (2007) described the larva and pupa of *A. singularis*. Larvae of *A. singularis* were collected from soil beneath cultivated cassava (*Manihot esculenta* Crantz; Euphorbiaceae) in Colombia (Neita-Moreno et al. 2007).

*Aspidolea* species can be recognized by the following combination of characters: 1) dorsal coloration highly variable, with or without black or brown maculae on the pronotum and elytra; 2) body not anteroposteriorly compressed or dorsoventrally flattened; 3) clypeus robust and broad, with sides more or less parallel at base, appearing quadrate in dorsal view; 4) frontoclypeal suture complete medially; 5) males with anterolateral margin of the mandibles weakly toothed (in *A. fuliginea*) or not; 6) mandibular molar area with rows of circular micropunctures; 7) apical margin of mentum broadly and deeply (nearly to level of labial palp insertion) emarginated; 8) galea of maxilla dorsoventrally flattened; 9) dentition of galea of maxilla variable, inner surface of galea lacking teeth or with reduced teeth (2 small, yet obvious teeth at the apex with 1 greatly reduced tooth at the base, presence or absence of medial teeth varies among species, teeth often obscured by dense setae); 10) apex of galea with dense brush of penicillate setae; 11) pronotum with broadly incomplete or complete beaded basal margin; 12) males with 2 or 3 protibial teeth, females with 3 protibial teeth, when 3 teeth are present, basal tooth reduced, removed from the more apical 2 teeth, and oriented laterally; 13) protibial spur straight to weakly deflexed or strongly deflexed; 14) males with inner protarsal claw enlarged and entire (not cleft with a small ramus) or narrowly cleft at apex; 15) mesocoxae widely separated; 16) meso- and metatibiae with distal, transverse carinae; 17) metacoxae with lateral edge acutely angled with respect to ventral surface; 18) anterior edge of hindwing distal to apical hinge lacking setae and with produced, membranous border; 19) vein RA with 2 rows of pegs extending distally nearly to margin of apical hinge.

#### Augoderia

Taxon classificationAnimaliaColeopteraScarabaeidae

Burmeister, 1847

##### Type species.

*Augoderia nitidula* Burmeister, 1847: 34, by monotypy.

##### Valid taxa.

Five species and subspecies.

**Figure 55.**
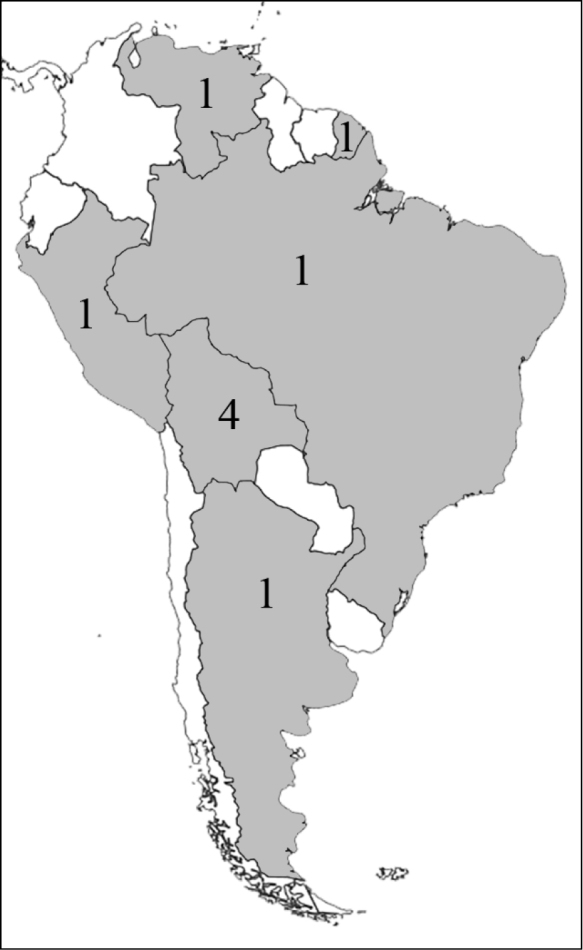
Country-level distribution of *Augoderia* taxa in South America. Numbers indicate taxa per country.

The five species and subspecies of *Augoderia* are distributed in Argentina, Bolivia, Brazil, French Guiana, Peru, and Venezuela (Burmeister 1847, Harold 1869b, Arrow 1937b, Blackwelder 1944, Guimarães 1944, Martínez 1966, Gibbs et al. 1977, Endrődi 1966, 1967a, 1981, 1985a, Riehs 2005, Ronqui and Lopes 2006, Ponchel 2009, Grossi et al. 2011, Ratcliffe et al. 2015) (Fig. 55). *Augoderia* species are similar to some *Cyclocephala* in overall appearance, although three taxa (*A. giuglarisi* Ponchel, *A. nitidula nitidula*, and *A. nitidula yungana* Martínez) are notable for their metallic, mother-of-pearl luster that reflects circularly polarized light, a cuticular trait that is rare in Dynastinae (Endrődi 1967a, 1981, Ponchel 2009, Pye 2010). The biology of *Augoderia* species is completely unknown. Gibbs et al. (1977) reported *A. nitidula* as a floral visitor of *Magnolia ovata*, but this beetle was likely a misidentified *Cyclocephala* species (see Gottsberger et al. 2012, Moore and Jameson 2013). The immature stages are undescribed. Adults are attracted to lights at night (Riehs 2005, Ronqui and Lopes 2006, Grossi et al. 2011).

*Augoderia*, though maintained as a valid genus since Burmeister (1847), is poorly defined and diagnosed in the literature. The irregularly spaced punctures of the elytra and the mother-of-pearl sheen of some taxa are the only characters historically used to separate *Augoderia* from *Cyclocephala*. Thus, the genus has no clearly hypothesized synapomorphic characters. For example, many characters used to diagnose *Augoderia* in Endrődi’s (1985a) *Dynastinae of the World* are all variably present in *Cyclocephala*, *Arriguttia*, and *Aspidolea* species: 1) body short, convex; 2) dorsal coloration yellow, with dark maculae, and with or without metallic reflections; 3) mandibles of males with small anterolateral tooth, lacking in females; 4) frontoclypeal suture complete; 5) 10-segmented antennae with a short club in both sexes; 6) large eyes; 7) males with thickened protarsi; and 8) protibia tridentate in both sexes.

The following combination of characters can be used to recognize *Augoderia* species: 1) dorsal coloration yellowish or light brown, with or without elytral maculae, with or without metallic, mother-of-pearl sheen; 2) body not anteroposteriorly compressed or dorsoventrally flattened; 3) clypeal apex evenly rounded in dorsal view; 4) frons mesad of eyes with long, erect setae; 5) frontoclypeal suture complete; 6) males with anterolateral margin of mandibles weakly toothed; 7) mandibular molar area with rows of circular micropunctures; 8) apical margin of mentum weakly emarginated; 9) galea of the maxilla on inner surface with 3 fused basal teeth, a free median tooth, and 2 fused apical teeth (3-1-2 arrangement); 10) pronotum at base with incomplete or complete marginal bead; 11) pronotum on anterolateral portions with long, erect setae; 12) males and females with 3 protibial teeth, basal tooth reduced, removed from the apical 2 teeth, and oriented anteriorly; 13) protibial spur straight to weakly deflexed; 14) males with inner protarsal claw enlarged and narrowly cleft at apex; 15) mesocoxae widely separated; 16) metatibiae without distal, transverse carinae; 17) metacoxae with lateral edge perpendicular to ventral surface; 18) anterior edge of hindwing distal to apical hinge lacking setae and with produced, membranous border; 19) vein RA with 2 rows of pegs extending distally nearly to margin of apical hinge.

#### Chalepides

Taxon classificationAnimaliaColeopteraScarabaeidae

Casey, 1915

##### Type species.

Parachalepus (Chalepides) eucephalus Casey, 1915, by original designation.

##### Valid taxa.

15 species.

The nomenclatural history of *Chalepides* was complicated by a case of homonymy. *Chalepides* was originally proposed as a subgenus of *Parachalepus* (Casey 1915). *Parachalepus* Casey, 1915 is a homonym of *Parachalepus* Baly, 1885 (Coleoptera: Chrysomelidae) (Prell 1936, Arrow 1937a). To rectify this problem, *Chalepides* was elevated to the status of genus and comprised the seven species originally included in *Parachalepus* (Casey 1915, Prell 1936, Arrow 1937a). *Parachalepus* was proposed based on abdominal characters. *Parachalepus* included *Dyscinetus*-like species with a rigid fusion of the propygidium and the pygidium (Casey 1915). The subgenus Parachalepus (Chalepides) was proposed for species with a dramatic reduction of the pygidium in addition to propygidial/pygidial fusion (Casey 1915). *Chalepides* has been recognized as a valid genus by subsequent authors and was recently revised (Arrow 1937a, b, Endrődi 1966, 1985a, Joly and Escalona 2002).

**Figure 56.**
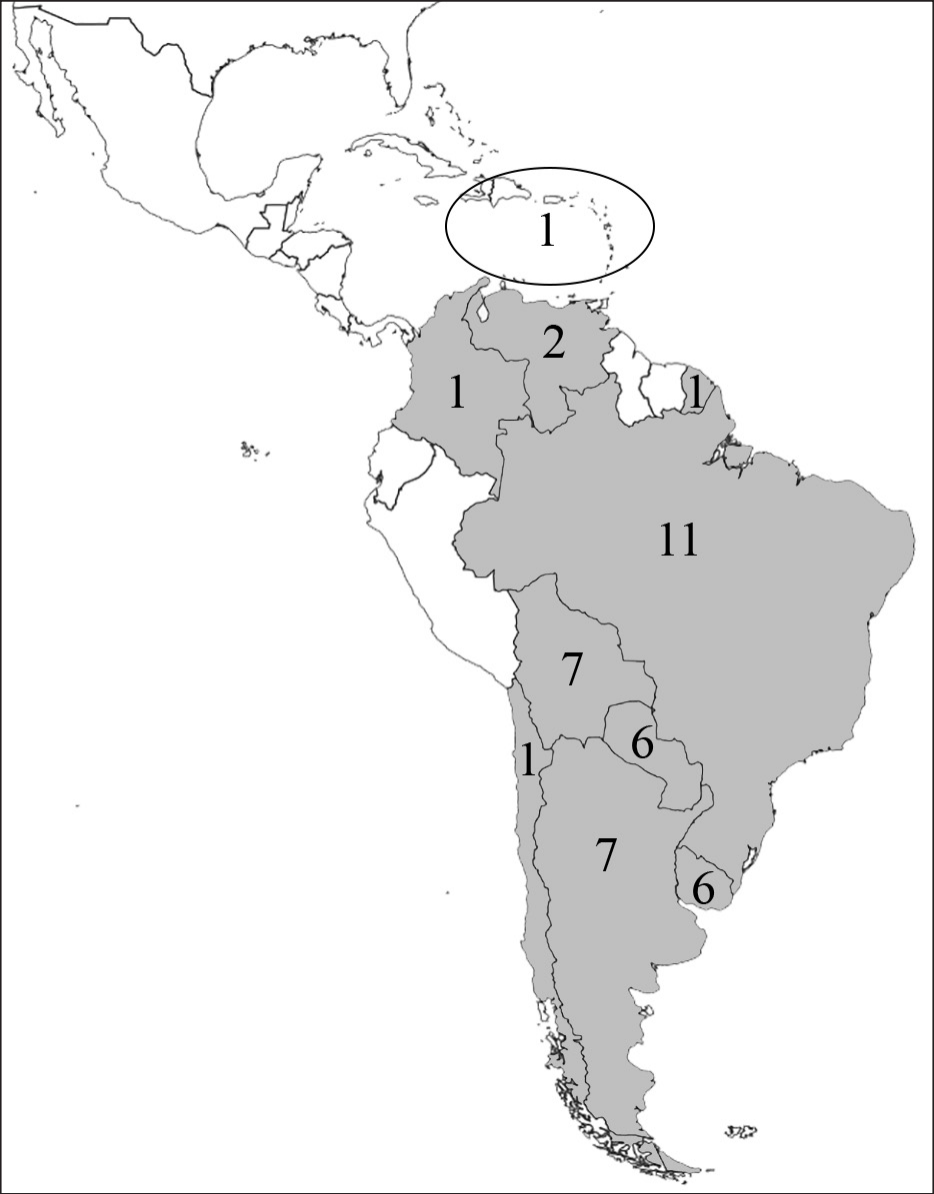
Country-level distribution of *Chalepides* taxa in South America and the West Indies. Numbers indicate taxa per country or region.

The 15 species of *Chalepides* are distributed across South America and the West Indies (Martínez 1978b, Endrődi 1966, 1973a, 1985a, Joly and Escalona 2002, Riehs 2005, Ratcliffe and Cave 2015) (Fig. 56). Species of *Chalepides* described by Prokofiev (2012) require a special discussion. *Chalepides euhirtus* Prokofiev and *C. unduavicus* Prokofiev were described based on specimens from Peru and Bolivia (Prokofiev 2012), and the Peruvian data would represent a new country record for *Chalepides*. However, both species were placed into the wrong genus, based on the original descriptions and images of the holotypes. The holotype of *C. euhirtus* appears to be a female specimen of *A. fuliginea* (Prokofiev 2012). *Chalepides unduavicus* was later synonymized under *A. scarabaeoides* and was also considered an infrasubspecific (“ab.”) entity (Prokofiev 2013, 2014). The discussion below covering the biology and genus-level recognition of *Chalepides* will exclude information on the misclassified species *C. euhirtus* and *C. unduavicus*.

Relatively little is known about the biology and natural history of *Chalepides* species. It is unclear, based on available data, if *Chalepides* species are floral visitors. Mannerheim (1829) reported that *C. dilatatus* (Mannerheim) was collected in flowers without further detail. Valla and Cirino (1972) reported a single specimen of an unidentified *Chalepides* species from the inflorescence of a *Victoria cruziana* A.D. Orb. *Chalepides barbatus* adults and larvae are associated with sugar cane fields in Puerto Rico (Wolcott 1923, 1948). In Puerto Rico, adult *C. barbatus* are prey for the invasive cane toad *R. marina* (Wolcott 1937, 1948). Like *Dyscinetus*, *Chalepides* species may have some semi-aquatic habits. *Chalepides luridus* (Burmeister) and *C. alliaceus* (Burmeister) have been collected along the edges of river banks (Endrődi 1973a). *Chalepides barbatus* reportedly attacks the invasive, aquatic weed water hyacinth (*Eichhornia crassipes* [Mart.] Solms [Pontederiaceae]) in Uruguay (Silveira Guido 1965, Perkins 1974, Buckingham and Bennett 1989). *Chalepides* species are attracted to lights at night (Kusui 1992, Riehs 2005, Albuquerque et al. 2016).

*Chalepides* species can be recognized by the following combination of characters: 1) dorsal coloration yellowish brown, dark brown, or almost black with greenish reflections in some species; 2) body convex, not strongly anteroposteriorly compressed or dorsoventrally flattened; 3) clypeus trapezoidal with apex truncate in dorsal view; 4) frontoclypeal suture complete or narrowly incomplete medially; 5) males with anterolateral margin of the mandibles lacking weak tooth; 6) mandibular molar area with rows of circular micropunctures; 7) mandibular molar area on proximal margin with 2 semicircular depressed pits; 8) galea of maxilla on inner surface with 2 fused basal teeth, 2 free medial teeth, and 2 fused apical teeth (2-2-2 arrangement); 9) pronotum with broadly incomplete beaded basal margin; 10) males and females with 3 protibial teeth on lateral margin, basal tooth not greatly reduced, only slightly removed from apical 2 teeth, and oriented laterally; 11) protibial spur straight to weakly deflexed; 12) males with inner protarsal claw enlarged and entire at apex, not cleft; 13) mesocoxae not widely separated, nearly touching; 14) metacoxae on lateral edge with transverse, depressed sulcus; 15) metacoxae with lateral edge perpendicular to ventral surface; 16) meso- and metatibiae with distal, transverse carinae; 17) anterior edge of hindwing distal to apical hinge with erect setae and lacking produced, membranous border; 18) vein RA with single row of pegs proximal to the apical hinge; 19) propygidium expanded, propygidium and pygidium fused, pygidium with long, dense setae.

#### Cyclocephala

Taxon classificationAnimaliaColeopteraScarabaeidae

Dejean, 1821

##### Type species.

*Scarabaeus amazonus* Linnaeus, 1767: 551, subsequent designation by Casey (1915).

##### Valid taxa.

359 species and subspecies.

**Figure 57.**
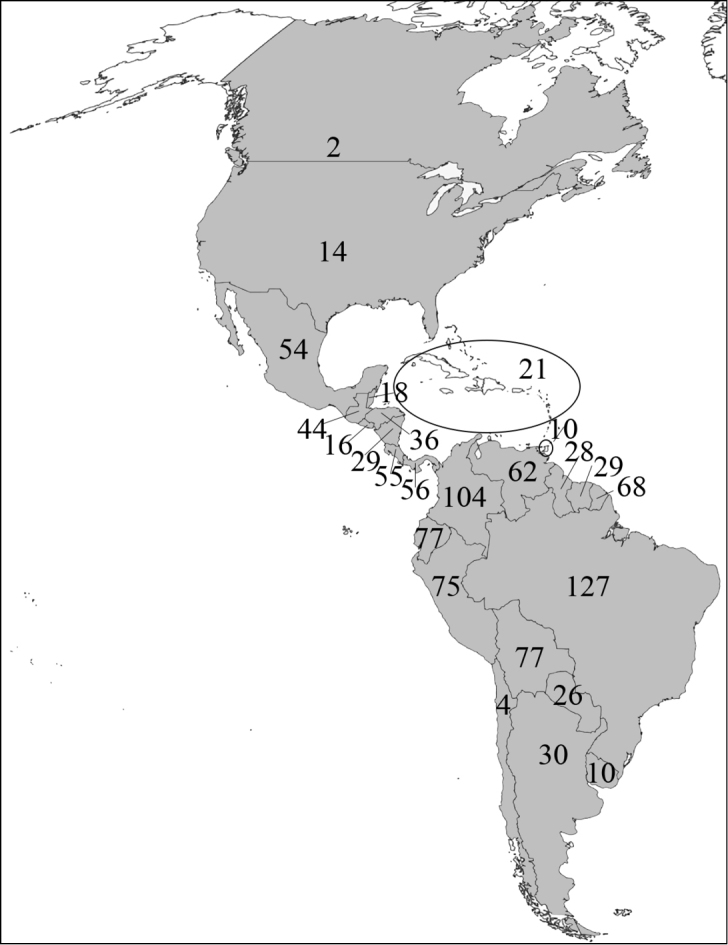
Country-level distribution of *Cyclocephala* taxa in the Neotropical and Neartic realms. Numbers indicate number of taxa per country or region.

The speciose genus *Cyclocephala* contains over 350 taxa distributed throughout the Nearctic and Neotropical realms (Fig. 57). *Cyclocephala* contains the only adventive species in Cyclocephalini, with *C. pasadenae* and *C. signaticollis* established in Hawaii and Australia, respectively (Carne 1956, Jameson et al. 2009). The greatest number of *Cyclocephala* species is found in northern South America, but many endemic species occur in Meso- and Central America. Some *Cyclocephala* species are extremely geographically widespread. For example, *C. lunulata* occurs from the southwestern United States south to Argentina. In contrast, there are also cases of endemism in mainland species of the genus. The pollination mutualist *C. jalapensis* occurs only in a narrow band of habitat in eastern Mexico (Veracruz, Puebla, Oaxaca, Querétaro, and Hidalgo states) where its host plant *Magnolia schiedeana* Schltl. is found (Dieringer and Delgado 1994, Dieringer and Espinosa 1994).

*Cyclocephala* is a difficult genus to diagnose due to its species richness, diversity of forms, and probable non-monophyly. Many of the character descriptions below are complicated by these factors. *Cyclocephala* species can be recognized by the following combination of characters: 1) dorsal coloration highly variable; unicolored black, green, or light brown, pronotum in some species cherry-red, light brown species often have complex maculae patterns of the pronotum and elytra; 2) body not anteroposteriorly compressed or dorsoventrally flattened; 3) clypeal apex variable; evenly rounded, parabolic, acute, emarginate, triemarginate, or nearly straight; 4) frons mesad of eyes with or without long, erect setae; 5) frontoclypeal suture complete or incomplete medially; 6) males with anterolateral margin of mandibles weakly toothed or not; 7) mandibular molar area with rows of circular micropunctures either present or absent; 8) apical margin of mentum weakly emarginated or broadly and deeply emarginated; 9) galea of the maxilla well-developed [with or without teeth] or reduced into a rounded process; 10) galea of the maxilla dorsoventrally flattened or not; 10) galea of maxilla on inner surface variable (not all character states are given here); with 3 fused basal teeth, a free median tooth, and 2 fused apical teeth (3-1-2 arrangement) (in *C. amazona*-like species and former *Mimeoma*, the galea are flattened and the basal tooth is compressed and rotated, giving the appearance of being bidentate with the third tooth shifted dorsally); with 2 fused basal tooth and 2 fused apical teeth (2-0-2 arrangement); with 2 fused basal teeth, 1 middle tooth, and 2 fused apical teeth (2-1-2 arrangement); 11) pronotum at base with incomplete or complete marginal bead; 12) pronotum on anterolateral portions with or without long, erect setae; 13) males with 2 or 3 protibial teeth, females always with 3; 14) protibial spur straight to weakly deflexed or strongly decurved; 15) males with inner protarsal claw enlarged and narrowly cleft at apex or entire at apex; 16) mesocoxae widely separated or nearly touching, contiguous; 17) metatibiae with or without distal, transverse carinae; 18) metacoxae with lateral edge perpendicular to ventral surface or with lateral edge angled underneath the ventral surface; 19) anterior edge of hindwing distal to apical hinge lacking setae and with produced, membranous border or lacking membranous border and with decumbent setae (*C. cribrata* species-group); 20) vein RA with 2 rows of pegs extending distally nearly to margin of apical hinge.

#### Dyscinetus

Taxon classificationAnimaliaColeopteraScarabaeidae

Harold, 1869

##### Type species.

*Melolontha geminata* Fabricius, 1801, by monotypy.

##### Valid taxa.

21 species.

**Figure 58.**
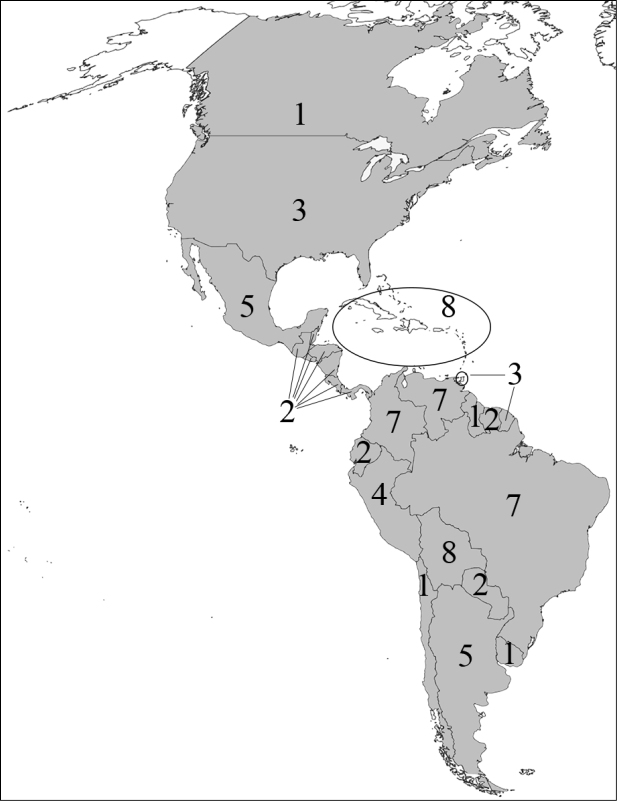
Distribution of *Dyscinetus* species in North, Central, and South America and the West Indies. Numbers indicate number of taxa per country or region.

The genus *Dyscinetus* comprises 21 species distributed from North America south to Argentina and the West Indies (Fig. 58). Smooth, large, and mostly black *Dyscinetus* species superficially resemble hydrophilid beetles. *Dyscinetus* is generally not considered an aquatic or semiaquatic genus. However, some species in the genus have an intriguing association with moist, mucky soils and aquatic plants. *Dyscinetus rugifrons* and another *Dyscinetus* sp. attack water hyacinth in Uruguay (Silveira Guido 1965, Bennett and Zwolfer 1968, Perkins 1974). *Dyscinetus morator* also attacks water hyacinth in Florida (Perkins 1974, Buckingham and Bennett 1989). These species are considered scavengers and enhancers of damage started by other arthropods on water hyacinth, though they are known to attack healthy tissues (Perkins 1974, Buckingham and Bennett 1989). Feeding damage on water hyacinth occurs inside petioles, crowns, petiole bases, and submerged roots (Perkins 1974, Buckingham and Bennett 1989).

Experiments indicated that *D. morator* can survive submerged in water for up to 36 hours (Buckingham and Bennett 1989). The mechanism allowing for this prolonged submersion is unclear. Air bubbles are visible along the elytral margin and on the metathorax in submerged individuals (Buckingham and Bennett 1989). *Dyscinetus laevipunctatus* Bates was also observed submerged in association with water hyacinth in Mexico (García-Rivera and Contreras-Ramos 2015). Unlike many other genera in the group, *Dyscinetus* adults are not known to visit flowers. A Brazilian *Dyscinetus* species was reportedly attracted to the floral odors of *Annona* sp., although these beetles were not encountered in any inflorescences (Gottsberger 1989). This is the only mention of *Dyscinetus* floral attraction in the literature.

*Dyscinetus* species can be recognized by the following combination of characters: 1) dorsal coloration dark piceous to black; 2) body convex, not strongly anteroposteriorly compressed or dorsoventrally flattened; 3) clypeus trapezoidal with apex truncate in dorsal view; 4) frontoclypeal suture complete medially; 5) males with anterolateral margin of the mandibles lacking weak tooth; 6) mandibular molar area with rows of circular micropunctures; 7) mandibular molar area on proximal margin with 2 semicircular depressed pits; 8) galea of maxilla on inner surface with 2 fused basal teeth, 2 free medial teeth, and 2 fused apical teeth (2-2-2 arrangement); 9) pronotum with broadly incomplete beaded basal margin; 10) males and females with 3 protibial teeth on lateral margin, basal tooth not greatly reduced, only slightly removed from the more apical 2 teeth, and oriented laterally; 11) protibial spur straight to weakly deflexed; 12) males with inner protarsal claw enlarged and narrowly cleft at apex; 13) mesocoxae not widely separated, nearly touching; 14) metacoxae on lateral edge with transverse, depressed sulcus; 15) metacoxae with lateral edge perpendicular to ventral surface; 16) meso- and metatibiae with distal, transverse carinae; 17) anterior edge of hindwing distal to apical hinge with erect setae and lacking produced, membranous border; 18) vein RA with single row of pegs proximal to apical hinge; 19) propygidium not expanded, with propygidium and pygidium not fused.

#### Erioscelis

Taxon classificationAnimaliaColeopteraScarabaeidae

Burmeister, 1847

##### Type species.

*Apogonia emarginata* Mannerheim, 1829, by monotypy.

##### Valid taxa.

Five species.

**Figure 59.**
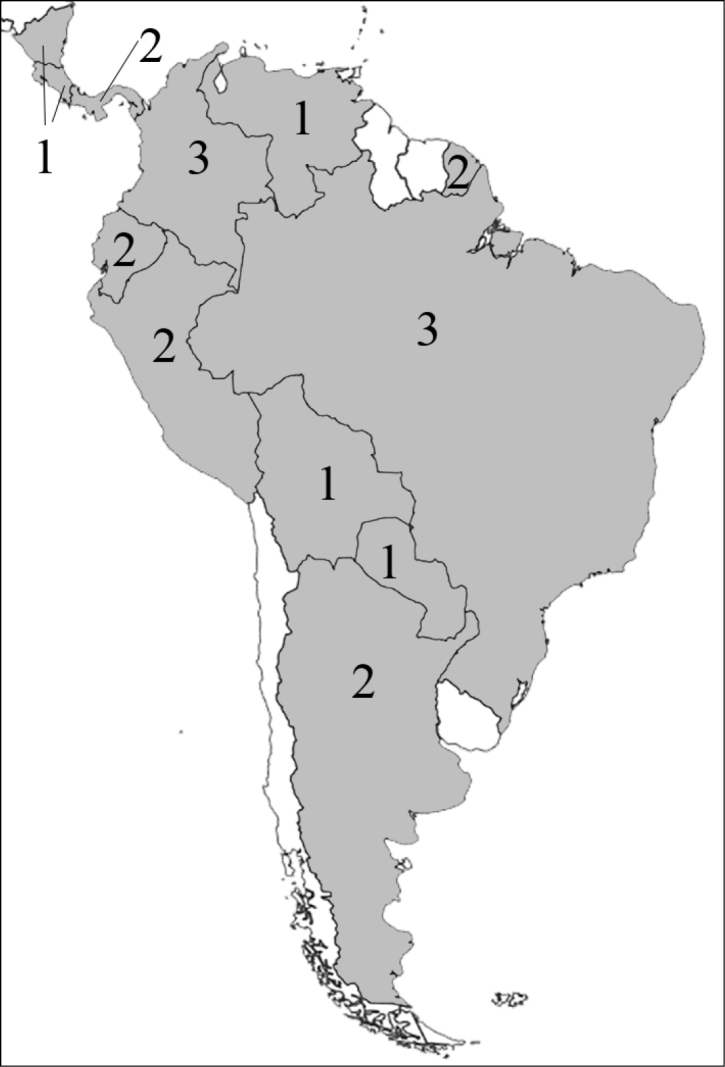
Country-level distribution of *Erioscelis* species in Central and South America. Numbers indicate taxa per country.

The five species of *Erioscelis* are distributed in South America north to Nicaragua (Fig. 59). *Erioscelis* species are remarkable among cyclocephalines for their well-characterized floral visitation syndromes. *Erioscelis* species are associated with nocturnally blooming genera in the family Araceae. Three *Erioscelis* species have been reported from the spathes of *Dieffenbachia*, *Philodendron* Schott, *Syngonium* Schott, *Montrichardia* Crueg., and possibly *Xanthosoma* Schott (Schrottky 1910, Gottsberger and Amaral 1984, Young 1986, Grayum 1996, Croat 1997, Morón 1997, Beath 1998, 1999, Gibernau et al. 2003). While the association between *Erioscelis* species and aroid flowers is firmly established, there is little evidence of species- or genus-level specificity in this pollination mutualism. For example, *Erioscelis columbica* Endrődi has been collected from the spathes of nine different *Philodendron* species in Heredia, Costa Rica (Grayum 1996, Croat 1997, Morón 1997, Moore and Jameson 2013). Based on feeding damage to *Philodendron* inflorescences by *Erioscelis*, it was hypothesized that this genus may be an interloper on the cyclocephaline/aroid mutualism (Goldwasser 1987). Other observations seem to indicate that *Erioscelis* species are part of this mutualism.

The descriptions of *Erioscelis* spp. visitation of *Dieffenbachia* and *Philodendron* inflorescences are some the most detailed available for Cyclocephalini. In Costa Rica, *E. columbica* is a pollinator of *Dieffenbachia nitidipetiolata* Croat & Grayum (Young 1986, 1988a, 1988b, 1990). *Erioscelis columbica* arrive at receptive female-phase inflorescences during nightfall, where they feed on staminodia and mate (Young 1986). The beetles exit the spathe after 24 hours when the spadix is in the male-phase and shedding pollen (Young 1986). *Erioscelis columbica* are covered in sticky pollen grains while exiting the spathe, and they may also feed on some of the pollen (Young 1986). *Erioscelis proba* (Sharp) displays similar behavior in the inflorescences of two other *Dieffenbachia* species in French Guiana (Gibernau 2015a).

Observational and experimental evidence suggests that *Erioscelis emarginata* (Mannerheim) prefers to feed upon sterile staminate flowers on the spadix in two *Philodendron* species (Maldonado et al. 2015). Furthermore, analyses of nutritional and defensive compound (calcium oxalate) content of sterile and fertile flowers in these *Philodendron* species suggested that sterile staminate flowers have lower amounts of defensive compounds (Maldonado et al. 2015). *Erioscelis* species are seemingly attracted to the strong floral scents that are volatilized during thermogenesis and receptivity of the staminate flowers in these aroids. The dynamics of floral scent attraction are mostly unexplored for *Erioscelis*. In the case of *Philodendron adamantium* Mart. ex Schott, a single dominant flower scent compound (Dihydro-β-ionone) extracted from this species was sufficient to attract *E. emarginata* to scent traps (Pereira et al. 2014).

*Erioscelis* was first revised by Saylor (1946) and again by Endrődi (1966, 1985a). These works provide a strong foundation for species-level identification, but characters that separate *Erioscelis* from other cyclocephalines are largely undiscussed. For example, Saylor (1946) commented, “When compared with such species as Cyclocephala (Stigmalia) mafaffa Burmeister, or C. (Aclinidia) castanea (Fabricius), the only character definitely to separate *Erioscelis* is the unenlarged front tarsal claws of both sexes”. Unique protibial (2 teeth on the lateral margin in both sexes, subapical position of reduced protibial spur) and abdominal (bisinuate margin of 6^th^ abdominal sternite, terminal spiracle not positioned on pleural suture) characters of *Erioscelis emarginata* also complicate recognition of the genus and may be reasons to doubt the monophyly of the group. These characters (except for the bisinuate margin of 6^th^ abdominal sternite) are associated with Anomalini (Rutelinae) and are absent in all other members of *Erioscelis* and Cyclocephalini more broadly. Sister-relationships of *Erioscelis* have not been hypothesized and the immature stages are unknown for the genus.

*Erioscelis* species can be recognized by the following combination of characters: 1) dorsal coloration castaneous, rufocastaneous, or piceous; 2) body not dorsoventrally flattened nor anteroposteriorly compressed; 3) clypeal apex truncate, weakly emarginate, or deeply emarginate in dorsal view; 4) frontoclypeal suture complete medially; 5) apical margin of mentum shallowly emarginate; 6) anterolateral margin of mandible lacking tooth; 7) mandibular molar area with rows of circular micropunctures; 8) galea of maxilla not dorsoventrally flattened; 9) galea of maxilla on inner surface with 6 teeth in 2-2-2 arrangement (each pair shares a base); 10) pronotum with apical bead complete medially; 11) basal bead of pronotum incomplete medially; 12) anterior membrane of pronotum straight at middle, not projected anteriorly; 13) anterior membrane of the pronotum extending laterally to apicolateral margins of the pronotum; 14) protibia with 2 or 3 lateral teeth in both sexes; 15) when protibia tridentate, basal tooth not greatly reduced, only slightly removed from the apical 2 teeth, and oriented laterally; 16) protibial spur subapical or apically positioned; 17) protibial spur straight to weakly reflexed; 18) males and females with protarsal claws simple, not enlarged; 19) males and females with inner protarsal claws with apex entire, not cleft; 20) mesocoxae not widely separated, nearly touching; 21) metacoxae with lateral edge perpendicular to ventral surface; 22) anterior edge of hindwing distal to apical hinge simple (lacking setae or membrane) or with row of long, erect setae extending along vein; 23) vein RA with double row of pegs proximal to apical hinge; 24) terminal abdominal spiracle situated on pleural suture or not.

#### Harposceles

Taxon classificationAnimaliaColeopteraScarabaeidae

Burmeister, 1847

##### Type species.

*Harposceles paradoxus* Burmeister, 1847: 35, by monotypy.

##### Valid taxa.

One species.

**Figure 60.**
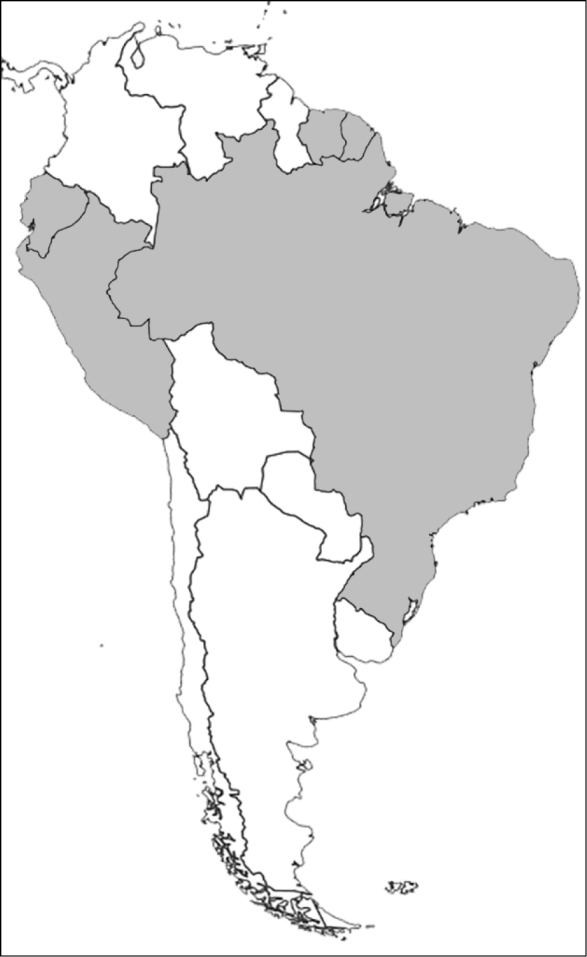
Country-level distribution of *Harposceles paradoxus* in South America.

The monotypic genus *Harposceles* was erected for the species *H. paradoxus*. This striking, relatively large cyclocephaline occurs in lowland forests in Brazil, Ecuador, French Guiana, Peru, Suriname, and possibly Colombia (Burmeister 1847, Harold 1869b, Arrow 1937b, Blackwelder 1944, Endrődi 1966, 1985a, Endrődi and Dechambre 1976, Lachaume 1992, Couturier and Kahn 1992, Andreazze 2001, Andreazze and da Silva Motta 2002, Touroult et al. 2010, Ponchel 2011, Saltin and Ratcliffe 2012, Ratcliffe et al. 2015) (Fig. 60). Males display dramatic, and unique, characters of the protibia. *Harposceles paradoxus* males have elongated, arcuate protibia with the protibial spurs fused to the base of the tibia. Females are much less common than male specimens in collections, and males are readily attracted to lights at night, especially between midnight and 4 am (Andreazze 2001, Andreazze and da Silva Motta 2002, Touroult et al. 2010, Saltin and Ratcliffe 2012). The immature stages of *H. paradoxus* are associated with the palms *Astrocaryum chonta* Mart. and *A. carnosum* F. Kahn & B. Millán (Arecaceae) (Couturier and Kahn 1992). The larvae and pupae were found in the organic litter accumulated between leaf sheaths of *A. carnosum* (Couturier and Kahn 1992). The immature stages are undescribed.

*Harposceles* species can be recognized by the following combination of characters: 1) dorsal coloration dark piceous to black; 2) body dorsoventrally flattened; 3) clypeus rounded in dorsal view; 4) frontoclypeal suture incomplete medially; 5) apical margin of mentum truncate; 6) anterolateral margin of mandible lacking tooth; 7) mandibular molar area with surface lacking circular pits, with large, disorganized, canal-like invaginations; 8) galea of maxilla dorsoventrally flattened; 9) galea on inner surface at base with large, flattened, blade-like, tooth (less produced than in *Surutu* species); 10) galea on inner surface with 7 teeth in 2-1-1-1-2 arrangement from base to apex; 11) apical and basal beaded margins of pronotum complete at middle; 12) anterior membrane of the pronotum interrupted before lateral pronotal margins; 13) males with protrochanter with ventrally produced tooth; 14) protibia with 3 teeth in both sexes; 15) males with protibia elongated and arcuate; 16) protibial spur straight to weakly reflexed; 17) males with protibial spur fused to protibia, not articulated at its base; 18) males with inner protarsal claw thickened and not cleft at apex; 19) mesocoxae not widely separated, nearly touching; 20) metacoxae with lateral edge perpendicular to ventral surface; 21) apices of the meso- and metatibiae with a corbel; 22) anterior edge of hindwing distal to apical hinge lacking membranous border; 23) anterior edge of hindwing distal to apical hinge with decumbent setae surrounding vein and originating away from apical hinge; 24) vein RA with single row of pegs proximal to apical hinge.

The relationship of *Harposceles* to other cyclocephalines has not been elaborated upon in the literature. However, *H. paradoxus* shares some characters with *Surutu* that may be indicative of a close relationship between the two genera. The rounded shape of the clypeal apex in *H. paradoxus* is like the clypeal form in *S. dytiscoides*. The single row of RA pegs in *H. paradoxus* is shared between *Ancognatha* and *Surutu*, though *Ancognatha* species lack setae on the anterior edge of the hindwing distal to the apical hinge. The decumbent setae of the hindwing leading edge (distal to apical hinge) found in *H. paradoxus* is also found in *Surutu* species and the “*Cyclocephala cribrata* species group” (which included species previously placed in *Mononidia* and *Surutoides*) (Dechambre 1997). These groups also all share corbeled meso- and metatibial apices and entirely black coloration. *Harposceles paradoxus* shares other interesting characters with *Surutu* species. These shared characters include: 1) body strongly dorsoventrally flattened; 2) dorsoventrally flattened maxillary galea; 3) a seven-toothed maxillary galea in a 2-1-1-1-2 arrangement from the base to apex; 4) an incomplete frontoclypeal suture; and 5) the apical pronotal membrane interrupted before the lateral margins of the pronotum. The large basal tooth of the maxillary galea is much smaller and less produced in *H. paradoxus* than in *Surutu* species. Several male characters of *H. paradoxus* are autapomorphic in Cyclocephalini: 1) the protibial spur fusion to the protibial; 2) the arcuate, elongated protibia (seen also in some Dynastini); and 3) the ventrally produced protrochanter teeth.

#### Peltonotus

Taxon classificationAnimaliaColeopteraScarabaeidae

Burmeister, 1847

##### Type species.

*Peltonotus morio* Burmeister, 1847: 75, by monotypy.

##### Valid taxa.

25 species.

**Figure 61.**
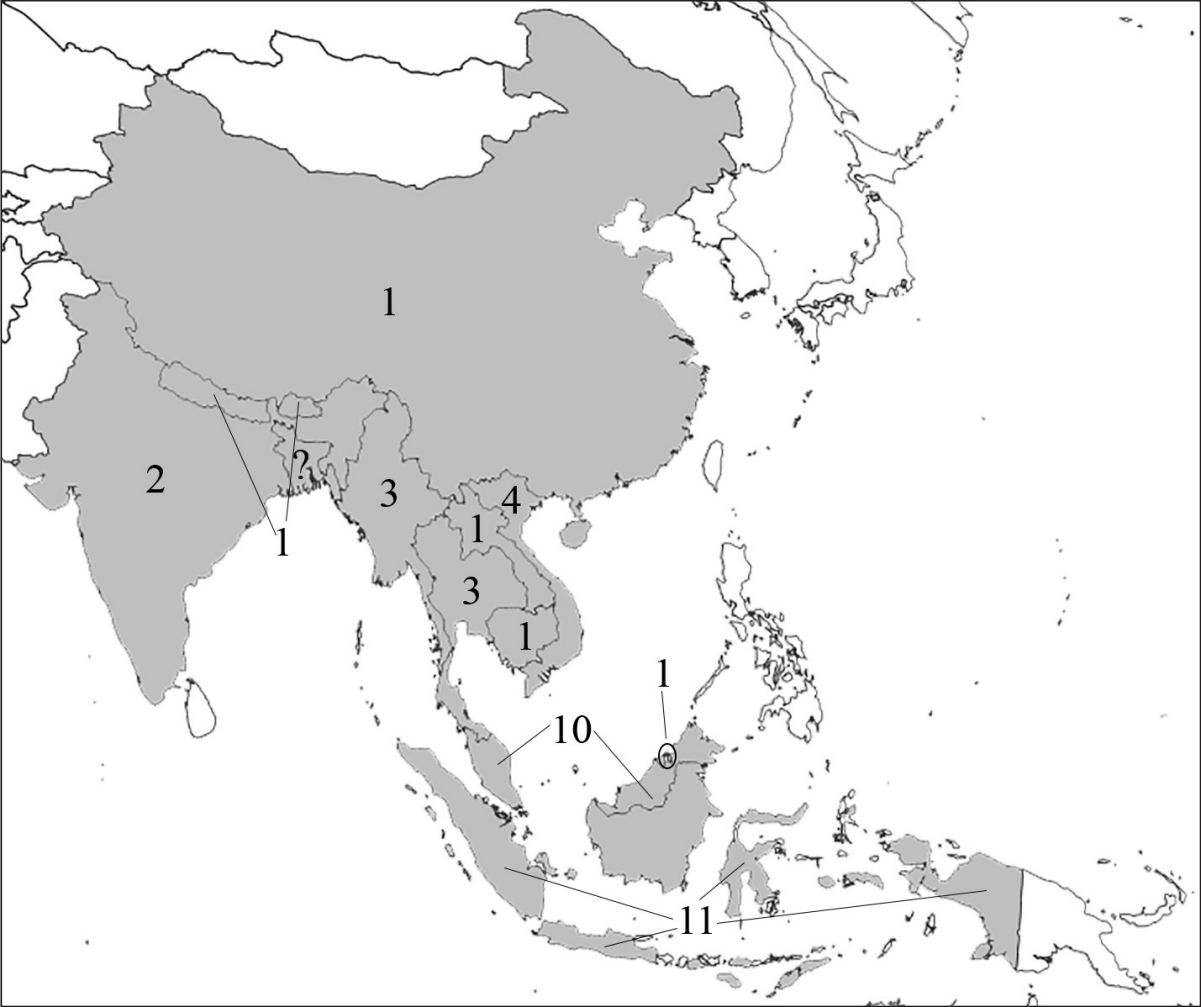
Country-level distribution of *Peltonotus* species in Southeast Asia, the Indian Subcontinent, and China. Numbers indicate taxa per country.

*Peltonotus* species are distributed throughout Southeast Asia, southern China, and the eastern portion of the Indian Subcontinent (Fig. 61). *Peltonotus* is currently considered the sole Asian lineage of Cyclocephalini, though its subfamilial classification has been unstable. The genus is remarkable for its confounding combination of morphological and behavioral traits that blurred the lines between historical concepts of the subfamilies Dynastinae and Rutelinae. For example, the sexual dimorphism of the protarsi in *Peltonotus* species has long been compared to that found in *Cyclocephala* (e.g., see Burmeister 1847). In contrast, the labral morphology of *Peltonotus* species matches that found in Asian parastasiine and fruhstoferiine (Rutelinae) scarabs (Arrow 1908, 1910). The floral feeding behavior of *Peltonotus* species on Araceae is also shared between cyclocephalines and Asian parastasiines, adding a further layer of intrigue to unresolved evolutionary relationships between the groups at the subfamilial- and tribal-level (e.g., see Moore and Jameson 2013, Kumano-Nomura and Yamaoka 2006, Kumano-Nomura and Yamaoka 2009, Tung et al. 2010, Hoe et al. 2011, 2016).

*Peltonotus* was described by Burmeister (1847), and he included it within the Chalepidae division of Cyclocephalidae. The classification of *Peltonotus* was stable until Arrow (1908, 1910) transferred the genus to Rutelinae based upon the exposed (in dorsal view, produced apically beyond the clypeus) and chitinized labrum. Arrow (1917) later erected the “division” Peltonotini for *Peltonotus* within his classification of Rutelinae. Ohaus (1918, 1934b) and Machatschke (1972) rejected Peltonotini and included *Peltonotus* in Pelidnotina (Rutelini) in their catalogs of Rutelinae. Morphological phylogenetic analysis of Rutelina (Rutelinae: Rutelini) suggested that *Peltonotus* were more closely related to Cyclocephalini than Rutelini (Jameson 1998). Subsequent works on the genus have treated *Peltonotus* as a member of Cyclocephalini (Jameson and Wada 2004, 2009, Jameson and Jákl 2010, Jameson and Drumont 2013).

Little is known about the biology and natural history of *Peltonotus* species. The immatures are undescribed. Adults are attracted to lights at night (Jameson and Wada 2004). *Peltonotus malayensis* Arrow was collected from the spathes of *Epipremnum falcifolium* Engl. (Araceae), where males and females were observed mating and feeding (Jameson and Wada 2004). In Thailand, *P. nasutus* visit the large inflorescences of the terrestrial aroid *Amorphophallus paeoniifolius* (Dennst.) Nicolson, where adult beetles feed and mate (Grimm 2009). *Peltonotus nasutus* can be attracted to the inflorescences in high numbers (over 70 individuals) (Danell 2010).

*Peltonotus* species can be recognized by the following combination of characters: 1) dorsal coloration brown to black with variable presence of maculae; 2) body convex, not dorsoventrally flattened; 3) clypeal apex rounded to straight in dorsal view; 4) frontoclypeal suture incomplete medially; 5) apical margin of mentum variably shaped with weak emargination; 6) anterolateral margin of mandible lacking tooth; 7) mandibular molar area with rows of circular micropunctures; 8) galea of maxilla not strongly dorsoventrally flattened; 9) galea of the maxilla on inner surface with 3 fused basal teeth, a free median tooth, and 2 fused apical teeth (3-1-2 arrangement); 10) galea with articulated medial tooth; 11) labrum extending apically beyond clypeal apex (obvious in dorsal view); 12) apical and basal margins of pronotum with beaded margin complete or incomplete at middle; 13) protibia of males with 2 or 3 teeth, females with 3 teeth; 14) protibial spur straight to weakly reflexed; 15) males with inner protarsal claw thickened and not cleft at apex (nib variably present or absent); 16) mesocoxae not widely separated, nearly touching; 17) metacoxae with lateral edge perpendicular to ventral surface; 18) anterior edge of hindwing distal to apical hinge lacking membranous border; 19) anterior edge of hindwing distal to apical hinge with row of long setae extending from apical hinge along length of the costal vein; 20) vein RA with single row of pegs proximal to apical hinge.

#### Ruteloryctes

Taxon classificationAnimaliaColeopteraScarabaeidae

Arrow, 1908

##### Types species.

*Ruteloryctes tristis* Arrow, 1908: 336, by monotypy.

##### Valid taxa.

Two species.

**Figure 62.**
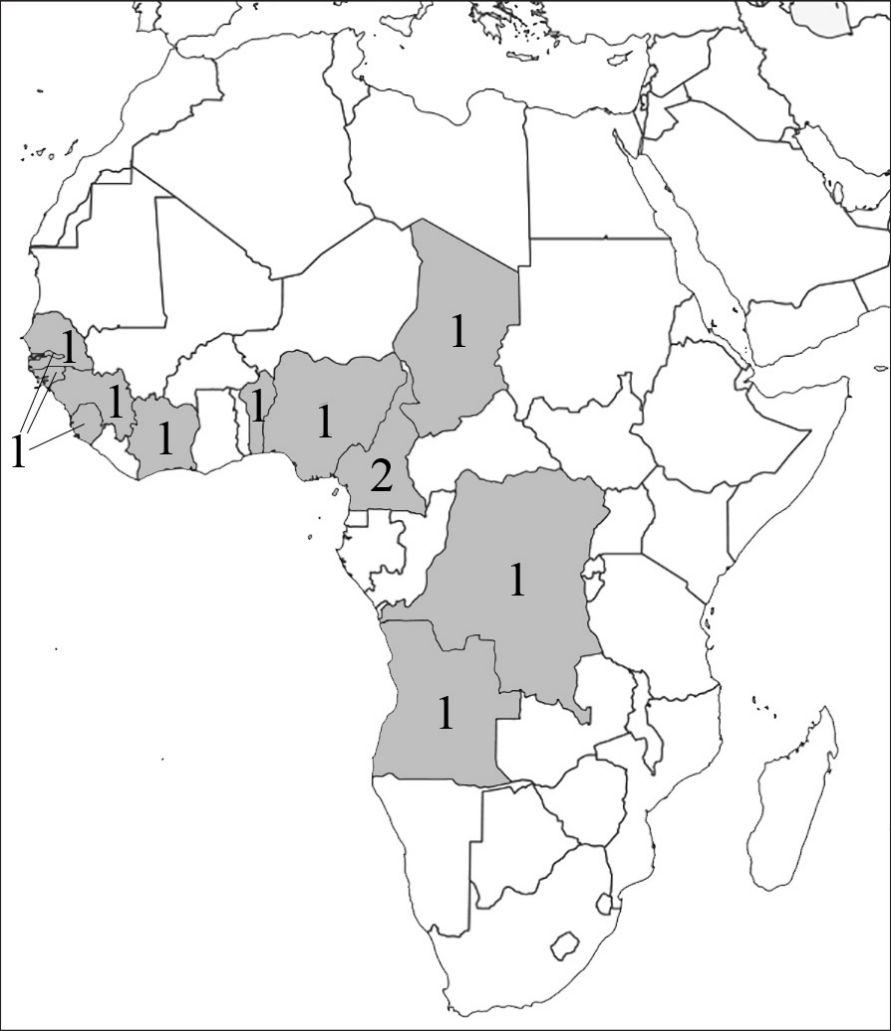
Country-level distribution of *Ruteloryctes* species in Africa. Numbers indicate taxa per country.

The two species of *Ruteloryctes* are distributed in the Guinea-Congo lowland rainforests of West and Central Africa. *Ruteloryctes* specimens have been collected in Angola, Benin, Cameroon, Chad, Côte d’Ivoire, Democratic Republic of the Congo, Guinea, Guinea-Bissau, Nigeria, Senegal, Sierra Leone, and The Gambia (Burgeon 1947, Paulian 1954, Endrődi 1960, 1966, 1985a, Krell et al. 2003, Hirthe and Porembski 2003, Ervik and Knudsen 2003) (Fig. 62). *Ruteloryctes morio* is a pollinator of nocturnally blooming *Nymphaea lotus* L., and this floral association has been reported from Côte d’Ivoire, Senegal, and Nigeria (Fabricius 1798, Krell et al. 2003, Hirthe and Porembski 2003, Ervik and Knudsen 2003). The immature stages of *Ruteloryctes* are undescribed.

*Ruteloryctes* species can be recognized by the following combination of characters: 1) dorsal coloration black to dark brown; 2) body convex, not strongly anteroposteriorly compressed or dorsoventrally flattened; 3) clypeal apex truncate or rounded in dorsal view; 4) frontoclypeal suture incomplete medially; 5) males with anterolateral margin of the mandibles lacking weak tooth; 6) mandibular molar area with rows of circular micropunctures; 7) apex of mentum weakly emarginated at middle; 8) galea of maxilla on inner surface with 3 fused basal teeth, a free median tooth, and 2 fused apical teeth (3-1-2 arrangement); 9) pronotum with broadly incomplete beaded basal margin; 10) males and females with 3 protibial teeth on lateral margin, basal tooth not greatly reduced, slightly removed from apical 2 teeth, and oriented laterally; 11) protibial spur straight to weakly deflexed; 12) males with inner protarsal claw enlarged and narrowly cleft at apex; 13) mesocoxae not widely separated, nearly touching; 14) meso- and metatibiae with distal, transverse carinae; 15) metacoxae with lateral edge perpendicular to ventral surface; 16) anterior edge of hindwing distal to apical hinge lacking setae and with produced, membranous border; 17) vein RA with single row of pegs proximal to apical hinge.

The original description of *Ruteloryctes* compared the genus to New World *Dyscinetus* species, and it was hypothesized to have “strayed across the Atlantic” (Arrow 1908). Endrődi (1966) thought that *Ruteloryctes* was one of the most “primitive” cyclocephaline genera. The 3-1-2 arrangement of the teeth on the maxillary galea in *Ruteloryctes* is most similar to *Arriguttia*, *Augoderia*, and many *Cyclocephala* species. The membranous border of the hindwing present in *Ruteloryctes* is also shared with *Arriguttia*, *Acrobolbia*, *Ancognatha*, *Aspidolea*, and *Cyclocephala*. However, the single row of pegs present on the hindwing RA vein in *Ruteloryctes* is present in *Ancognatha*, *Surutu*, *Harposceles*, *Stenocrates*, *Dyscinetus*, *Erioscelis*, and *Chalepides*.

#### Stenocrates

Taxon classificationAnimaliaColeopteraScarabaeidae

Burmeister, 1847

##### Type species.

*Scarabaeus laborator* Fabricius, subsequent designation by Casey 1915: 114.

##### Valid taxa.

52 species and subspecies.

**Figure 63.**
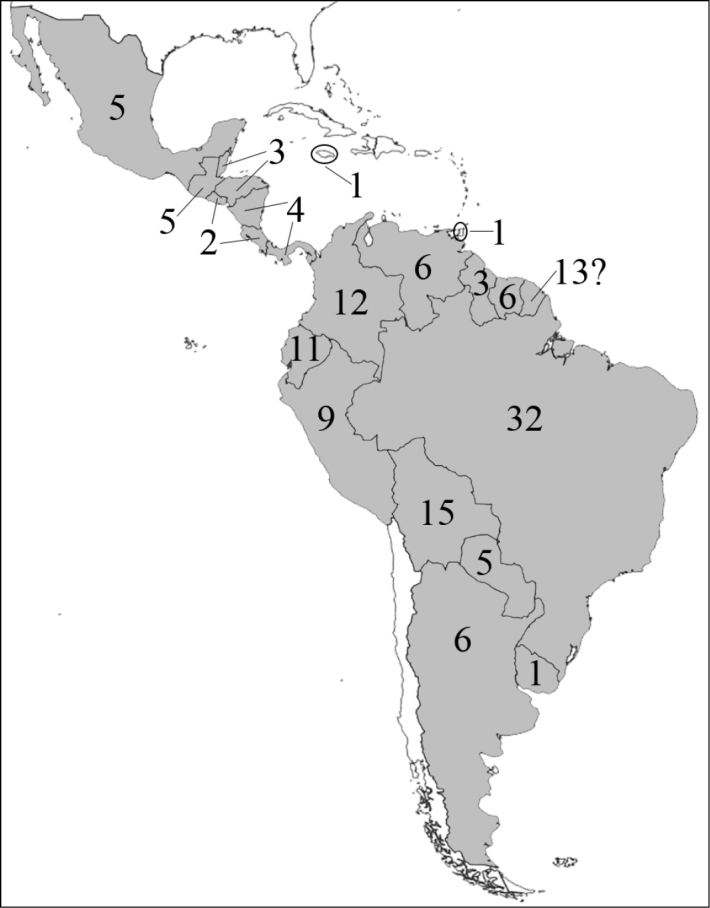
Country-level distribution of *Stenocrates* species and subspecies in Meso-, Central, and South America and the West Indies. Numbers indicate taxa per country.

The enigmatic genus *Stenocrates* comprises 52 taxa distributed from Mexico south throughout South America (except Chile) and Jamaica (Fig. 63). Species diversity in the group is highest in the tropical forests of Brazil, especially the northern and western states of Amazonas, Pará, Acre, and Rondônia. Many *Stenocrates* species are also known from eastern Brazil, especially Bahia, Espírito Santo, São Paulo, and Santa Catarina. *Stenocrates* species are problematic to identify due to conserved external morphology among species, making the group, “…possibly the most difficult genus of Dynastinae in the Americas with which to work” (Ratcliffe and Cave 2015). Male paramere morphology is diagnostic for species-level identification in the genus, and females not associated with males at the time of collection cannot be reliably identified with existing literature. Nothing is known about the natural history and biology of *Stenocrates* species. Adults can be collected at lights at night (Endrődi 1969a, Ratcliffe and Cave 2006, Ratcliffe 2014, 2015). Immature stages are undescribed for the genus.

*Stenocrates* was erected by Burmeister (1847) for species that he considered highly similar to the historical concept of *Chalepus*, except for the lack of dimorphic protarsi. Burmeister (1847) included 4 species in *Stenocrates* and speculated that *Melolontha rufipennis* Fabricius could also be a member of the genus. Descriptions of new species of *Stenocrates* were slow to accumulate in the 19^th^ and early 20^th^ century. Kirsch (1870) described the sixth *Stenocrates* species from Colombia. Bates (1888) examined *S. laborator* specimens from Mexico and noted that the simple protarsi of the males and dorsoventrally flattened tibiae separated diagnosed *Stenocrates* within Cyclocephalini. *Stenocrates* was compared to *Euetheola* by Bates (1888) stating that the form of the mandibles and the proximal tarsomeres served to separate these genera. Arrow (1911, 1913) added two new species to *Stenocrates*, but he did not offer a diagnosis for the genus or make meaningful character comparisons for the genus. *Stenocrates* was revised by Endrődi (1966, 1985a), and many new species have been described since that work, which have not been incorporated into a comprehensive identification key.

*Stenocrates* species can be recognized by the following combination of characters: 1) dorsal coloration black or dark brown and without maculae; 2) body convex, not strongly anteroposteriorly compressed or dorsoventrally flattened; 3) clypeus trapezoidal with apex truncate in dorsal view; 4) frontoclypeal suture complete medially; 5) males with anterolateral margin of the mandibles lacking weak tooth; 6) mandibular molar area with rows of circular micropunctures; 7) mandibular molar area on proximal margin without semicircular depressed pits; 8) galea of maxilla on inner surface with 2 fused basal teeth, 2 fused medial teeth, and 2 fused apical teeth (2-2-2 arrangement); 9) pronotum with broadly incomplete beaded basal margin; 10) pronotum with narrowly incomplete beaded apical margin; 11) males and females with 3 protibial teeth on lateral margin, basal tooth not greatly reduced, only slightly removed from apical 2 teeth, and oriented laterally; 12) protibial spur straight to weakly deflexed; 13) males and females with protarsal claws simple (not cleft) and not enlarged; 14) mesocoxae not widely separated, nearly touching; 15) metacoxae on lateral edge without transverse, depressed sulcus; 16) metacoxae with lateral edge perpendicular to ventral surface; 17) meso- and metatibiae with distal, transverse carinae; 18) meso- and metatibiae dorsoventrally flattened and laterally expanded; 19) anterior edge of hindwing distal to apical hinge with erect setae and lacking produced, membranous border; 20) vein RA with single row of pegs proximal to apical hinge; 21) propygidium not expanded, propygidium and pygidium not rigidly fused.

#### Surutu

Taxon classificationAnimaliaColeopteraScarabaeidae

Martínez, 1955

##### Type species.

*Surutu dytiscoides* Martínez, 1955: 245–249, by monotypy.

##### Valid taxa.

Five species.

**Figure 64.**
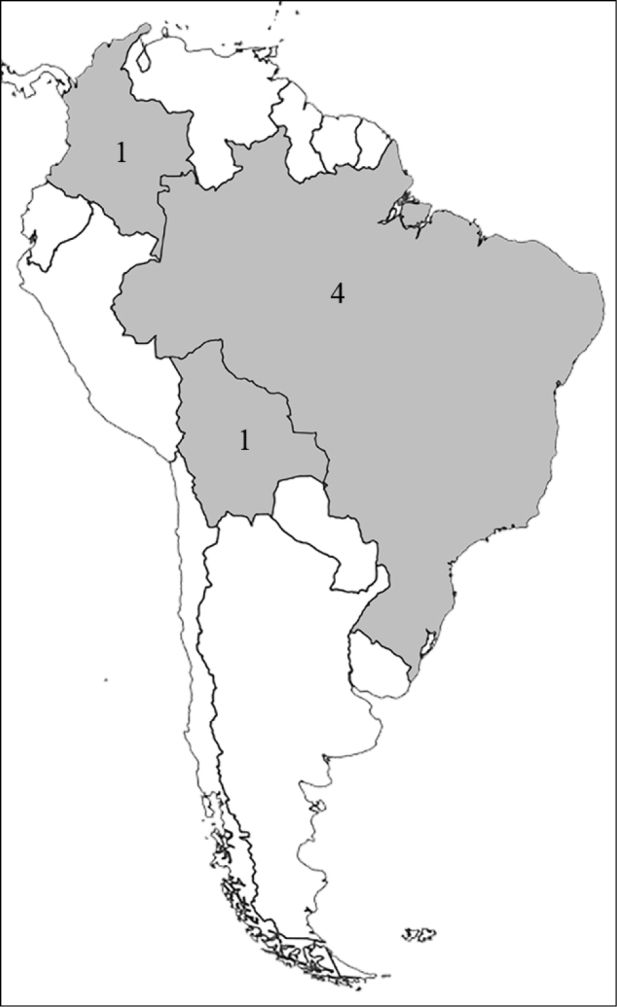
Country-level distribution of *Surutu* species in South America. Numbers indicate taxa per country.

The five species of the South American genus *Surutu* are distributed in Colombia, Bolivia, and Brazil (Martínez 1955, D’Andretta and Martínez 1956, Endrődi 1966, 1975a, 1985a, Ratcliffe 1981, Andreazze 2001, Otavo et al. 2013) (Fig. 64). These spectacular black species are truly the monsters of the Cyclocephalini, with some specimens of *Surutu seabrai* D’Andretta and Martínez measuring over 4 cm in length. Nothing is known about the biology of *Surutu* species. At least some species are attracted to lights at night (Ratcliffe 1981). The immature stages are undescribed for the genus as currently circumscribed.

*Surutu* species can be recognized by the following combination of characters: 1) dorsal coloration dark piceous to black; 2) body dorsoventrally flattened; 3) clypeus rounded to parabolic in dorsal view; 4) frontoclypeal suture incomplete medially; 5) apex of mentum narrowly and deeply emarginated (in *S. dytiscoides* and *S. seabrai*; other species unknown); 6) anterolateral margin of mandible lacking tooth; 7) galea of maxilla dorsoventrally flattened (in *S. dytiscoides* and *S. seabrai*; other species unknown); 8) galea on inner surface at base with large, flattened, blade-like, bifurcated tooth (in *S. dytiscoides* and *S. seabrai*; other species unknown); 9) galea on inner surface with 7 teeth in 2-1-1-1-2 arrangement from base to apex (in *S. dytiscoides* and *S. seabrai*; other species unknown); 10) apical and basal beaded margins of pronotum incomplete at middle (in *S. dytiscoides* and *S. seabrai*; other species unknown); 11) anterior membrane of the pronotum interrupted before lateral pronotal margins (in *S. dytiscoides* and *S. seabrai*; other species unknown); 12) protibia with 3 teeth in both sexes; 13) protibial spur straight to weakly reflexed; 14) males with protibial spur articulated at base, not fused to protibia; 15) males with inner protarsal claw thickened and narrowly cleft at apex (claw apex entire in *S. fenni* Ratcliffe and *S. schulzei* Endrődi); 16) mesocoxae not widely separated, nearly touching; 17), metacoxae with lateral edge perpendicular to ventral surface; 18) apices of the meso- and metatibiae with a corbel (in *S. dytiscoides* and *S. seabrai*; other species unknown); 19) anterior edge of hindwing distal to apical hinge lacking membranous border; 20) anterior edge of hindwing distal to apical hinge with decumbent setae surrounding the vein and originating away from the hinge; 21) vein RA with single row of pegs proximal to apical hinge.

Some characters of the head, mouthparts, and elytra of *Surutu* have been compared to *Ancognatha*, *Cyclocephala*, and *Mimeoma* (Martínez 1955, D’Andretta and Martínez 1956). The parabolic and rounded clypeal apex in *Surutu* species is like the clypeal form in several *Ancognatha* species. *Surutu dytiscoides* and *S. seabrai*, at least, have a deeply emarginated apex of the mentum that is also shared with *Ancognatha* species. The single row of RA pegs is also shared between *Ancognatha* and *Surutu*, although *Ancognatha* species lack setae on the anterior edge of the hindwing distal to the apical hinge. Instead, *Ancognatha* have a hindwing membrane like that found in *Cyclocephala*, *Augoderia*, *Arriguttia*, *Aspidolea*, and *Acrobolbia*. The dramatic dilations and knobs on the elytral epipleuron of *S. seabrai* are similar to those found in some *Ancognatha* and *Cyclocephala* species.

The distinctive setae of the hindwings found in *Surutu* are also found in *Harposceles* and species of the “*Cyclocephala cribrata* species group” (which included species previously placed in the genera *Mononidia* and *Surutoides*) (Dechambre 1997). These groups also share corbeled meso- and metatibial apices and entirely black coloration. *Harposceles paradoxus* shares other interesting characters with *Surutu* species, suggestive of a close relationship between the two genera. These shared characters include: 1) body strongly dorsoventrally flattened; 2) dorsoventrally flattened maxillary galea; 3) a 7-toothed maxillary galea in a 2-1-1-1-2 arrangement from the base to apex; 4) an incomplete frontoclypeal suture; and 5) the apical pronotal membrane interrupted before the lateral pronotal margins.

*Platyphileurus felscheanus* Ohaus (Dynastinae: Oryctini) warrants special discussion here. This species was described twice. *Platyphileurus felscheanus* was described from specimens collected from Santa Catarina, Brazil (Ohaus 1910). This new genus was compared to *Phileurus* Latreille and later included in the tribe Phileurini (Ohaus 1910, Arrow 1937b). Endrődi (1975) later described *Surutu jelineki* from Rio de Janeiro based on two female specimens. Comparison of the types of these species revealed that they are conspecific, with the name *Platyphileurus felscheanus* having priority over *Surutu jelineki* (Grossi et al. 2010).

The immatures of *Platyphileurus felscheanus* are associated with bromeliads (Grossi et al. 2010, Albertoni et al. 2014). Based on examination of larval, pupal, and adult characters, *P. felscheanus* was excluded from Phileurini and proposed to be a member of Oryctini (Albertoni et al. 2014). However, there are some intriguing adult character similarities between *P. felscheanus* and other *Surutu* species. For example, *P. felscheanus* is black, dorsoventrally flattened, and has dimorphic protarsal claw morphology (enlarged in males, simple in females) (Endrődi 1975, Grossi et al. 2010, Albertoni et al. 2014). The apices of the metatibiae in *P. felscheanus* are “weakly dentate” (Albertoni et al. [2014]: figure 30). Alternatively, the outer edge of the metatibia figured in Albertoni et al. (2014) could be considered not to be “weakly dentate”, but corbeled (outer edge produced beyond the inner edge of the tibial apex). This tibial character is found in *Surutu*, *Harposceles*, and in the “*Cyclocephala cribrata* species group”. The venter of the meso- and metatarsi in *P. felscheanus* is covered with dense, reddish, flattened setae (Albertoni et al. 2014). Similar flattened, scale-like setae are also found on the venter of the meso- and metatarsi of *S. seabrai* and *S. dytiscoides*. Future analyses of the tribal placement of *P. felscheanus* should focus on adult character comparisons with *Surutu* species and *H. paradoxus*, especially characters of the mandibles, maxillary galea, tibiae, tarsi, parameres, and hind wings.

## Supplementary Material

XML Treatment for Acrobolbia

XML Treatment for Ancognatha

XML Treatment for Arriguttia

XML Treatment for Aspidolea

XML Treatment for Augoderia

XML Treatment for Chalepides

XML Treatment for Cyclocephala

XML Treatment for Dyscinetus

XML Treatment for Erioscelis

XML Treatment for Harposceles

XML Treatment for Peltonotus

XML Treatment for Ruteloryctes

XML Treatment for Stenocrates

XML Treatment for Surutu
